# An Upper Palaeolithic engraved human bone associated with ritualistic cannibalism

**DOI:** 10.1371/journal.pone.0182127

**Published:** 2017-08-09

**Authors:** Silvia M. Bello, Rosalind Wallduck, Simon A. Parfitt, Chris B. Stringer

**Affiliations:** 1 Department of Earth Sciences, The Natural History Museum, London, United Kingdom; 2 University College London, Institute of Archaeology, London, United Kingdom; Max Planck Institute for the Science of Human History, GERMANY

## Abstract

Cut-marked and broken human bones are a recurrent feature of Magdalenian (~17–12,000 years BP, uncalibrated dates) European sites. Human remains at Gough’s Cave (UK) have been modified as part of a Magdalenian mortuary ritual that combined the intensive processing of entire corpses to extract edible tissues and the modification of skulls to produce skull-cups. A human radius from Gough’s Cave shows evidence of cut marks, percussion damage and human tooth marks, indicative of cannibalism, as well as a set of unusual zig-zagging incisions on the lateral side of the diaphysis. These latter incisions cannot be unambiguously associated with filleting of muscles. We compared the macro- and micro-morphological characteristics of these marks to over 300 filleting marks on human and non-human remains and to approximately 120 engraved incisions observed on two artefacts from Gough’s Cave. The new macro- and micro-morphometric analyses of the marks, as well as further comparisons with French Middle Magdalenian engraved artefacts, suggest that these modifications are the result of intentional engraving. The engraved motif comfortably fits within a Magdalenian pattern of design; what is exceptional in this case, however, is the choice of raw material (human bone) and the cannibalistic context in which it was produced. The sequence of the manipulations suggests that the engraving was a purposeful component of the cannibalistic practice, implying a complex ritualistic funerary behaviour that has never before been recognized for the Palaeolithic period.

## Introduction

Engraving is usually associated with the intellectual creations of *Homo sapiens* [1], although isolated finds of linear designs engraved on shells from Trinil in Java (ca. 0.5 million years [2]) and on a number of personal ornaments and decorated bone tools associated with Neanderthals [3, 4] suggest that such behaviour was not restricted to this species [5]. Mobiliary art perceptibly spread across Europe, from the Atlantic coast to the Urals, during the Aurignacian (ca. 40,000–34,000 BP), and reached its prime during the Magdalenian (ca.17,000–12,000 years BP [6–9], uncalibrated dates). Throughout this latter period we see the development of rich decorative forms, with many portable objects on bone, antler and ivory engraved with animal representations (e.g., [10–13]) or geometric designs (e.g., [14–17]). However, with the possible exception of an engraved human skull from the Magdalenian site of Isturitz (France [18]), which is still debated, the engraving of human bones is completely absent from the Palaeolithic archaeological record.

**Fig 1:**
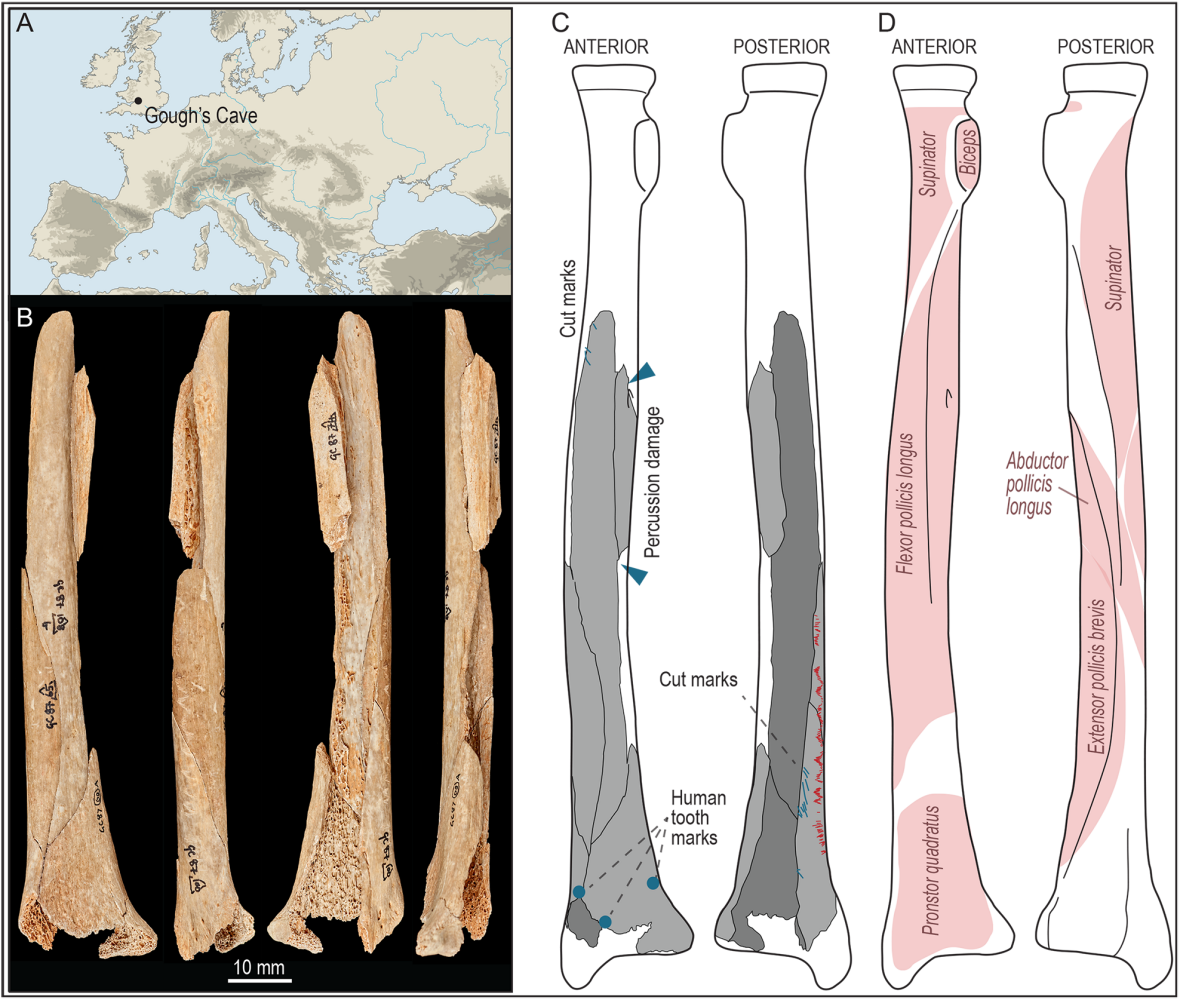
Site location and engraved human radius. (A) Location of Gough’s Cave (UK). (B) Photo of the engraved radius M54074 (anterior, medial, posterior and lateral sides). (C) Drawing of the preserved portion of the radius showing the location of the engraving marks (in red), human tooth marks (blue dots) and percussion damage (blue arrows). (D) Sketch of the location of muscles and muscle insertions on a human radius.

One of the most extensive Magdalenian human bone assemblages comes from Gough’s Cave, a sizeable limestone cave set in Cheddar Gorge (Somerset, UK). Analyses of the human remains have provided unequivocal evidence for nutritional cannibalism [19, 20] and the modification of human calvaria into skull-cups [21]. In this paper, we describe a right human radius (M54074), excavated in 1987 by a team led by Natural History Museum palaeontologists at the Magdalenian site of Gough’s Cave (Somerset, UK; Fig 1A). This bone has been humanly modified by cut marks, percussion damage and human tooth marks, as well as exhibiting a set of unusual zig-zagging incisions on the lateral side of the diaphysis. These were previously noticed by Jill Cook, who suggested possible engraving of the bone surface (pers. comm.), but this interpretation was criticized and ultimately dismissed by Andrews and Fernández-Jalvo [19, 22] who classified them as filleting marks created during butchery. Our new macro- and micro-morphometric analyses of the marks as well as comparisons with Magdalenian artefacts suggest that these modifications are in fact the result of intentional engraving. The association of an engraved human bone with a multi-stage cannibalistic practice suggests that the engraving was a purposeful component of the ritual. This is a unique example of complex cannibalistic funerary behaviour that has never been recognized before within a Palaeolithic context.

## Methods

Research on the human bones received ethical approval and consent at the Natural History Museum (London, UK). Modifications observed on the human remains were identified and classified as slicing cut marks (*sensu* [23]), percussion damage (*sensu* [24–27]) and human tooth marks [28–31]. Incisions made during engraving are similar to cut marks; however, the intentionality of their production can result in distinctive micro-morphometric characteristics [32]. To demonstrate that the incisions observed on the human radius (M54074) are the result of artistic engraving as opposed to butchery, we compared their micro-morphological characteristics to 322 filleting marks on human and non-human remains and to 119 engraving incisions observed on two artefacts (an engraved horse rib, BS27 3QF and an engraved hare tibia, BS27 3QF) from Gough’s Cave (S1 File). Artefacts, human and non-human remains were initially examined using a hand lens and binocular microscope. The modifications were further examined using an LEO1455VP scanning electron microscope (SEM), operated in variable pressure mode (chamber pressure 15 Pa). The topography of surface modifications was recorded using a Focus Variation Microscope (FVM), the Alicona InfiniteFocus optical surface measurement system. This system was used to produce three-dimensional (3D) micro-morphological models of the incisions (cut marks and engraving) according to the methodology described by Bello and co-authors [33, 34]. Micro-morphometric parameters for the engraving marks were assessed following the protocol of Bello et al. ([35], pages 2467–2469 and Fig 5 within). Cut marks and engraving incisions were measured at their midpoint. The profile measurements considered were: length of the incision (L), width of the incision at the surface (WIS), width at the bottom of the incision (WIB), opening angle of the cut (OA), depth (D) and angle of the tool inclination (ATI) during cutting [35].

## Results

### Archaeological context

Gough’s Cave (Long. 51.281869, Lat. -2.765523) is a large show-cave opening on the southern side of Cheddar Gorge, south-western England. The cave, discovered in the 1880s, was developed for tourism with extensive excavations only part of which were supervised by archaeologists. R. F. Parry later made better-recorded excavations between 1927 and 1931. More recently, excavations at Gough’s Cave were carried out by a team from the Natural History Museum between 1986 and 1992, investigating a small remnant of what turned out to be undisturbed sediment protected by a large fallen block near the entrance to the cave [36]. The results highlighted the exceptional richness of the deposits with notable finds of mobiliary art [37, 38], a dense cluster of refitting flint debitage, and butchered human and non-human bones. The human bones were deposited on the floor of the cave along with butchered large mammal remains and were exposed to limited chemical weathering and trampling. New ultrafiltrated radiocarbon determinations demonstrate that the cave was occupied by Magdalenian hunters for a very short span of time, possibly no more than two or three human generations, at the onset of Lateglacial Interstadial 1 (GI-1e, Bølling chronozone of the European record) at about 14, 7000 cal BP [39].

### Evidence for cannibalism

The Upper Palaeolithic human assemblage is characterized by scattered, highly fragmentary postcranial bones and relatively complete cranial vaults. We calculate a Minimum Number of six individuals: a child (aged 3.2 years), a young adolescent (approximately 12–14 years old), an older adolescent (approximately 14–16 years old), at least two adults and an older adult [20].

The human remains were modified regardless of the age of the individuals, and show clear signs of butchery. These have been detailed elsewhere [20, 21, 40], and therefore will only be summarised here. The frequency of cut-marked bones at Gough’s Cave exceeds 65%, and includes cuts made during disarticulation, scalping, and filleting of soft tissues. Cut marks were observed on persistent articulations (e.g., atlanto-occipital and lumbar articulations, on the neck of the femur and on the tibial tableau) as well as on labile articulations (e.g., on several bones of the hand and foot, on four cervical vertebrae and the scapulo-thoracic joint), suggesting that cutting occurred soon after the death of the individual. Micro-morphometric analyses of the cut marks on human and non-human remains at Gough’s Cave also suggest similar butchery patterns, with the metric values for cut marks on human remains intermediate between large and small animals (cf., [40]). About a third of the human bones (35%) exhibits percussion damage consistent with marrow and grease processing, and 87 postcranial fragments show evidence of human tooth marks, accounting for 42% of the bone fragments [20].

All modifications observed on the cranial fragments (cut mark frequency on the cranial fragments reaches 95.1%) are indicative of meticulous soft tissue removal to allow for controlled percussion and chipping of the broken edges in the manufacture of skull-cups [21]. It is unclear whether the organic tissue extracted from the skull was consumed, or if the cleaning of the skull was ritualistic and only associated with the manufacturing of a cup. It is therefore probable that cannibalism at Gough’s Cave took place as part of a mortuary ritual that combined the intensive processing of entire corpses to extract edible tissues and the modification of skulls to produce skull-cups [20].

### Mobiliary art

**Fig 2:**
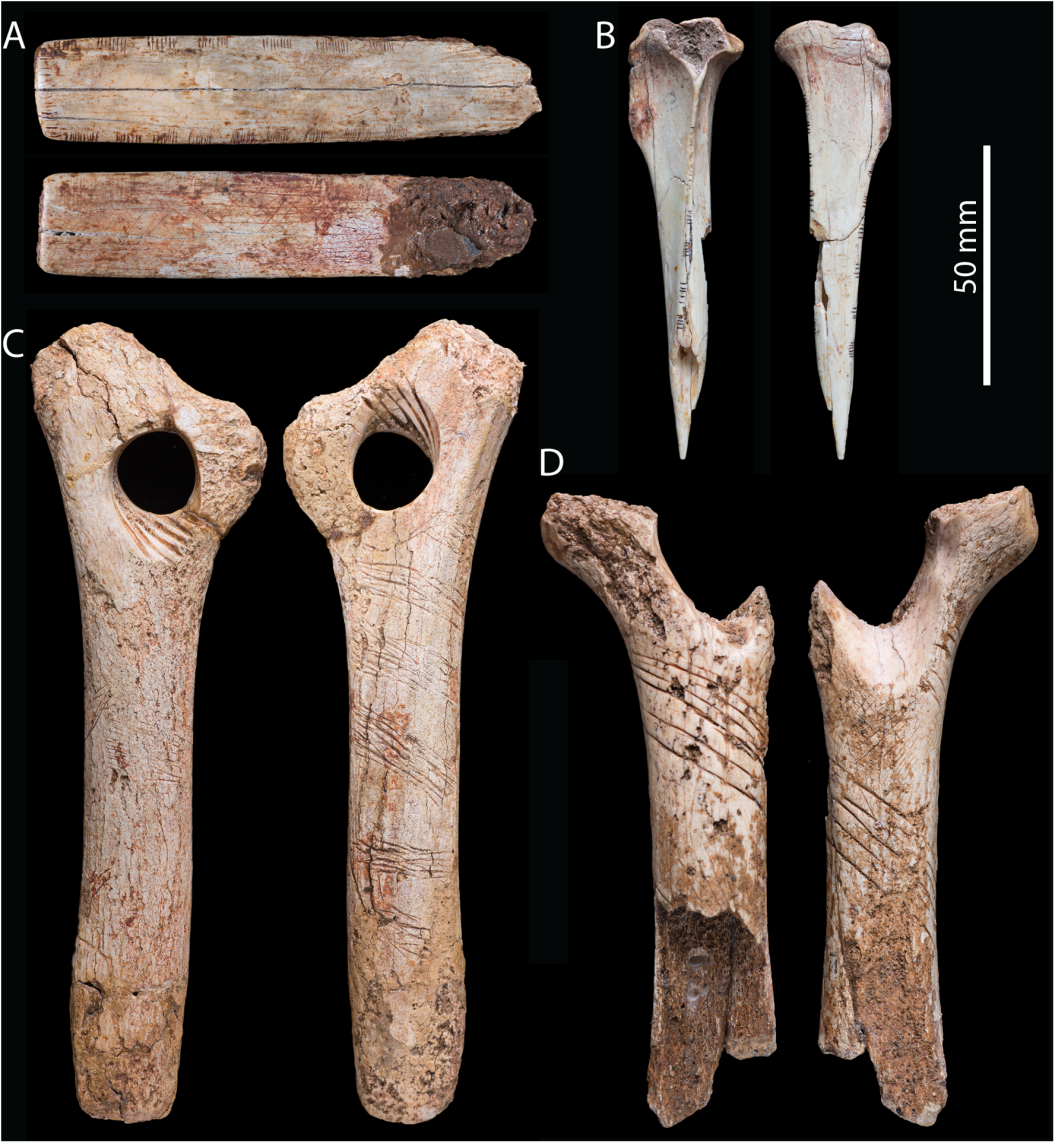
Engraved artefacts from Gough’s Cave. (A) engraved horse rib (BS27 3QF), ventral and dorsal views; (B) engraved hare (*Lepus timidus*) tibia (BS27 3QF), ventral and lateral views; (C) ‘*bâton percé*’ made from reindeer (*Rangifer tarandus*) antler (PAE 7782), front and rear views; (D) fragment of ‘*bâton percé*’ made from reindeer antler (PAE 7783), front and rear views. Engraving marks on artefacts A and B were measured for micro-morphometric comparisons with engravings on the human bone.

Mobiliary art at Gough’s Cave includes three ‘*bâton percé*’ made from reindeer antlers, worked and engraved fragments of hare tibiae and the rib shaft of a horse, amber pebbles and minute fragments of ivory with groups of incisions [37, 38, 41, 42] (Fig 2). The manufacture of the tools and the engraved designs on the bone and ivory artefacts at Gough’s Cave have close parallels to those found at other European Magdalenian sites (e.g. [16, 43–46]). All the bone artefacts, but in particular the two ‘*bâtons percé’*, are considerably weathered and smoothed, suggesting they had been carried and handled for a considerable period of time before being discarded. Overall, the mobiliary art at Gough’s Cave suggests that the carvers were competent and experienced in working and engraving different raw materials, with some traces indicating advance technical expertise, consistent control of the tool and gestural precision [37, 38].

### The engraved human radius

The re-fitting of five diaphysial fragments and one fragment of distal epiphysis forms an almost complete human right radius, M54074 (Fig 1B). Because of its overall dimensions (maximal length = 154 mm and maximal width = 33mm), and by comparison with other radii at the site, it is likely that M54074 belonged to a gracile adult individual. Apart from slight signs of weathering, the bone surface is generally sound.

The human radius has been modified by clusters of transverse cut marks on the lateral surface close to the proximal portion of the diaphysis, and on its distal posterior surface produced during filleting of the *supinator*, *flexor pollicis longus* and *extensor pollicis brevis* muscles (Fig 1D). The radius has been heavily fragmented owing to marrow processing, which has resulted in lengthwise fractures. The longitudinal breakage and associated percussion pits and cracks are mainly located on the diaphysis (Fig 1B and 1C). This breakage pattern is consistent with bone cracking resulting from marrow extraction as observed on other human long bones at Gough’s Cave [20]. Human tooth marks (scooping-out and saw-toothed edges) have also been observed on two fragments of its distal shaft. One of the fragments (GC87 60A) shows a crescent-shaped pit. This set of bone modifications is a clear indication that the radius, similarly to other human remains at Gough’s Cave, was damaged during cannibalism [40].

**Fig 3:**
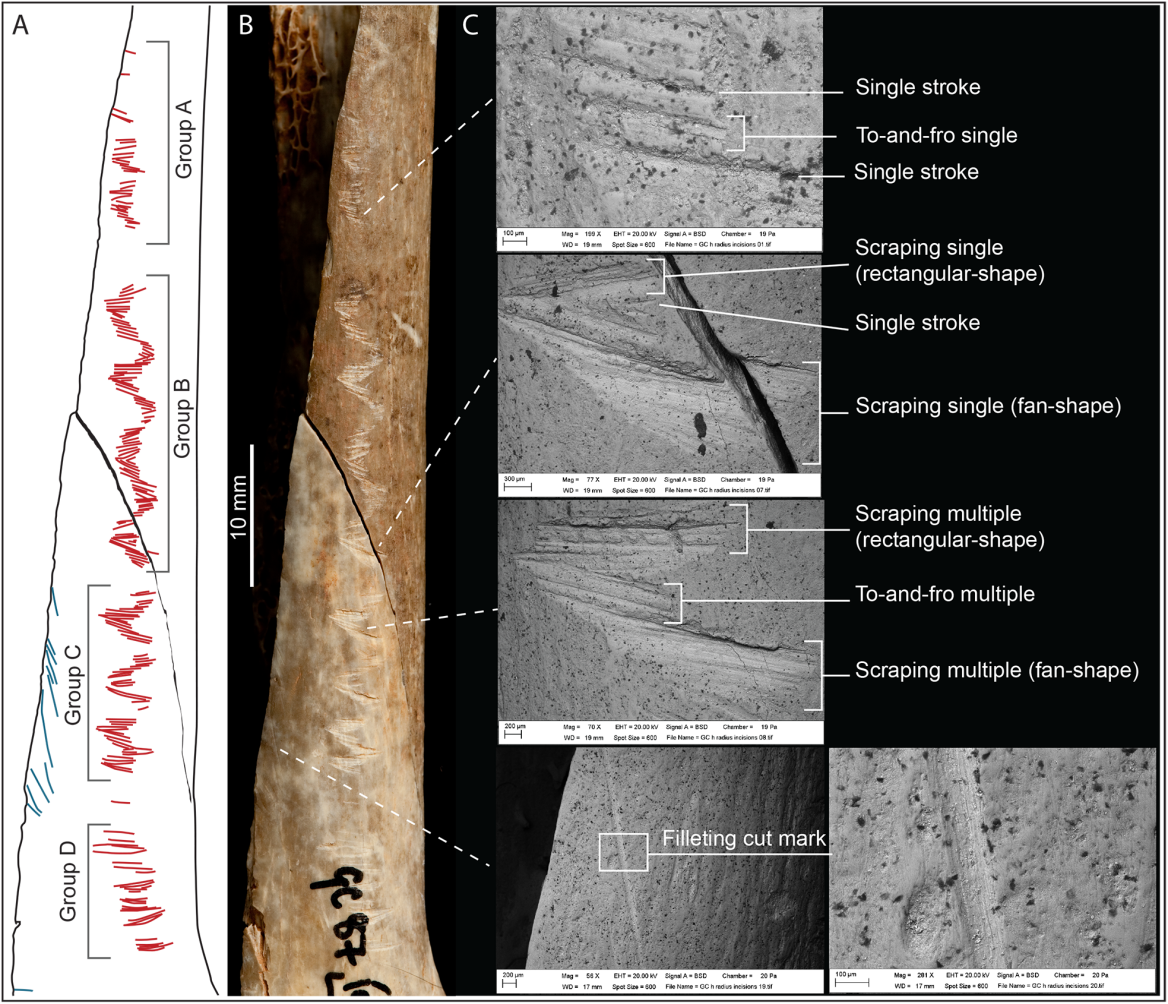
Details of the incisions on the engraved human bone. (A-B) Photo and drawing of the engraving on the human radius M54074, with division into the four groups (A-D). (C) Scanning electron microscope images detailing the different types of incisions.

A set of zig-zagging incisions can be observed on the lateral side of the diaphysis. These marks are located on a region without muscle attachments and therefore cannot be attributed to filleting (Fig 1B–1D). Their micro-morphological and micro-morphometric characteristics (described in detail below) also suggest these modifications are different from butchery marks. There are no scrape marks in the area of the bone where these incisions are found, so there does not seem to have been any form of preparation of the bone surface prior to incision. The geometric zig-zag design of the entire cluster of marks is about 63 mm long. The cluster of incisions follows the longitudinal fracture, although, toward the proximal end of the radius, it is cut short by breakage, suggesting the incisions were performed before bone cracking occurred. Four clusters of regularly spaced incisions (Fig 3A–3D) can be distinguished along the full length of the zig-zag design, and the distance between each cluster and their respective width and length are fairly homogenous (Table 1).

**Table 1:** Number of cuts, length and average width of the four groups of incisions forming the engraving and distances between each cluster.

Group	No. cuts	Length of the cluster (mm)	Average width of the cluster (mm)
**A**	20	12.1	3.5
**Distance between A-B**		5.6	
**B**	40	19.4	4.3
**Distance between B-C**		1.6	
**C**	17	12.7	5.4
**Distance between C-D**	1	4.0	
**D**	9	8.9	4.3

Each cluster of marks is a combination of (a) ‘single-stroke’ incisions, (b) ‘to-and-fro’ incisions often with recognizable multiple internal sub-incisions (*sensu* [35]) produced by a sawing action, and (c) ‘scraping or chiselling’ incisions, often with recognizable multiple internal sub-incisions produced by pressure scraping (Fig 3B and 3C). Within each cluster of incisions (A-D), the succession and combination of single-stroke, to-and-fro, and scraping incisions appear to be ordered, with single incisions opening and ending each cluster, and larger scraping incisions more often located in the middle of each cluster (for details of each incision refer to S1 File and S2 File). Incisions in Group D, however, appear less well organized into a geometric zig-zag design with a higher number of isolated single incisions (either single-stroke, or to-and-fro and scraping single incisions). Single-stroke incisions are very similar to slicing/filleting cut marks. However, what is remarkable for the Gough’s Cave radius is that all single-stroke incisions present consistent micro-morphometric parameters (Table 2). The to-and-fro and scraping incisions may appear similar; nevertheless it is possible to distinguish them at a microscopic level because they are the result of opposing actions. In the case of the Gough’s Cave radius, to-and-fro incisions are produced by a tool moving (slicing) across the surface, transversally to the long axis of the diaphysis (Fig 3). Their micro-morphometric profiles are quite similar to those of filleting cut marks, where single incisions overlap to produce larger incisions [14, 35]. As for the single-stroke incisions, the lengths of each to-and-fro incision are very consistent (Table 2). The scraping incisions are produced by pressure scraping, in the case of the Gough’s Cave radius along the long axis of the diaphysis (Fig 3), without displacing the tool (*sensu* [14]). On the surface of the bone, scraping incisions assume either a rectangular shape, resulting from the pressure being exerted along the entire cutting edge of the tool, or a fan shape, resulting from unequal pressure produced as the tool pivoted around one of the corners of its cutting edge (Fig 3C). The micro-morphological characteristics of these incisions suggest that the chiselling action may have been unintentionally interrupted in some cases, possibly because the tool went too deep into the bone surface and was subsequently lifted before continuing the scraping of the bone surface. In this case, consecutive sub-incisions are visible within a scraping incision (Fig 3C).

**Table 2:** Average length and average measurements of the profile parameters (taken at the incision’s midpoint) of incisions (according to their types) and cut marks on human and non-human bones from Gough’s Cave.

	No. cuts		L (mm)	WIS (μm)	WIB (μm)	OA (°)	D (μm)	ATI (°)
**Single-stroke** **incision**	33	Mean	1.64	230.64	75.27	151.61	20.47	90.51
		Max	2.89	366.58	113.92	165.35	41.86	102.40
		Min	1.04	185.40	40.72	134.32	17.02	75.59
		SD	0.52	57.02	21.48	8.34	7.31	6.82
**To-and-fro incision (single)**	13	Mean	1.82	338.52	101.62	154.56	33.76	88.79
		Max	2.93	465.05	176.25	166.43	60.64	92.57
		Min	1.02	239.96	53.21	139.91	13.34	83.37
		SD	0.56	63.15	32.14	7.72	15.17	2.77
**To-and-fro incision (multiple)**	32	Mean	1.75	364.42	65.30	148.49	22.53	87.92
		Max	4.14	570.91	209.72	168.75	55.33	100.45
		Min	0.69	236.62	24.28	112.82	8.63	68.53
		SD	0.70	89.56	33.18	13.41	12.76	6.39
**Scraping incision (single)**	13	Mean	2.32	721.58	226.49	139.22	82.19	81.52
		Max	3.37	1227.7	499.55	162.95	132.75	93.66
		Min	1.37	415.72	78.79	86.72	25.51	59.37
		SD	0.56	270.71	104.93	22.49	39.20	9.95
**Scraping incision (multiple)**	9	Mean	1.87	665.79	94.98	153.29	44.38	87.03
		Max	2.85	954.23	252.33	170.23	109.94	94.34
		Min	1.25	502.03	20.17	128.64	8.43	70.14
		SD	0.44	153.33	63.05	11.75	27.35	6.13
**Engraving on artefacts**	119	Mean	2.59	317.76	93.41	147.90	34.29	88.92
		Max	4.13	672.11	312.83	169.68	137.77	110.97
		Min	1.04	68.49	25.34	101.49	9.30	41.12
		SD	0.78	108.22	43.82	15.94	23.98	6.87
**Filleting marks on human radii**	37	Mean		229.68	97.03	140.85	22.81	88.22
		Max		458.49	322.23	169.25	45.14	102.25
		Min		80.22	19.67	110.18	8.74	74.10
		SD		94.59	60.51	13.39	9.09	7.52
**Filleting marks on human bones**	207	Mean		259.65	91.34	138.40	32.74	88.26
		Max		734.84	402.69	169.25	103.86	128.05
		Min		71.21	19.28	56.77	5.74	60.48
		SD		129.58	58.71	18.28	22.06	8.31
**Filleting marks on large animal bones**	44	Mean		305.79	121.90	123.51	56.65	86.36
		Max		619.02	375.38	165.43	119.59	99.77
		Min		102.25	39.88	92.04	14.44	63.57
		SD		129.23	74.98	20.92	27.09	8.22
**Filleting marks on small animal bones**	71	Mean		250.59	73.87	139.82	32.83	89.75
		Max		755.04	152.19	168.65	85.92	112.16
		Min		65.86	18.19	94.93	7.05	69.26
		SD		135.00	39.34	15.78	18.18	7.19

Length of the incision (L), width of the incision at the surface (WIS), width at the bottom of the incision (WIB), opening angle of the cut (OA), depth (D) and angle of the tool inclination (ATI).

**Fig 4:**
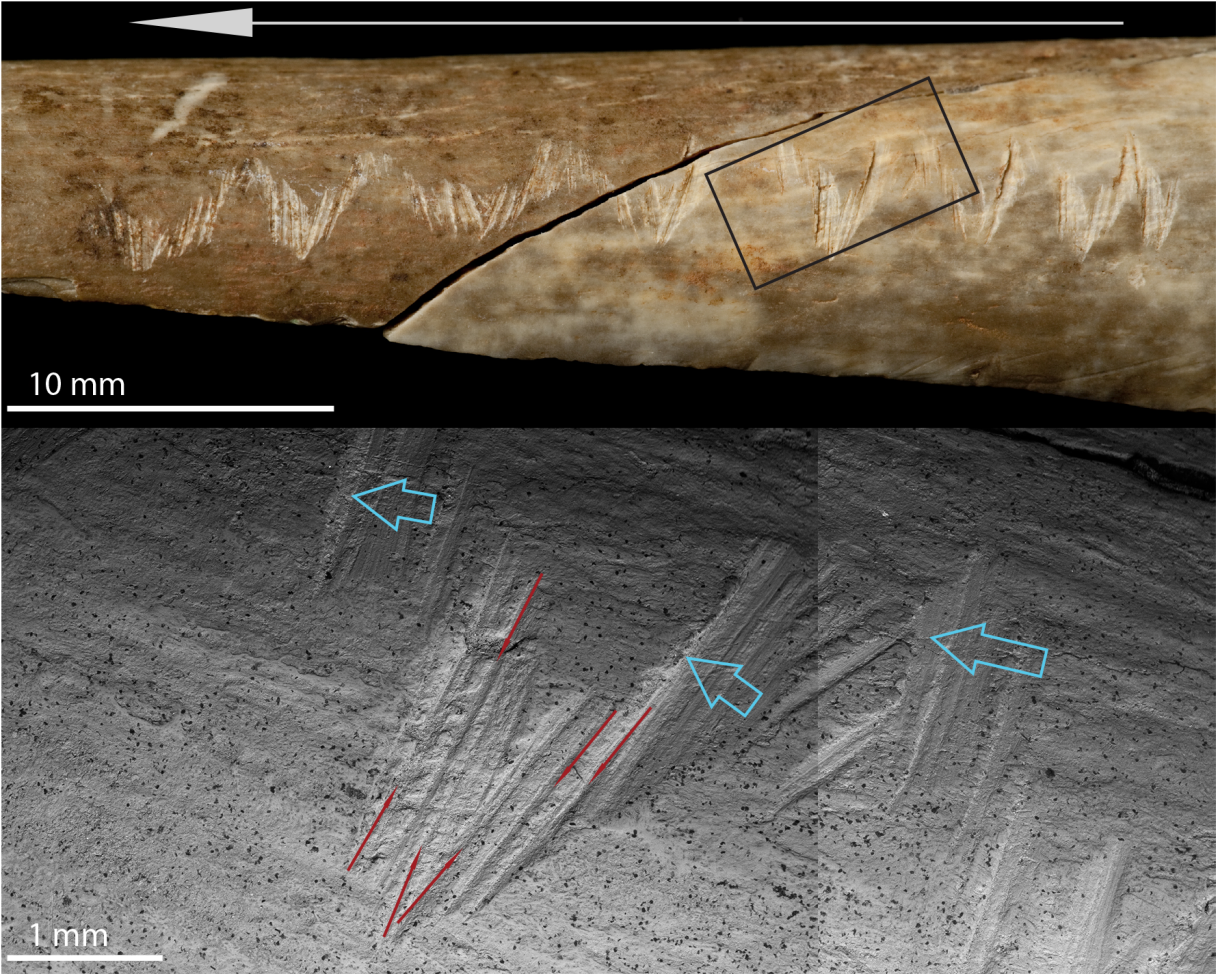
Details of the engraving design on the human radius. Photo and scanning electron microscope composite image illustrating the directionality of single incisions and the overall directionality kept while producing the entire engraving. Red arrows indicate to-and-fro sawing incisions, blue arrows scraping incisions.

The entire engraving on the human radius is composed of 87 incisions: 33 single-stroke incisions, 32 to-and-fro sawing incisions (13 of which appear as a large single-incision due to the complete overlapping of the sub-incisions, and 19 as incisions with multiple internal sub-incisions) and 22 scraping incisions (13 of which were due to homogeneous pressure along the scraped surface, resulting in a single large incision, and 9 were due to variable pressure, resulting in multiple recognizable internal sub-incisions). The micro-morphometric details of each incision are presented in S1 File and S2 File. Overall, the length of each incision is constant along the entire engraving, with minimal standard deviation, regardless of the types of incision (single-stroke, to-and-fro, or scraping incision; SD = 0.6 mm; Table 2). Similarly, the micro-morphological profile and the opening angle for each incision forming the engraving are also quite consistent regardless of the type of incision (SD = 13.7°). This suggests that the same tool, likely a unilaterally retouched blade (refer to S2 File for details) was used to produce the entire engraving. Single-stroke incisions are on average narrower and shallower (Table 2) compared to to-and-fro (width at the incision at the surface (WIS): p < 0.001; depth (D): p = 0.036) and scraping incisions (WIS: p < 0.001; D: p < 0.001). Single-stroke incisions were predominantly produced by holding the tool fairly perpendicular to the bone’s surface, more similar to cutting/disarticulation marks than filleting marks (values of the angle of the tool impact, ATI; [40, 47]). In the case of to-and-fro and scraping incisions, the tool was generally held at an angle less than 90° relative to the bone’s surface (Table 2, S1 File). These results suggest that the entire engraving was produced during one single event, with the engraver holding the tool at a consistent angle through the gesture. The combination of overlapping incisions, microscopic characteristics (e.g., hertzian cones) and ATI values indicates the engraving was produced starting from the distal end of the diaphysis of the radius toward its proximal epiphysis (Fig 4). It is not, however, possible to determine whether the engraver was right- or left-handed due to the different positions in which the radius could have been handled (cf. [48]). The final result can in fact be obtained holding the radius with the proximal epiphysis toward the engraver and working towards oneself (this is possible either for a right- or a left-handed person). The same effect could also be obtained by working laterally from right to left with the medial side of the radius toward oneself, for a right-handed person, or working laterally from left to right with the lateral side of the radius toward oneself for a left-handed person.

**Fig 5:**
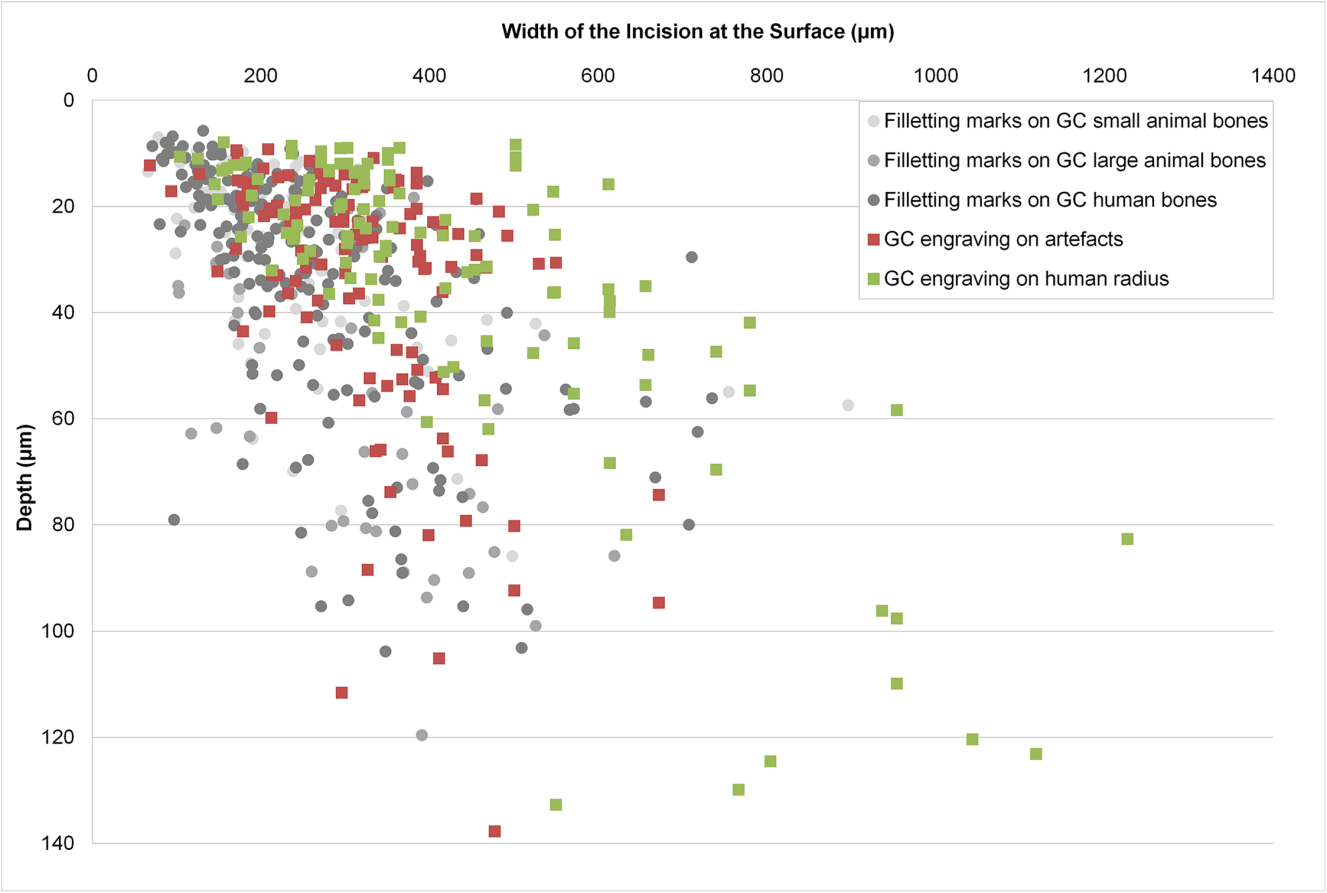
Micro-morphometric values of incisions measured on human and non-human bones from Gough’s Cave. Graph of the micro-morphometric values of the width plotted against the depth of cut marks on large/small animals and human bones from Gough’s Cave (GC), and of incisions on engraved artefacts and the engraved human radius (M54074) from Gough’s Cave.

Comparisons with engraving on other artefacts at Gough’s Cave show several similarities in terms of the overall pattern of the design and consistency in the length of each incision. The micro-morphometric characteristics of engraving on the horse rib and the hare tibia fall between the single-stroke and to-and-fro incisions observed on the engraved radius (Table 2). This result was expected, as engravings on the artefacts from Gough’s Cave were more often made using a single-stroke technique and in 7 cases (out of 112 incisions analysed) using a to-and-fro action (S1 File). No scraping incisions were recognized among the engraved artefacts, making the engraved human radius unique in the use of this technique. The absence of scraping marks and the few examples of multiple to-and-fro incisions on the engraved artefacts explain the smaller width of their incisions compared to the ones on the human radius (p < 0.001; Fig 5). Analyses of the micro-morphometric characteristics of filleting marks on human (207 cut marks measured) and non-human bones (115 cut marks measured) from Gough’s Cave highlight the dissimilarities between filleting marks and engraving (S1 File). Filleting marks do not present consistent lengths. Although filleting marks and single-stroke incisions share similar micro-morphological characteristics (Fig 3C), the differences between the micro-morphometric profile parameters of filleting marks and to-and-fro and scraping incisions on the human radius are obvious (Table 2). Also in this case, the absence of scraping marks and to-and-fro incisions results in significant differences in the width of filleting marks compared to the width of the incisions on the human radius (p < 0.001). These results strongly suggest the use of different techniques of cutting (normally single-stroke slicing marks) versus engraving (use of multiple techniques of marking the bones).

## Discussion and conclusions

The use of human bones as raw material to produce utilitarian or symbolic tools in Palaeolithic Europe is rare. The earliest known example has been described by Verna and d’Errico [49] for the Mousterian site of La Quina (France) where three human cranial fragments have been interpreted as retouchers (knapping tools made from organic materials used in the manufacture of stone tools). However, the authors suggest that the use of Neanderthal bones was accidental and not a choice. Neanderthal skeletal elements used as retouchers have also been described from the sites of Krapina (Croatia [50]), Les Pradelles (France [49, 51]) and Troisième caverne of Goyet (Belgium [52]). A few European Upper Palaeolithic sites have yielded human teeth interpreted as personal ornaments [53, 54] and human calvaria fashioned to make skull-cups [18, 21, 55–57]. A unique example of a Chalcolithic (3^rd^ millennium BC) human femur has recently been described as a burin, possibly to drill animal hide [58]. None of these human objects appears to have been decorated (but see [18]). Only two European Mesolithic (~ 6000 cal BP) examples of engraved human remains have been recognized so far: a radius engraved with a series of notches from the site of Lepenski Vir (Serbia [32]), and a child’s rib incised with parallel lines from the site of Téviec (France [43, 59]). Perforated human teeth are likewise rare, so far reported from only three European Mesolithic sites [60]. Decorated human bones have been described from Neolithic contexts for the sites of Adlestrop [61] and Hambledon Hill [62] in Britain. Worked and engraved human bones and teeth are a more common feature in Holocene sites in North and Meso-America [63–67].

There is no doubt that the zig-zagging incisions on the diaphysis of the human radius from Gough’s Cave are engraving marks, produced with no utilitarian purpose apart from an artistic representation. Similar to other engraved bone artefacts from Gough’s Cave, the incisions are very consistent in their lengths, overall pattern, and technique of production (e.g., single stroke and to-and-fro incisions). Unlike filleting marks, which are generally produced in a single-stroke slicing motion, the engraving is produced by a combination of single-stroke, to-and-fro, and scraping incisions. This combination results in quantifiable differences between the macro- and micro-morphometric characteristics of the engraved incisions and filleting marks on human and non-human bones from Gough’s Cave. The more unusual engraving marks observed on the human radius are those produced by pressure during a scraping action. These scraping incisions have not been recognized on any other engraved or filleted animal bone at the site. High values of width and depth of this type of engraving are only comparable with measurements of width and depth of filleting marks on large mammals at Gough’s Cave. Previous studies have suggested a pattern of wider and deeper cut marks associated with the higher strength necessary to fillet larger muscles [40, 68]. It is therefore plausible that the engraving by scraping was produced using greater pressure than the production of the engraving by slicing. This extra strength required is unlikely to be due to a larger amount of meat, but rather the intentionality of leaving visible marks on the bone. However, what is striking is the consistency in the values of the opening angle for all incisions (engraving and filleting marks) measured on bones at Gough’s Cave, which suggests that similar tools were used to butcher human and non-human carcasses, and for engraving bones. The even closer similarity of the micro-morphometric parameters of filleting marks on the human bones and the engraving itself (Table 2, Fig 5) may also indicate that the filleting of human bodies during cannibalism and the engraving of human bones were intricately related, probably made using the same tools, by the same butcher/engraver and in succession.

**Fig 6:**
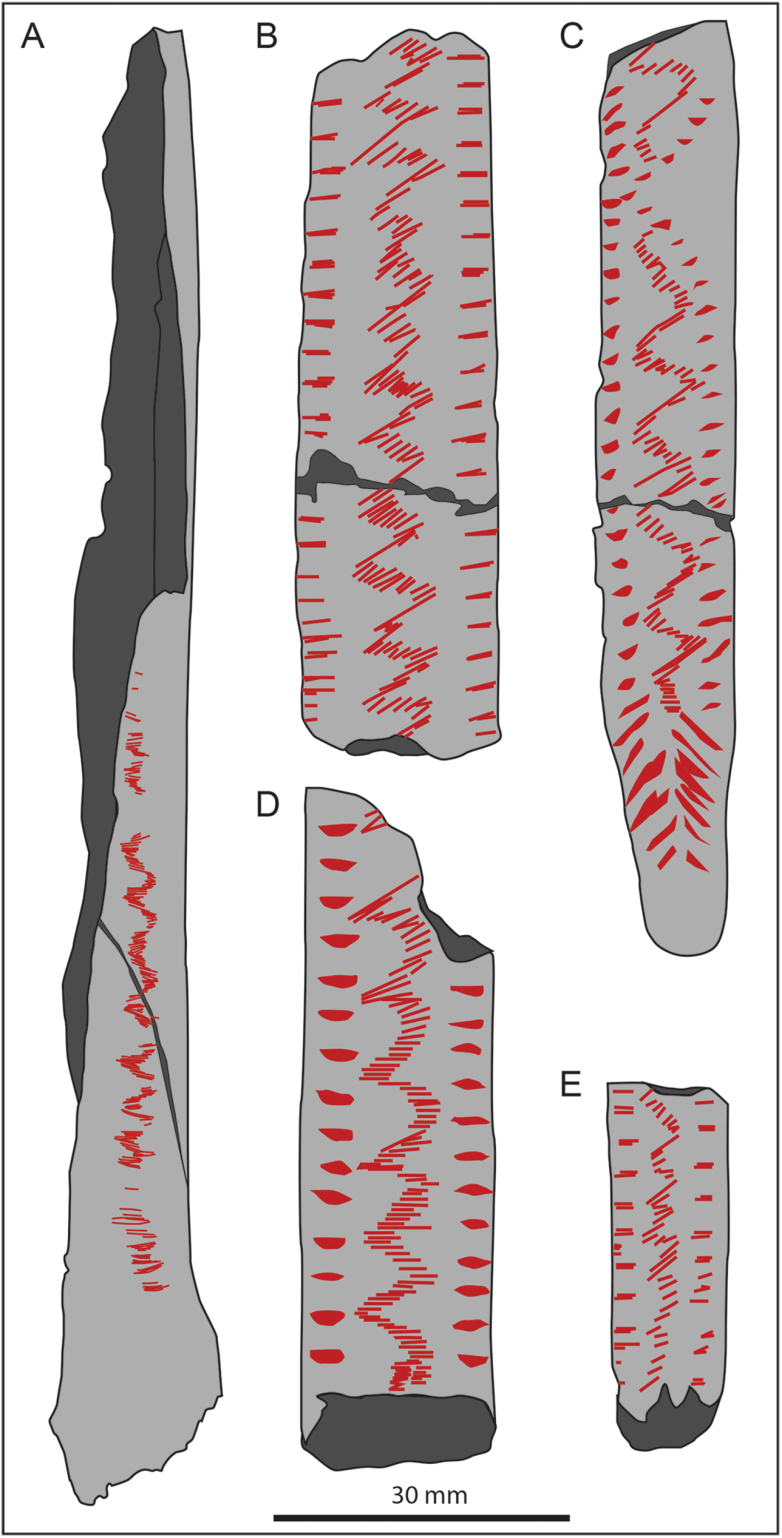
Drawings of Magdalenian engraved artefacts. (A) Gough’s Cave radius (M54074); (B) Engraved lissoir (MAN 48716) from Brassempouy (France); (C) Engraved lissoir (Duru 35) from Duruthy (France); and (D-E) Engraved lissoirs (Ist 288 and Ist 284) from Isturitz (France). Drawing of the French artefacts were obtained from photos, courtesy of Claire Lucas [16]. All drawings were drafted by S.M.B.

Although the scraping incisions appear to be the result of an unusual engraving technique, the zig-zagging pattern itself is not unique for this period. Repetitive geometric designs, sometimes called ‘schematic’ or non-figurative art, are very common in Palaeolithic art. Frequent forms include notches, zig-zagging lines, wave and chevron patterns [15, 17, 43, 45, 46, 69, 70, 71]. Widespread during the Magdalenian is the engraving of zig-zagging (or ‘criss-crossing’ according to different authors) motifs formed by successive parallel incisions cutting the main axis of the artefacts perpendicularly [46, 72–74]. This type of representation seems to be particularly recurrent for the Middle Magdalenian (~15,000–14,000 BP) in south-west France. Examples that present decoration remarkably similar to the one on the human radius from Gough’s Cave have been observed at the sites of Brassempouy, Duruthy and Isturitz [16, 75–78] (Fig 6). In all these cases, the engraving was on a flat bone surface, more often from a rib of a large mammal, modified to produce lissoirs (bone tools used to prepare and smooth hides). The engraving of these artefacts was generally preceded by the manufacture of the bone tool (lissoir), and scraping of the bone surface from the periosteum. Overall, these modifications are indicative of a more complex *chaîne opératoire* in the manufacture and engraving of an object that was probably frequently used, curated and carried around for some time [74, 79].

The engraved motif observed on the human radius from Gough’s Cave (for which human bones have been directly dated at 14,700 cal BP [39]) comfortably fits within a Middle Magdalenian pattern of engraving (Fig 6). What is exceptional in this case, however, is the choice of raw material (human bone), and the context in which it was produced. The engraved bone was part of a cannibalistic ritual and the individual to whom it belonged was certainly disarticulated, skinned and its muscles filleted. It is possible that the skull was used to produce one of the skull-cups (although only DNA analysis, currently in progress, could associate this radius with one of the skull-cups found on site [21]) while the long bones were chewed and broken to extract marrow. The radius itself presents evidence for disarticulation, filleting and chewing marks. The breakage of the bone to extract marrow, however, cut across the engraving. It is therefore likely that the bone was engraved after it was cleaned of its muscle and tendons (but not of its periosteum by scraping), but before breakage occurred. The engraving itself was produced by a single individual using one tool and during only one event. The bone was probably placed flat, and the engraving produced from the distal end of the diaphysis toward the proximal epiphysis. The sequence of the manipulations strongly suggests that the engraving was an intrinsic part of the multi-stage cannibalistic ritual and, as such, the marks must have held a symbolic connotation. We can only speculate about the context in which this happened. The engraving of objects and utilitarian tools has been linked to ways of remembering events, places or circumstances, a sort of ‘written memory’ and ‘symbolic glue’ that held together complex social groups [80, 81]. The engraving of this bone may have been a sort of ‘story-tale’ more directly related to the deceased than the surrounding landscape, perhaps indicative of the individual, events from their life, the way they died, or the cannibalistic ritual itself. Unlike the other artefacts present on site, the engraving was not made to last over time and the radius was not made into an object that the group would have carried around. It was quickly engraved, with minimal preparation (i.e., no scraping of the bone surface), possibly not following more standard procedures (e.g., the presence of ‘scraping marks’ has never been observed on any other Magdalenian engraved artefacts) or sophisticated techniques (cf. [80, 82]) compared to the engraved bone and antler artefacts at Gough’s Cave. It was then broken and discarded. The sequence of the manipulations can only imply that the engraving was a purposeful component of the cannibalistic ritual at the site, with the act of engraving itself as significant as the finished motif, suggesting a complex funerary cannibalistic behaviour that has never been recognized before for the Palaeolithic period.

## Supporting information

S1 File **Tables 1–4, Type of incision, length and micro-morphometric profile values, taken at the incision’s midpoint, of each engraved incision on the human radius (M54074), two artefacts engraved bones (BS27 3QF) and filleting marks on human and non-human remains from Gough’s Cave.** Length of the incision (L), width of the incision at the surface (WIS), width at the bottom of the incision (WIB), opening angle of the cut (OA), depth (D) and angle of the tool inclination (ATI)
(DOCX)

S2 File 3-Dimensional Alicona image, Alicona profile (with and without measurements) and description of each engraving mark on the human radius (M54074). (DOCX)
