# Supplementary material for: An Upper Palaeolithic engraved human bone associated with ritualistic cannibalism
Source: PLoS One. 2017 Aug 9;12(8):e0182127. doi: 10.1371/journal.pone.0182127 (PMC5549908; doi:10.1371/journal.pone.0182127)
Supplement: S1 File — Tables 1–4, Type of incision, length and micro-morphometric profile values, taken at the incision’s midpoint, of each engraved incision on the human radius (M54074), two artefacts engraved bones (BS27 3QF) and filleting marks on human and non-human remains from Gough’s Cave. Length of the incision (L), width of the incision at the surface (WIS), width at the bottom of the incision (WIB), opening angle of the cut (OA), depth (D) and angle of the tool inclination (ATI) (DOCX) [file pone.0182127.s001.docx]

**Supplementary Information 1**

**Tables 1-4: Type of incision, length and micro-morphometric profile values, taken at the incision’s midpoint, of each engraved incision on the human radius (M54074), two artefacts engraved bones (BS27 3QF) and filleting marks on human and non-human remains from Gough’s Cave.** Length of the incision (L), width of the incision at the surface (WIS), width at the bottom of the incision (WIB), opening angle of the cut (OA), depth (D) and angle of the tool inclination (ATI)

**Table 1:** Type of incision, cluster, length and micro-morphometric profile values, taken at the incision’s midpoint, of each engraved incision on the human radius (M54074) from Gough’s Cave. Length of the incision (L), width of the incision at the surface (WIS), width at the bottom of the incision (WIB), opening angle of the cut (OA), depth (D) and angle of the tool inclination (ATI).

| **Type of incision** | **Cluster** | **Incision** | **Sub-incisions** | **L (mm)** | **WIS (µm)** | **WIS (for multiple incisions, µm)** | **WIB (µm)** | **OA (˚)** | **D (µm)** | **ATI (˚)** |
| --- | --- | --- | --- | --- | --- | --- | --- | --- | --- | --- |
| Single-stroke | A | 1 |  | 1.2 | 322.45 |  | 156.98 | 163.51 | 13.7 | 86.35 |
| To-and-Fro double | A | 2 | A | 0.94 |  | 271.53 | 61.04 | 154.24 | 9.64 | 82.16 |
| To-and-Fro double | A | 2 | B | 0.94 |  | 271.53 | 84.01 | 168.06 | 11.72 | 89.94 |
| To-and-Fro single | A | 3 |  | 1.59 | 317.24 |  | 108.25 | 161.17 | 23.18 | 89.70 |
| To-and-Fro double | A | 4 | A | 1.52 |  | 236.62 | 41.89 | 154.80 | 10.07 | 81.29 |
|  | A |  | B | 1.42 |  | 236.62 | 61.45 | 162.82 | 8.63 | 85.92 |
| Single-stroke | A | 5 |  | 2.21 | 182.38 |  | 53.03 | 149.11 | 11.79 | 100.79 |
| To-and-Fro double | A | 6 | A | 1.91 |  | 352.95 | 70.24 | 154.39 | 14.15 | 95.70 |
|  | A |  | B | 2.45 |  | 352.95 | 83.31 | 149.46 | 15.11 | 90.66 |
| Single-stroke | A | 7 |  | 1.27 | 196.28 |  | 38.48 | 160.78 | 14.86 | 86.51 |
| Scraping double | A | 8 | A | 1.68 |  | 548.75 | 252.33 | 149.96 | 36.15 | 81.11 |
|  | A |  | B | 1.55 |  | 548.75 | 100.58 | 164.2 | 25.38 | 89.09 |
| To-and-Fro double | A | 9 | A | 0.82 |  | 280.83 | 86.45 | 156.63 | 13.43 | 85.29 |
|  | A |  | B | 1.06 |  | 280.83 | 62.49 | 165.85 | 13.17 | 90.19 |
| Single-stroke | A | 10 |  | 1.77 | 168.31 |  | 79.67 | 155.18 | 12.24 | 86.42 |
| Single-stroke | A | 11 |  | 1.59 | 104.21 |  | 53.89 | 140.65 | 10.67 | 79.78 |
| To-and-Fro single | A | 12 |  | 1.02 | 296.09 |  | 53.21 | 165.26 | 19.6 | 89.50 |
| To-and-Fro single | A | 13 |  | 1.47 | 356.03 |  | 121.79 | 158.56 | 23.93 | 84.30 |
| Single-stroke | A | 14 |  | 1.68 | 240.75 |  | 88.34 | 156.55 | 18.91 | 87.38 |
| To-and-Fro double | A | 15 | A | 1.54 |  | 294.25 | 102.57 | 162.73 | 11.99 | 90.89 |
|  | A |  | B | 1.56 |  | 294.25 | 48.31 | 166.39 | 9.04 | 93.69 |
| Scraping single | A | 16 |  | 1.37 | 444.98 |  | 212.7 | 162.95 | 32.44 | 88.26 |
| Single-stroke | A | 17 |  | 1.35 | 145.32 |  | 64.16 | 126.00 | 15.89 | 104.66 |
| Single-stroke | A | 18 |  | 0.91 | 125.27 |  | 40.24 | 153.04 | 11.08 | 95.75 |
| To-and-Fro single | A | 19 |  | 1.62 | 239.96 |  | 60.76 | 147.47 | 25.84 | 83.37 |
| Single-stroke | A | 20 |  | 1.14 | 155.99 |  | 50.70 | 165.01 | 7.94 | 91.69 |
| Single-stroke | B | 21 |  | 1.25 | 326.64 |  | 157.93 | 158.14 | 11.95 | 83.65 |
| Single-stroke | B | 22 |  | 1.22 | 257.52 |  | 85.95 | 164.12 | 15.08 | 89.68 |
| To-and-Fro double | B | 23 | A | 1.36 |  | 364.29 | 52.22 | 154.58 | 9.03 | 86.02 |
|  | B |  | B | 1.98 |  | 364.29 | 78.41 | 153.83 | 17.6 | 83.49 |
| Scraping double | B | 24 | A | 1.80 |  | 612.08 | 180.45 | 149.03 | 35.63 | 90.89 |
|  | B |  | B | 1.63 |  | 612.08 | 89.85 | 167.26 | 15.89 | 89.75 |
| Scraping single | B | 25 |  | 1.80 | 415.72 |  | 94.54 | 161.15 | 25.51 | 85.23 |
| Single-stroke | B | 26 |  | 0.99 | 177.22 |  | 72.77 | 161.65 | 12.22 | 94.03 |
| To-and-Fro double | B | 27 | A | 0.69 |  | 302.6 | 73.05 | 154.69 | 11.94 | 95.85 |
|  | B |  | B | 1.30 |  | 302.6 | 98.87 | 159.21 | 9.00 | 94.28 |
| Single-stroke | B | 28 |  | 1.08 | 154.53 |  | 57.02 | 156.52 | 13.26 | 89.62 |
| Single-stroke | B | 29 |  | 1.43 | 160.02 |  | 44.57 | 161.28 | 12.82 | 90.06 |
| Single-stroke | B | 30 |  | 1.58 | 149.03 |  | 56.77 | 140.65 | 18.73 | 97.57 |
| To-and-Fro double | B | 31 | A | 1.85 |  | 348.33 | 34.98 | 129.26 | 28.43 | 91.92 |
|  | B |  | B | 1.89 |  | 348.33 | 81.73 | 132.62 | 27.46 | 83.38 |
| To-and-Fro single | B | 32 |  | 1.85 | 303.32 |  | 106.03 | 157.18 | 26.12 | 90.99 |
| Single-stroke | B | 33 |  | 2.47 | 176.34 |  | 80.91 | 133.46 | 25.75 | 92.45 |
| To-and-Fro double | B | 34 | A | 1.21 |  | 311.31 | 56.69 | 150.17 | 16.77 | 86.25 |
|  | B |  | B | 1.25 |  | 311.31 | 91.15 | 143.45 | 13.67 | 97.81 |
| Single-stroke | B | 35 |  | 1.23 | 243.24 |  | 55.28 | 157.37 | 23.55 | 89.39 |
| Single-stroke | B | 36 |  | 0.81 | 324.53 |  | 103.91 | 158.24 | 24.15 | 90.13 |
| Single-stroke | B | 37 |  | 1.07 | 293.29 |  | 47.85 | 155.45 | 20.23 | 82.73 |
| Single-stroke | B | 38 |  | 2.47 | 258.78 |  | 88.84 | 149.45 | 28.45 | 88.48 |
| To-and-Fro single | B | 39 |  | 2.33 | 300.91 |  | 68.67 | 156.53 | 30.62 | 88.13 |
| Single-stroke | B | 40 |  | 2.24 | 238.23 |  | 92.53 | 134.32 | 26.14 | 75.59 |
| Single-stroke | B | 41 |  | 2.10 | 249.84 |  | 81.30 | 149.88 | 29.9 | 82.61 |
| To-and-Fro single | B | 42 |  | 2.41 | 306.10 |  | 106.21 | 150.78 | 33.56 | 90.13 |
| Single-stroke | B | 43 |  | 2.12 | 212.77 |  | 40.72 | 148.07 | 32.09 | 90.61 |
| Single-stroke | B | 44 |  | 2.89 | 330.75 |  | 91.43 | 143.86 | 33.76 | 97.7 |
| Scraping double | B | 45 | A | 1.59 |  | 779.93 | 64.56 | 160.71 | 41.9 | 87.52 |
|  | B |  | B | 1.40 |  | 779.93 | 247.37 | 151.68 | 54.7 | 82.29 |
| To-and-Fro double | B | 46 | A | 1.11 |  | 350.65 | 32.85 | 168.75 | 10.01 | 86.12 |
|  | B |  | B | 1.16 |  | 350.65 | 48.20 | 162.56 | 11.22 | 84.47 |
| To-and-Fro double | B | 47 | A | 1.67 |  | 322.22 | 32.89 | 137.30 | 15.76 | 77.94 |
|  | B |  | B | 2.01 |  | 322.22 | 60.96 | 151.42 | 20.57 | 88.44 |
| Scraping single | B | 48 |  | 2.05 | 659.36 |  | 214.43 | 159.92 | 47.96 | 93.66 |
| Scraping double | B | 49 | A | 1.55 |  | 656.16 | 80.78 | 160.30 | 35.06 | 88.83 |
|  | B |  | B | 2.02 |  | 656.16 | 94.86 | 150.68 | 53.63 | 91.24 |
| Single-stroke | B | 50 |  | 1.04 | 185.40 |  | 56.96 | 148.87 | 22.07 | 91.61 |
| Single-stroke | B | 51 |  | 2.06 | 340.73 |  | 95.27 | 151.94 | 29.46 | 95.39 |
| Single-stroke | B | 52 |  | 1.93 | 366.58 |  | 113.92 | 149.42 | 41.86 | 89.23 |
| Single-stroke | B | 53 |  | 2.24 | 280.90 |  | 103.92 | 146.97 | 36.48 | 89.03 |
| Scraping single | B | 54 |  | 2.41 | 1227.7 |  | 499.55 | 155.45 | 82.71 | 83.91 |
| Scraping multiple | B | 55 | A | 1.78 |  | 502.03 | 20.17 | 169.40 | 8.43 | 86.6 |
|  | B |  | B | 1.58 |  | 502.03 | 65.58 | 170.23 | 12.39 | 87.86 |
|  | B |  | C | 1.25 |  | 502.03 | 75.05 | 169.33 | 10.81 | 89.64 |
| To-and-Fro single | B | 56 |  | 1.31 | 317.99 |  | 114.57 | 166.43 | 13.34 | 86.46 |
| To-and-Fro double | B | 57 | A | 1.16 |  | 389.16 | 50.97 | 143.17 | 24.97 | 82.68 |
|  | B |  | B | 1.50 |  | 389.16 | 92.61 | 137.69 | 40.83 | 100.44 |
| Scraping single | B | 58 |  | 2.16 | 416.75 |  | 217.83 | 137.31 | 51.25 | 85.57 |
| Single-stroke | B | 59 |  | 2.16 | 230.72 |  | 69.27 | 141.39 | 25.01 | 98.28 |
| Scraping single | B | 60 |  | 2.12 | 1119.45 |  | 253.98 | 147.96 | 123.17 | 84.19 |
| To-and-Fro single | C | 61 |  | 1.95 | 334.53 |  | 105.66 | 150.13 | 41.52 | 89.28 |
| Single-stroke | C | 62 |  | 1.49 | 340.39 |  | 56.39 | 165.35 | 19.03 | 92.38 |
| Scraping multiple | C | 63 | A | 2.85 |  | 954.23 | 87.35 | 146.76 | 109.94 | 87.27 |
|  | C |  | B | 2.60 |  | 954.23 | 54.06 | 149.93 | 97.68 | 87.80 |
|  | C |  | C | 2.60 |  | 954.23 | 34.88 | 150.3 | 58.38 | 88.51 |
| To-and-Fro double | C | 64 | A | 2.44 |  | 453.55 | 107.7 | 144.05 | 31.93 | 82.55 |
|  | C |  | B | 2.10 |  | 453.55 | 24.28 | 156.19 | 25.62 | 91.55 |
| Scraping single | C | 65 |  | 2.76 | 1043.93 |  | 232.39 | 100.59 | 120.42 | 59.36 |
| Single-stroke | C | 66 |  |  | 227.23 |  | 76.52 | 138.06 | 21.51 | 102.4 |
| To-and-Fro double | C | 67 | A | 0.97 |  | 302.00 | 39.15 | 155.14 | 25.63 | 87.17 |
|  | C |  | B | 1.34 |  | 302.00 | 36.57 | 130.59 | 26.92 | 95.23 |
| Scraping multiple | C | 68 | A | 1.31 |  | 739.84 | 59.58 | 128.64 | 47.38 | 70.14 |
|  | C |  | B | 2.22 |  | 739.84 | 102.25 | 131.23 | 69.63 | 73.01 |
| Single-stroke | C | 69 |  | 1.97 | 256.06 |  | 69.70 | 162.04 | 17.02 | 87.18 |
| To-and-Fro single | C | 70 |  | 2.15 | 428.11 |  | 176.25 | 151.18 | 50.29 | 90.01 |
| To-and-Fro single | C | 71 |  | 1.24 | 396.96 |  | 97.66 | 139.91 | 60.64 | 92.57 |
| Scraping single | C | 72 |  | 2.42 | 549.78 |  | 102.73 | 86.72 | 132.75 | 61.17 |
| Scraping single | C | 73 |  | 3.37 | 936.80 |  | 310.10 | 148.98 | 96.21 | 85.19 |
| Scraping double | C | 74 |  | 2.28 |  | 613.96 | 116.92 | 144.65 | 68.38 | 91.32 |
|  | C |  |  | 1.82 |  | 613.96 | 48.23 | 142.09 | 37.88 | 92.72 |
| Scraping single | C | 75 |  | 3.31 | 633.36 |  | 78.79 | 143.84 | 81.88 | 83.15 |
| Scraping single | C | 76 |  | 2.54 | 766.56 |  | 192.28 | 138.51 | 129.93 | 89.71 |
| To-and-Fro double | C | 77 | A | 2.2 |  | 339.64 | 36.51 | 130.88 | 37.64 | 81.45 |
|  | C |  | B | 2.23 |  | 339.64 | 43.17 | 136.95 | 44.79 | 85.42 |
| Single-stroke |  | 78 |  | 1.47 | 189.41 |  | 58.69 | 156.94 | 17.98 | 87.77 |
| To-and-Fro double | D | 79 | A | 2.04 |  | 546.45 | 49.23 | 150.29 | 17.25 | 96.03 |
|  | D |  | B | 4.14 |  | 546.45 | 78.94 | 150.29 | 36.29 | 95.38 |
| To-and-Fro single | D | 80 |  | 2.93 | 465.05 |  | 100.41 | 150.17 | 56.53 | 91.03 |
| To-and-Fro double | D | 81 | A | 1.96 |  | 418.88 | 28.27 | 150.74 | 22.58 | 92.98 |
|  | D |  | B | 2.52 |  | 418.88 | 36.76 | 142.41 | 35.44 | 89.71 |
| To-and-Fro double | D | 82 | A | 2.71 |  | 467.9 | 82.07 | 116.29 | 45.43 | 68.52 |
|  | D |  | B | 2.23 |  | 467.9 | 46.85 | 140.75 | 31.39 | 82.23 |
| Scraping single | D | 83 |  | 1.87 | 613.52 |  | 270.69 | 145.39 | 39.95 | 78.51 |
| Scraping double | D | 84 | A | 1.97 |  | 522.77 | 81.84 | 153.54 | 47.63 | 90.56 |
|  | D |  | B | 1.87 |  | 522.77 | 42.82 | 155.88 | 20.68 | 94.34 |
| Scraping single | D | 85 |  | 2.45 | 804.25 |  | 213.76 | 139.88 | 124.54 | 86.85 |
| Scraping single | D | 86 |  | 1.85 | 469.97 |  | 277.10 | 120.39 | 62.00 | 76.52 |
| To-and-Fro double | D | 87 | A | 2.35 |  | 570.91 | 209.72 | 151.25 | 45.8 | 85.71 |
|  | D |  | B | 3.05 |  | 570.91 | 74.72 | 112.82 | 55.33 | 82.03 |

**Table 2:** Type of incision, length (when possible) and micro-morphometric profile values, taken at the incision’s midpoint, of each engraved incision on artefacts from Gough’s Cave. Length of the incision (L), width of the incision at the surface (WIS), width at the bottom of the incision (WIB), opening angle of the cut (OA), depth (D) and angle of the tool inclination (ATI).

| **Type of incision** | **Incision** | **Sub-incisions** | **Length (mm)** | **WIS (µm)** | **WIS (for multiple incisions, µm)** | **WIB (µm)** | **OA (˚)** | **D (µm)** | **ATI (˚)** |
| --- | --- | --- | --- | --- | --- | --- | --- | --- | --- |
| Horse rib BS27 3QF (X-side) | | | | | | | | | |
| Single-stroke | 1 |  |  | 202.93 |  | 141.79 | 144.75 | 12.87 | 96.075 |
| Single-stroke | 2 |  |  | 529.38 |  | 105.58 | 167.58 | 30.84 | 90.21 |
| Single-stroke | 3 |  |  | 357.63 |  | 312.83 | 151.14 | 16.43 | 80.97 |
| Single-stroke | 4 |  |  | 288.39 |  | 141.18 | 153.83 | 22.91 | 86.625 |
| Single-stroke | 5 |  |  | 315.63 |  | 74.34 | 160.97 | 23.07 | 86.005 |
| Single-stroke | 6 |  |  | 320.18 |  | 204.22 | 143.11 | 26.28 | 88.995 |
| Single-stroke | 7 |  |  | 178.94 |  | 39.55 | 122.19 | 43.54 | 82.465 |
| Single-stroke | 8 |  |  | 333.77 |  | 89.13 | 169.36 | 10.94 | 92.26 |
| Single-stroke | 9 |  |  | 308.95 |  | 196.71 | 149.13 | 16.76 | 82.835 |
| Single-stroke | 10 |  |  | 322.51 |  | 66.49 | 167.67 | 16.05 | 90.925 |
| To-and-Fro double | 11 | A |  |  | 93.88 | 42.73 | 147.83 | 17.2 | 84.825 |
|  |  | B |  |  | 93.88 | 25.34 | 155.79 | 12.29 | 97.285 |
| Single-stroke | 12 |  |  | 203.73 |  | 56.66 | 153.33 | 21.84 | 92.795 |
| To-and-Fro double | 13 | A |  |  | 247.89 | 105.86 | 137.55 | 28.32 | 98.265 |
|  |  | B |  |  |  | 42.23 | 116.03 | 32.28 | 110.965 |
| Single-stroke | 14 |  |  | 184.25 |  | 51.3 | 159.18 | 16.18 | 88.14 |
| Single-stroke | 15 |  |  | 241.98 |  | 64.15 | 155.91 | 22.22 | 92.815 |
| Single-stroke | 16 |  |  | 126.9 |  | 70.54 | 126.17 | 13.98 | 100.185 |
| Single-stroke | 17 |  |  | 185.19 |  | 71.24 | 156.87 | 15.68 | 89.265 |
| Single-stroke | 18 |  |  | 312.76 |  | 116.41 | 158.19 | 25.38 | 89.095 |
| Single-stroke | 19 |  |  | 220.73 |  | 140.62 | 148.95 | 14.52 | 92.995 |
| Single-stroke | 20 |  |  | 304.05 |  | 116.01 | 162.64 | 19.76 | 89.67 |
| Single-stroke | 21 |  |  | 274.14 |  | 149.69 | 161.13 | 15.27 | 89.055 |
| Single-stroke | 22 |  |  | 415.49 |  | 105.49 | 167.01 | 24.17 | 91.095 |
| Single-stroke | 23 |  |  | 425.73 |  | 115.18 | 154.77 | 31.44 | 83.035 |
| Single-stroke | 24 |  |  | 299.09 |  | 140.88 | 160.29 | 14.02 | 85.085 |
| Single-stroke | 25 |  |  | 178.82 |  | 75.35 | 152.84 | 19.78 | 84.31 |
| Single-stroke | 26 |  |  | 271.01 |  | 57.79 | 151.32 | 16.57 | 79.86 |
| Single-stroke | 27 |  |  | 241.66 |  | 145.73 | 134.3 | 34.09 | 93.27 |
| Single-stroke | 28 |  |  | 172.84 |  | 65.39 | 156.7 | 15.15 | 92.32 |
| Single-stroke | 29 |  |  | 455.89 |  | 115.78 | 169.63 | 18.58 | 88.835 |
| Single-stroke | 30 |  |  | 482.32 |  | 109.53 | 165.72 | 20.98 | 93.38 |
| Single-stroke | 31 |  |  | 191.09 |  | 155.14 | 114 | 16.22 | 89.68 |
| Single-stroke | 32 |  |  | 384.94 |  | 90.77 | 165.16 | 27.26 | 89.03 |
| Single-stroke | 33 |  |  | 172.44 |  | 25.65 | 165.91 | 10.27 | 88.605 |
| Single-stroke | 34 |  |  | 214.4 |  | 124.95 | 130.17 | 21.16 | 76.215 |
| Single-stroke | 35 |  |  | 359.74 |  | 89.73 | 165.16 | 15.02 | 93.39 |
| Single-stroke | 36 |  |  | 258.35 |  | 114.72 | 164.74 | 11.46 | 87.7 |
| Single-stroke | 37 |  |  | 179.27 |  | 77.56 | 154.81 | 15.44 | 91.775 |
| Single-stroke | 38 |  |  | 176.77 |  | 76.27 | 130.59 | 18.27 | 92.145 |
| Single-stroke | 39 |  |  | 364.83 |  | 51.7 | 165.75 | 24.18 | 90.855 |
| Single-stroke | 40 |  |  | 320.15 |  | 150.41 | 142.9 | 16.39 | 75.69 |
| Single-stroke | 41 |  |  | 252.43 |  | 118.91 | 154.34 | 20.63 | 91.03 |
| Single-stroke | 42 |  |  | 170.92 |  | 103.49 | 147.93 | 9.49 | 95.295 |
| Single-stroke | 43 |  |  | 232.59 |  | 45.99 | 143.42 | 36.39 | 88.08 |
| Single-stroke | 44 |  |  | 280.49 |  | 60.29 | 165.63 | 15.23 | 91.205 |
| Horse rib BS27 3QF (notches side) | | | | | | | | | |
| Single-stroke | 1 |  | 2.54 | 212.85 |  | 62.08 | 138.12 | 32.85 | 95.52 |
| Single-stroke | 2 |  | 2.49 | 299.47 |  | 167.59 | 152.64 | 28.06 | 83.83 |
| Single-stroke | 3 |  | 2.52 | 210.29 |  | 113.87 | 136.29 | 20.42 | 96.145 |
| To-and-Fro double | 4 | A | 2.42 |  | 500.44 | 35.79 | 118.83 | 80.22 | 83.675 |
|  |  | B | 2.19 |  | 500.44 | 116.61 | 122.75 | 92.35 | 82.585 |
| Single-stroke | 5 |  | 2.09 | 394.05 |  | 88.47 | 157.33 | 31.81 | 84.555 |
| Single-stroke | 6 |  | 1.95 | 264.94 |  | 85.61 | 153.68 | 18.84 | 81.08 |
| Single-stroke | 7 |  | 1.9 | 241.73 |  | 131.14 | 155.58 | 21.01 | 86.17 |
| To-and-Fro double | 8 | A | 2.04 |  | 298.13 | 95.17 | 141.7 | 22.9 | 90.39 |
|  |  | B | 2.21 |  | 298.13 | 41.18 | 149.03 | 21.39 | 98.235 |
| Single-stroke | 9 |  | 2.38 | 316.22 |  | 142.88 | 136.71 | 36.47 | 80.715 |
| Single-stroke | 10 |  | 2.91 | 170.79 |  | 54.96 | 127.94 | 28.02 | 81.79 |
| Single-stroke | 11 |  | 2.93 | 271.82 |  | 43.55 | 151.53 | 30.97 | 88.405 |
| Single-stroke | 12 |  | 3.37 | 259.26 |  | 35.97 | 156.63 | 29.08 | 88.165 |
| Single-stroke | 13 |  | 3.25 | 377.17 |  | 130.21 | 164.32 | 21.45 | 89 |
| Single-stroke | 14 |  | 3.39 | 433.94 |  | 74.7 | 166.52 | 25.2 | 89.09 |
| To-and-Fro double | 15 | A | 2.73 |  | 384.91 | 69.81 | 151.76 | 13.71 | 80.66 |
|  |  | B | 2.86 |  | 384.91 | 69.8 | 165.9 | 15.73 | 90.17 |
| Single-stroke | 16 |  | 2.93 | 234.93 |  | 55.18 | 151.11 | 22.97 | 80.715 |
| Single-stroke | 17 |  | 3.14 | 232.69 |  | 44.4 | 165.72 | 14.17 | 88.64 |
| To-and-Fro double | 18 | A | 3.19 |  | 332.6 | 41.37 | 150.63 | 25.92 | 93.125 |
|  |  | B | 2.84 |  | 332.6 | 44.77 | 161.16 | 22.85 | 93.72 |
| Single-stroke | 19 |  | 3.23 | 300.01 |  | 62.17 | 152.2 | 32.65 | 87.38 |
| Single-stroke | 20 |  | 2.44 | 288.29 |  | 43.72 | 168.89 | 16.15 | 90.205 |
| Single-stroke | 21 |  | 2.91 | 361.0 |  | 75.5 | 147.94 | 47.05 | 85.37 |
| Single-stroke | 22 |  | 2.63 | 208.93 |  | 65.16 | 167.69 | 9.3 | 91.285 |
| Single-stroke | 23 |  | 2.8 | 313.93 |  | 104.55 | 167.14 | 14.54 | 89.77 |
| Single-stroke | 24 |  | 2.88 | 269.57 |  | 77.86 | 164.53 | 13.69 | 86.615 |
| Single-stroke | 25 |  | 2.82 | 384.74 |  | 65.74 | 166.5 | 20.46 | 88.19 |
| Single-stroke | 26 |  | 2.75 | 362.95 |  | 54.16 | 169.68 | 15.18 | 91.58 |
| Single-stroke | 27 |  | 3.07 | 289.68 |  | 51.18 | 138.23 | 46.11 | 92.275 |
| Single-stroke | 28 |  | 3.25 | 385.54 |  | 59.16 | 149.96 | 50.85 | 89.7 |
| Single-stroke | 29 |  | 2.75 | 379.29 |  | 87.38 | 147.44 | 47.52 | 86.81 |
| Single-stroke | 30 |  | 2.87 | 467.13 |  | 116.22 | 162.16 | 31.54 | 86.25 |
| Single-stroke | 31 |  | 3.55 | 219.36 |  | 41.61 | 135.24 | 33.02 | 97.93 |
| Single-stroke | 32 |  | 2.57 | 267.57 |  | 44.28 | 143.28 | 37.73 | 93.21 |
| Single-stroke | 33 |  | 2.5 | 404.3 |  | 103.91 | 163.89 | 22.97 | 92.615 |
| Single-stroke | 34 |  | 2.78 | 407.19 |  | 98.38 | 141.86 | 52.3 | 94.03 |
| Single-stroke | 35 |  | 3.16 | 461.78 |  | 55.59 | 141.46 | 67.82 | 95.32 |
| Single-stroke | 36 |  | 2.41 | 415.25 |  | 106.8 | 159.06 | 36.19 | 92.54 |
| Single-stroke | 37 |  | 2.45 | 213.81 |  | 43.64 | 135.6 | 32.24 | 86.9 |
| Single-stroke | 38 |  | 3.19 | 415.65 |  | 76.67 | 145.1 | 63.72 | 89.38 |
| Single-stroke | 39 |  | 3.75 | 443.28 |  | 106.92 | 132.74 | 79.27 | 79.71 |
| Single-stroke | 40 |  | 2.84 | 492.29 |  | 90.01 | 168.16 | 25.53 | 88.42 |
| Single-stroke | 41 |  | 3.24 | 550.1 |  | 88.55 | 165.11 | 30.62 | 87.745 |
| Single-stroke | 42 |  | 2.95 | 455.59 |  | 121.21 | 161.38 | 29.21 | 84.19 |
| Single-stroke | 43 |  | 3.32 | 343.76 |  | 145.61 | 148.85 | 29.53 | 97.165 |
| Single-stroke | 44 |  | 3.27 | 304.78 |  | 66.94 | 151.7 | 37.4 | 86.97 |
| Single-stroke | 45 |  | 3.81 | 253.76 |  | 105.66 | 139.55 | 31.55 | 82.085 |
| To-and-Fro double | 46 | A | 2.94 |  | 672.11 | 148.21 | 142.04 | 94.66 | 84.71 |
|  |  | B | 2.98 |  | 672.11 | 65.48 | 135.27 | 74.36 | 93.205 |
| Single-stroke | 47 |  | 3.87 | 219.1 |  | 52.48 | 152.83 | 19.9 | 41.115 |
| Single-stroke | 48 |  | 3.81 | 254.55 |  | 60.02 | 142.2 | 40.92 | 90.18 |
| Single-stroke | 49 |  | 4.13 | 212.41 |  | 107.02 | 102.37 | 59.86 | 86.265 |
| Single-stroke | 50 |  | 3.13 | 210.22 |  | 67.24 | 127.21 | 39.82 | 90.045 |
| Single-stroke | 51 |  | 3.79 | 341.84 |  | 85.84 | 130.7 | 65.88 | 96.4 |
| Single-stroke | 52 |  | 2.81 | 388.72 |  | 127.91 | 158.21 | 29.36 | 82.375 |
| Single-stroke | 53 |  |  | 386.84 |  | 128.81 | 152.87 | 30.47 | 96.175 |
| Tibia of hare (BS27 3QF) | | | | | | | | | |
| Single-stroke | 1 |  | 1.13 | 411.3 |  | 87.77 | 121.19 | 105.17 | 95.585 |
| Single-stroke | 2 |  | 1.15 | 367.65 |  | 80.73 | 143.16 | 52.58 | 92.05 |
| Single-stroke | 3 |  | 1.51 | 336.09 |  | 133.05 | 129.43 | 66.15 | 92.565 |
| Single-stroke | 4 |  | 1.3 | 316.49 |  | 120.33 | 132.92 | 56.58 | 97.83 |
| Single-stroke | 5 |  | 1.51 | 326.53 |  | 115.08 | 115.62 | 88.49 | 84.42 |
| Single-stroke | 6 |  | 1.48 | 295.88 |  | 72.34 | 101.49 | 111.62 | 87.325 |
| Single-stroke | 7 |  | 1.51 | 421.81 |  | 127.14 | 136.8 | 66.19 | 89.29 |
| Single-stroke | 8 |  | 1.53 | 398.77 |  | 111.18 | 124.86 | 81.97 | 93.75 |
| Single-stroke | 9 |  | 1.4 | 353.48 |  | 153 | 115.04 | 73.86 | 87.63 |
| Single-stroke | 10 |  | 1.51 | 477.44 |  | 186.83 | 109.2 | 137.77 | 88.54 |
| Single-stroke | 11 |  | 1.4 | 395.34 |  | 74.63 | 161.93 | 31.64 | 91.315 |
| Single-stroke | 12 |  | 1.4 | 329.16 |  | 77.19 | 139.08 | 52.41 | 94.61 |
| Single-stroke | 13 |  | 1.45 | 376.37 |  | 111.64 | 140.37 | 55.78 | 87.485 |
| Single-stroke | 14 |  | 1.04 | 349.72 |  | 96.6 | 140.62 | 53.89 | 87.55 |
| Single-stroke | 15 |  | 1.14 | 415.85 |  | 180.62 | 140.84 | 54.42 | 86.65 |

**Table 3:** Micro-morphometric profile values, taken at the cut mark’s midpoint, of filleting marks on human remains from Gough’s Cave. Width of the incision at the surface (WIS), width at the bottom of the incision (WIB), opening angle of the cut (OA), depth (D) and angle of the tool inclination (ATI).

| Museum number | Bone element | Cut-mark no | WIS (µm) | WIB (µm) | OA (˚) | D (µm) | ATI (˚) |
| --- | --- | --- | --- | --- | --- | --- | --- |
| GC2 | frontal (right of R orbit) | 1 | 337.11 | 97.69 | 163.4 | 24.29 | 90.56 |
|  |  | 2 | 268.01 | 114.12 | 164.54 | 12.00 | 87.10 |
|  |  | 3 | 241.56 | 86.72 | 153.21 | 24.23 | 91.19 |
|  |  | 4 | 197.68 | 38.61 | 151.78 | 16.7 | 85.49 |
|  |  | 5 | 84.60 | 36.90 | 127.39 | 11.53 | 79.78 |
|  |  | 6 | 314.74 | 72.57 | 154.07 | 22.65 | 82.92 |
| GC86(55) | right temporal - zygomatic process | 1 | 236.08 | 154.58 | 119.49 | 26.68 | 94.605 |
|  |  | 2 | 195.55 | 92.14 | 163.39 | 12.48 | 87.195 |
|  |  | 3 | 452.87 | 245.07 | 155.60 | 33.53 | 87.28 |
|  |  | 4 | 140.88 | 63.49 | 147.47 | 13.38 | 87.225 |
|  |  | 5 | 124.05 | 77.09 | 114.92 | 15.78 | 85.29 |
|  |  | 6 | 205.61 | 149.98 | 104.83 | 21.47 | 77.005 |
|  |  | 7 | 263.45 | 130.51 | 129.86 | 28.27 | 76.72 |
|  |  | 8 | 152.18 | 34.98 | 121.03 | 30.09 | 76.815 |
| GC7 1,1-2 | right parietal | 1 | 468.76 | 105.51 | 152.28 | 46.89 | 88.14 |
|  |  | 2 | 711.04 | 110.57 | 168.7 | 29.59 | 87.46 |
|  |  | 3 | 734.84 | 177.00 | 167.95 | 56.17 | 89.05 |
|  |  | 4 | 221.99 | 129.77 | 134.51 | 32.92 | 83.93 |
|  |  | 5 | 248.12 | 64.93 | 140.56 | 35.13 | 89.98 |
|  |  | 6 | 656.64 | 359.37 | 144.42 | 56.83 | 88.02 |
|  |  | 7 | 386.72 | 95.80 | 122.37 | 53.45 | 73.94 |
|  |  | 8 | 491.97 | 240.05 | 160.53 | 40.07 | 87.55 |
|  |  | 9 | 221.41 | 142.02 | 143.73 | 18.82 | 84.41 |
|  |  | 10 | 258.40 | 79.10 | 147.30 | 30.54 | 87.42 |
| M54133 | Mandible | 1 | 323.49 | 214.75 | 111.61 | 43.59 | 82.01 |
|  |  | 2 | 185.33 | 69.81 | 134.06 | 22.22 | 104.06 |
|  |  | 3 | 280.74 | 64.53 | 152.59 | 26.28 | 85.29 |
|  |  | 4 | 239.94 | 44.37 | 162.69 | 17.01 | 91.69 |
|  |  | 5 | 206.34 | 58.07 | 161.2 | 16.82 | 95.60 |
|  |  | 6 | 235.01 | 77.12 | 166.89 | 9.22 | 92.27 |
|  |  | 7 | 196.08 | 62.05 | 144.62 | 25.63 | 85.08 |
|  |  | 8 | 142.11 | 31.76 | 157.18 | 10.23 | 93.38 |
|  |  | 9 | 490.70 | 291.72 | 136.46 | 54.40 | 92.16 |
|  |  | 10 | 378.61 | 144.25 | 155.79 | 43.91 | 90.47 |
| M54012 | Rib 6th right | 1 | 241.42 | 131.37 | 73.06 | 69.21 | 74.3 |
| M54015 | Rib 11 - outer sternal surface | 1 | 366.66 | 123.03 | 123.46 | 86.54 | 90.87 |
|  |  | 2 | 367.79 | 71.09 | 131.58 | 89.09 | 89.13 |
|  |  | 3 | 190.15 | 70.45 | 112.06 | 51.55 | 94.15 |
|  |  | 4 | 189.77 | 51.09 | 126.41 | 49.93 | 86.95 |
|  |  | 5 | 286.53 | 125.04 | 129.12 | 55.51 | 92.38 |
|  | Rib 11 - outer surface near vertebral end | 6 | 192.61 | 66.57 | 103.75 | 39.93 | 66.27 |
|  |  | 7 | 199.07 | 84.07 | 110.1 | 58.15 | 94.30 |
|  |  | 8 | 359.83 | 204.63 | 144.12 | 34.08 | 85.32 |
|  |  | 9 | 168.59 | 61.4 | 110.26 | 42.47 | 90.64 |
|  |  | 10 | 150.77 | 47.65 | 133.78 | 25.06 | 121.00 |
|  |  | 11 | 157.49 | 53.39 | 125.79 | 29.87 | 128.04 |
|  |  | 12 | 185.34 | 49.48 | 130.84 | 29.40 | 84.49 |
| M54056 | Scapula | 1 | 303.78 | 116.4 | 84.93 | 94.25 | 86.81 |
|  |  | 2 | 327.54 | 123.5 | 110.28 | 75.49 | 83.49 |
|  |  | 3 | 320.89 | 134.1 | 159.21 | 20.57 | 89.01 |
|  |  | 4 | 241.42 | 58.11 | 151.01 | 22.95 | 89.86 |
|  |  | 5 | 218.10 | 52.74 | 144.47 | 24.27 | 94.98 |
|  |  | 6 | 354.37 | 179.85 | 159.68 | 27.84 | 90.11 |
|  |  | 7 | 350.62 | 73.81 | 149.61 | 32.16 | 93.63 |
|  |  | 8 | 310.54 | 113.70 | 145.71 | 28.32 | 86.90 |
|  |  | 9 | 328.72 | 71.53 | 158.36 | 26.02 | 92.05 |
|  |  | 10 | 282.57 | 58.56 | 156.54 | 21.14 | 93.36 |
|  |  | 11 | 229.30 | 64.40 | 142.73 | 23.9 | 89.00 |
|  |  | 12 | 252.73 | 47.63 | 144.28 | 32.64 | 90.62 |
|  |  | 13 | 298.87 | 83.41 | 150.28 | 25.56 | 90.89 |
|  |  | 14 | 279.69 | 73.48 | 141.66 | 34.66 | 87.75 |
|  |  | 15 | 289.25 | 93.46 | 154.62 | 19.08 | 96.31 |
|  |  | 16 | 238 | 43.52 | 160.17 | 14.44 | 85.365 |
| M54055 | Clavicle | 1 | 246.3 | 64.58 | 156.7 | 15.24 | 94.68 |
|  |  | 2 | 207.83 | 54.7 | 141.61 | 35.03 | 91.90 |
|  |  | 3 | 82.45 | 25.26 | 145.86 | 11.03 | 93.58 |
|  |  | 4 | 87.32 | 42.9 | 150.35 | 8.04 | 98.47 |
|  |  | 5 | 439.89 | 111.13 | 127.19 | 95.35 | 94.79 |
|  |  | 6 | 107.70 | 68.64 | 138.41 | 8.65 | 94.97 |
|  |  | 7 | 194.15 | 47.99 | 129.70 | 40.36 | 92.43 |
| M54062 | Humerus | 1 | 309.13 | 121.87 | 152.44 | 25.47 | 98.28 |
|  |  | 2 | 183.56 | 36.07 | 148.95 | 14.32 | 97.52 |
|  |  | 3 | 247 | 87.8 | 135.58 | 32.77 | 97.98 |
|  |  | 4 | 295.46 | 40.00 | 155.67 | 18.08 | 98.97 |
|  |  | 5 | 88.93 | 30.93 | 138.01 | 10.1 | 92.32 |
|  |  | 6 | 273.75 | 48.2 | 139.76 | 38.5 | 96.99 |
|  |  | 7 | 239.76 | 63.75 | 151.72 | 18.7 | 89.39 |
|  |  | 8 | 71.21 | 21.21 | 144.36 | 8.65 | 97.38 |
|  |  | 9 | 141.05 | 30.31 | 145.92 | 19.79 | 89.89 |
|  |  | 10 | 126.46 | 43.23 | 160.06 | 8.91 | 92.5 |
|  |  | 11 | 133.03 | 51.50 | 142.86 | 13.55 | 97.02 |
|  |  | 12 | 104.83 | 42.81 | 97.47 | 24.77 | 89.03 |
|  |  | 13 | 218.99 | 59.74 | 122.25 | 51.78 | 91.55 |
|  |  | 14 | 97.17 | 44.60 | 56.77 | 79.06 | 95.38 |
|  |  | 15 | 159.80 | 49.29 | 140.56 | 18.65 | 95.74 |
|  |  | 16 | 223.30 | 58.35 | 133.96 | 37.00 | 96.03 |
|  |  | 17 | 200.54 | 44.60 | 145.31 | 27.77 | 88.615 |
| M 54063 | Humerus | 1 | 331.84 | 86.61 | 109.05 | 77.82 | 75.915 |
|  |  | 2 | 361.29 | 121.32 | 123.38 | 73.00 | 89.59 |
|  |  | 3 | 707.49 | 402.69 | 124.96 | 79.95 | 77.15 |
| M54061 | Humerus | 1 | 404.41 | 97.95 | 133.58 | 69.3 | 82.75 |
|  |  | 2 | 169.42 | 77.98 | 144.66 | 19.1 | 88.23 |
|  |  | 3 | 262.04 | 142.32 | 113.63 | 53.66 | 74.23 |
|  |  | 4 | 178.19 | 65.43 | 147.44 | 23.61 | 88.04 |
|  |  | 5 | 167.82 | 92.29 | 119.30 | 29.30 | 79.76 |
|  |  | 6 | 165.61 | 66.32 | 117.16 | 26.99 | 73.54 |
|  |  | 7 | 257.03 | 86.93 | 136.35 | 35.71 | 84.30 |
| M54071 | Radius | 1 | 143.79 | 42.59 | 145.06 | 15.54 | 89.22 |
|  |  | 2 | 142.78 | 101.15 | 141.02 | 8.74 | 86.21 |
|  |  | 3 | 214.65 | 72.28 | 128.88 | 34.24 | 78.22 |
|  |  | 4 | 285.87 | 112.41 | 131.38 | 45.14 | 89.31 |
|  |  | 5 | 285.69 | 53.57 | 132.51 | 32.74 | 74.09 |
| M54074 | Radius | 1 | 314.36 | 140.66 | 163.97 | 16.79 | 89.56 |
|  |  | 2 | 397.47 | 109.66 | 149.7 | 15.3 | 77.46 |
|  |  | 3 | 257.56 | 136.06 | 143.86 | 12.29 | 75.19 |
|  |  | 4 | 349.29 | 43.76 | 169.25 | 16.68 | 88.05 |
|  |  | 5 | 234.32 | 70 | 152.64 | 23.73 | 85.21 |
|  |  | 6 | 164.86 | 83.04 | 120.86 | 19.04 | 98.68 |
|  |  | 7 | 408.29 | 191.99 | 151.54 | 37.95 | 90.55 |
|  |  | 8 | 458.49 | 154.92 | 161.35 | 25.21 | 95.56 |
|  |  | 9 | 266.11 | 153.57 | 140.62 | 22.69 | 88.27 |
|  |  | 10 | 196.52 | 46.54 | 160.37 | 14.73 | 89.035 |
|  |  | 11 | 180.82 | 50.32 | 150.46 | 18.97 | 88.7 |
|  |  | 12 | 205.24 | 60.22 | 137.65 | 30.1 | 99.25 |
|  |  | 13 | 257.38 | 173.11 | 135.8 | 24.87 | 89.6 |
|  |  | 14 | 184.24 | 144.63 | 142.86 | 15.1 | 102.25 |
|  |  | 15 | 431.53 | 322.23 | 156.95 | 32.43 | 98.78 |
|  |  | 16 | 213.37 | 140.07 | 134.16 | 15.33 | 77.25 |
|  |  | 17 | 161.1 | 79.73 | 135.43 | 15.14 | 99.49 |
|  |  | 18 | 260.36 | 173.04 | 147.62 | 14.38 | 96.3 |
|  |  | 19 | 110.76 | 43.48 | 140.81 | 10.85 | 97.01 |
|  |  | 20 | 264.41 | 128.48 | 138.04 | 30.78 | 86.77 |
|  |  | 21 | 209.28 | 68.83 | 140.47 | 25.87 | 98.09 |
|  |  | 22 | 229.71 | 78.62 | 136.68 | 34.46 | 85.55 |
|  |  | 23 | 128.29 | 52.86 | 125.94 | 23.54 | 82.9 |
|  |  | 24 | 168.43 | 93.4 | 110.18 | 32.44 | 87.96 |
|  |  | 25 | 80.22 | 19.67 | 113.18 | 23.38 | 83.44 |
|  |  | 26 | 136.73 | 54.03 | 126.28 | 18.7 | 80.32 |
|  |  | 27 | 177.84 | 36.71 | 145.39 | 20.88 | 79.80 |
|  |  | 28 | 328.49 | 118.87 | 143.62 | 41.02 | 88.5 |
|  |  | 29 | 125.47 | 60.79 | 129.03 | 13.4 | 79.745 |
|  |  | 30 | 135.4 | 31.1 | 153.77 | 14.88 | 92.695 |
|  |  | 31 | 159.21 | 50.67 | 133.31 | 23.78 | 86.975 |
| M54068 | Ulna | 1 | 245.32 | 77.81 | 119.37 | 49.9 | 91.32 |
|  |  | 2 | 250.11 | 89.52 | 140.51 | 45.46 | 90.43 |
|  |  | 3 | 303.15 | 77.87 | 122.54 | 45.91 | 75.26 |
|  |  | 4 | 335.02 | 96.81 | 113.72 | 55.86 | 71.37 |
|  |  | 5 | 182.94 | 108.28 | 139.34 | 12.28 | 100.08 |
|  |  | 6 | 95.64 | 45.35 | 154.9 | 6.85 | 95.79 |
|  |  | 7 | 90.89 | 19.28 | 151.74 | 8.12 | 84.28 |
|  |  | 8 | 347.93 | 115.22 | 97.57 | 103.86 | 83.95 |
| M96560 | Femur | 1 | 266.47 | 108.72 | 143.4 | 28.62 | 85.23 |
|  |  | 2 | 208.24 | 52.7 | 140.58 | 26.78 | 83.77 |
|  |  | 3 | 190.99 | 77.1 | 146.09 | 18.43 | 86.815 |
|  |  | 4 | 302.31 | 45.51 | 135.2 | 54.63 | 84.39 |
|  |  | 5 | 247.62 | 70.28 | 95.21 | 81.48 | 91.045 |
|  |  | 6 | 266.8 | 70.62 | 139.77 | 40.63 | 91.715 |
|  |  | 7 | 196.17 | 71.42 | 158.95 | 12.02 | 87.975 |
|  |  | 8 | 201.5 | 91.86 | 128.09 | 19.61 | 106.07 |
|  |  | 9 | 219.49 | 59.62 | 160.74 | 13.88 | 92.29 |
|  |  | 10 | 193.74 | 83.75 | 159.98 | 12.72 | 90.08 |
|  |  | 11 | 310.79 | 63.11 | 148.93 | 29.47 | 98.29 |
|  |  | 12 | 131.45 | 79.93 | 151.38 | 5.74 | 98.14 |
|  |  | 13 | 185.14 | 34.14 | 142.23 | 15.92 | 81.42 |
|  |  | 14 | 412.8 | 121.19 | 126.58 | 71.59 | 98.42 |
| M54120 | Femur | 1 | 119.87 | 71.88 | 128.33 | 15.31 | 89.79 |
|  |  | 2 | 169.04 | 80.11 | 138.15 | 20.16 | 91.41 |
|  |  | 3 | 111.08 | 34.22 | 132.77 | 16.4 | 93.09 |
|  |  | 4 | 176.6 | 60.97 | 142.26 | 21.8 | 86.18 |
|  |  | 5 | 183.94 | 81.01 | 146.89 | 18.46 | 84.89 |
|  |  | 6 | 196.23 | 84.98 | 158.35 | 13.79 | 90.60 |
|  |  | 7 | 132.34 | 94.64 | 124.26 | 12.21 | 95.25 |
|  |  | 8 | 171.16 | 60.58 | 148.79 | 17.95 | 90.92 |
|  |  | 9 | 122.64 | 48.69 | 138.88 | 12.2 | 80.56 |
|  |  | 10 | 198.43 | 108.79 | 147.66 | 14.22 | 88.06 |
|  |  | 11 | 242.76 | 86.96 | 157.27 | 17.4 | 93.53 |
|  |  | 12 | 434.99 | 161.96 | 143.44 | 51.91 | 88.74 |
|  |  | 13 | 126.91 | 37.82 | 134.41 | 20.09 | 98.68 |
|  |  | 14 | 186.28 | 49.17 | 131.17 | 34.61 | 90.08 |
|  |  | 15 | 200.14 | 52.21 | 136.1 | 33.95 | 95.64 |
| M54085 | Femur | 1 | 197.87 | 86.34 | 127.02 | 29.93 | 79.07 |
|  |  | 2 | 292.87 | 31.22 | 138.59 | 44.97 | 82.56 |
|  |  | 3 | 198.79 | 33.5 | 135.93 | 20.59 | 76.19 |
|  |  | 4 | 438.95 | 61.28 | 121.68 | 74.79 | 73.91 |
| M54125 | Femur | 1 | 382.65 | 168.97 | 128.89 | 53.09 | 79.60 |
|  |  | 2 | 392.22 | 58.4 | 148.49 | 48.88 | 95.68 |
| M54126 | Tibia | 1 | 411.38 | 126.54 | 129.52 | 73.59 | 84.54 |
|  |  | 2 | 515.77 | 94.75 | 121.0 | 95.91 | 93.36 |
|  |  | 3 | 359.21 | 126.05 | 112.5 | 81.22 | 80.2 |
|  |  | 4 | 280.09 | 96.26 | 117.54 | 60.79 | 88.32 |
| M54097 | Metatarsal | 1 | 311.66 | 123.27 | 148.31 | 16.59 | 100.36 |
|  |  | 2 | 93.37 | 23.17 | 151.01 | 9.98 | 93.26 |
|  |  | 3 | 188.67 | 25.69 | 123.01 | 19.06 | 67.52 |
|  |  | 4 | 238.28 | 146.73 | 142.23 | 10.08 | 98.22 |
|  |  | 5 | 126.56 | 29.96 | 132.15 | 17.97 | 76.92 |
|  |  | 6 | 99.78 | 32.99 | 153.45 | 9.78 | 86.51 |
|  |  | 7 | 152.92 | 78.54 | 147.9 | 10.34 | 82.95 |
|  |  | 8 | 151.78 | 101.38 | 136.97 | 9.56 | 92.73 |
|  |  | 9 | 106.21 | 30.88 | 146.26 | 14.02 | 84.34 |
|  |  | 10 | 144.56 | 67.23 | 149.66 | 10.23 | 84.55 |
|  |  | 11 | 172.63 | 55.41 | 142.73 | 24.4 | 84.51 |
|  |  | 12 | 455.34 | 240.87 | 163.36 | 18.72 | 91.22 |
| M54098 | Metatarsal | 1 | 178.39 | 102.88 | 74.21 | 68.54 | 64.99 |
|  |  | 2 | 571.21 | 99.59 | 155.45 | 58.17 | 89.24 |
|  |  | 3 | 717.87 | 221.49 | 152.67 | 62.52 | 86.32 |
|  |  | 4 | 566.28 | 92.98 | 155.35 | 58.33 | 87.59 |
|  |  | 5 | 408.52 | 114.37 | 141.72 | 52.07 | 81.67 |
|  |  | 6 | 236.53 | 59.91 | 152.6 | 26.64 | 89.2 |
|  |  | 7 | 667.95 | 270.85 | 137.2 | 71.1 | 79.38 |
|  |  | 8 | 561.5 | 168.55 | 149.56 | 54.51 | 88.44 |
|  |  | 9 | 271.6 | 64.58 | 124.38 | 95.34 | 74.29 |
|  |  | 10 | 509.32 | 138.57 | 131.93 | 103.17 | 81.60 |
|  |  | 11 | 337.02 | 80.9 | 153.14 | 21.77 | 80.62 |
|  |  | 12 | 256.23 | 84.34 | 83.92 | 67.75 | 60.48 |
|  |  | 13 | 407.43 | 74.21 | 141.56 | 23.54 | 75.85 |
|  |  | 14 | 347.11 | 126.75 | 146.69 | 33.83 | 84.34 |
|  |  | 15 | 313.63 | 87.34 | 158.16 | 27.65 | 89.2 |

**Table 4:** Micro-morphometric profile values, taken at the cut mark’s midpoint, of filleting marks on non-human remains from Gough’s Cave. Width of the incision at the surface (WIS), width at the bottom of the incision (WIB), opening angle of the cut (OA), depth (D) and angle of the tool inclination (ATI).

| SPECIES | grave/details/no | Element | Cut-mark no | WIS (µm) | WIB (µm) | OA (˚) | D (µm) | ATI (˚) |
| --- | --- | --- | --- | --- | --- | --- | --- | --- |
| *Equus ferus* | M50001 | Mandible | 1 | 536.16 | 167.3 | 157.56 | 44.32 | 90.85 |
|  |  |  | 2 | 320.5 | 101.81 | 165.43 | 27.68 | 90.145 |
|  |  |  | 3 | 275.23 | 76.48 | 164.66 | 14.44 | 88.85 |
|  |  |  | 4 | 341.08 | 80.63 | 161.98 | 21.3 | 91.58 |
|  |  |  | 5 | 381.6 | 143.31 | 165.31 | 18.34 | 89.85 |
| *Equus ferus* | M50030 | 1st phalange | 1 | 447.22 | 148.58 | 101.65 | 74.16 | 63.56 |
|  |  |  | 2 | 525.73 | 238.8 | 120.33 | 99.06 | 85.39 |
|  |  |  | 3 | 390.8 | 106.67 | 94.83 | 119.59 | 73.49 |
|  |  |  | 4 | 146.81 | 78.84 | 139.46 | 30.62 | 84.28 |
|  |  |  | 5 | 200.18 | 57.43 | 162.64 | 26.52 | 90.54 |
|  |  |  | 6 | 253.59 | 84.35 | 116.04 | 29.9 | 76.15 |
|  |  |  | 7 | 336.48 | 75.94 | 113.55 | 81.2 | 78.12 |
|  |  |  | 8 | 284 | 103.09 | 116.67 | 80.19 | 87.54 |
|  |  |  | 9 | 186.62 | 58.81 | 114.73 | 63.36 | 79.85 |
|  |  |  | 10 | 174.8 | 50.52 | 130.89 | 35.61 | 90.14 |
|  |  |  | 11 | 236.72 | 106.13 | 124.82 | 36.6 | 93.51 |
|  |  |  | 12 | 109.67 | 44.33 | 122.4 | 23.55 | 89.42 |
|  |  |  | 13 | 102.88 | 54.24 | 112.72 | 36.31 | 72.31 |
|  |  |  | 14 | 102.25 | 40.01 | 129.07 | 35 | 99.35 |
|  |  |  | 15 | 227.78 | 82.3 | 154.45 | 23.71 | 87.10 |
|  |  |  | 16 | 307.25 | 104.19 | 140.49 | 42.98 | 84.73 |
|  |  |  | 17 | 324.5 | 55.91 | 120.36 | 80.65 | 97.17 |
|  |  |  | 18 | 148.32 | 83.34 | 97.25 | 27.58 | 79.23 |
|  |  |  | 19 | 379.77 | 155.32 | 126.89 | 72.35 | 87.36 |
|  |  |  | 20 | 446.45 | 175.12 | 128.71 | 89.07 | 82.61 |
|  |  |  | 21 | 369.39 | 209 | 103.52 | 88.96 | 69.71 |
|  |  |  | 22 | 117.35 | 46.147 | 95.97 | 62.84 | 87.83 |
|  |  |  | 23 | 147.32 | 84.07 | 92.04 | 61.76 | 88.67 |
|  |  |  | 24 | 619.02 | 332.4 | 110.33 | 85.85 | 99.76 |
|  |  |  | 25 | 396.95 | 158.46 | 107.34 | 93.68 | 74.71 |
|  |  |  | 26 | 481.14 | 375.38 | 97.01 | 58.24 | 87.04 |
|  |  |  | 27 | 198.54 | 76.38 | 114.78 | 46.66 | 88.87 |
|  |  |  | 28 | 372.92 | 224.9 | 121.18 | 58.73 | 91.59 |
|  |  |  | 29 | 304 | 211.7 | 139.23 | 20.45 | 98.54 |
|  |  |  | 30 | 332.17 | 157.17 | 118.78 | 55.17 | 91.24 |
|  |  |  | 31 | 322.85 | 102.23 | 116.6 | 66.27 | 94.07 |
|  |  |  | 32 | 463.3 | 124.25 | 139.38 | 76.67 | 88.23 |
|  |  |  | 33 | 367.75 | 100.42 | 131.88 | 66.65 | 89.49 |
|  |  |  | 34 | 405.61 | 201.19 | 104.07 | 90.38 | 82.81 |
|  |  |  | 35 | 260.1 | 67.66 | 97.96 | 88.81 | 97.19 |
|  |  |  | 36 | 476.68 | 193.72 | 120.5 | 85.09 | 81.39 |
| *Cervus elaphus* | M50045 | Mandible | 1 | 172.85 | 61.66 | 116.82 | 40.08 | 93.91 |
| = |  |  | 2 | 162.32 | 39.88 | 117.5 | 32.69 | 88.73 |
| *Cervus elaphus* | M49979 | Mandible | 1 | 298.02 | 123.59 | 106.63 | 79.35 | 72.90 |
| *Canis lupus* | M13794 | Mandible | 1 | 896.23 | 94.26 | 164.51 | 57.51 | 88.01 |
|  |  |  | 2 | 204.61 | 153.26 | 155.83 | 12.75 | 88.66 |
|  |  |  | 3 | 315.6 | 62.14 | 174.13 | 13.03 | 90.85 |
|  |  |  | 4 | 241.76 | 66.29 | 171.53 | 12.5 | 90.28 |
|  |  |  | 5 | 398.5 | 54.41 | 150.09 | 51.15 | 89.75 |
| *Lepus timidus* | M13806 | Femur (diaphysis) | 1 | 268.6 | 87.52 | 134.17 | 37.7 | 95.22 |
|  |  | = | 2 | 98.87 | 46.19 | 119.49 | 28.88 | 108.75 |
|  |  | = | 3 | 238.22 | 82.72 | 78.41 | 69.86 | 106.50 |
|  |  | = | 4 | 188.13 | 42.62 | 114.08 | 49.59 | 86.33 |
| *Cignus musicus* | A1899 | humerus | 1 | 270.04 | 44.75 | 127.06 | 46.95 | 101.92 |
|  |  |  | 2 | 104.12 | 16.56 | 154.74 | 11.78 | 92.1 |
|  |  |  | 3 | 200.27 | 128.1 | 137.01 | 17.74 | 84.31 |
|  |  |  | 4 | 216.04 | 69.69 | 144.34 | 21.16 | 81.97 |
|  |  |  | 5 | 173.46 | 119.31 | 137.74 | 12.16 | 82.65 |
|  |  |  | 6 | 126.39 | 77.84 | 141.4 | 10.58 | 82.75 |
|  |  |  | 7 | 294.68 | 104.4 | 142.31 | 41.75 | 86.46 |
|  |  |  | 8 | 241.63 | 106.22 | 128.46 | 39.36 | 99.39 |
|  |  |  | 9 | 151.92 | 117.15 | 125.27 | 12.84 | 99.34 |
|  |  |  | 10 | 240.97 | 45.6 | 165.54 | 16.43 | 90.49 |
|  |  |  | 11 | 162.02 | 86.44 | 122 | 18.45 | 107.63 |
|  |  |  | 12 | 175.45 | 68.56 | 128.25 | 19.79 | 106.13 |
|  |  |  | 13 | 152.38 | 43.59 | 145.37 | 17.19 | 82.71 |
|  |  |  | 14 | 245.28 | 156.76 | 146.8 | 11.6 | 81.59 |
|  |  |  | 15 | 123.97 | 19.06 | 162.81 | 9.69 | 90.30 |
|  |  |  | 16 | 194.85 | 132.41 | 136.89 | 15.26 | 96.87 |
|  |  |  | 17 | 267.43 | 61.66 | 117.14 | 54.4 | 104.98 |
|  |  |  | 18 | 215.68 | 137.7 | 156.05 | 12.11 | 82.63 |
|  |  |  | 19 | 129.57 | 39 | 119 | 17.37 | 70.76 |
|  |  |  | 20 | 237.77 | 37.99 | 164.39 | 18.1 | 90.76 |
|  |  |  | 21 | 323.46 | 61.95 | 147.5 | 37.84 | 85.86 |
|  |  |  | 22 | 295.38 | 104.58 | 148.09 | 28.29 | 92.78 |
|  |  |  | 23 | 291.37 | 68.68 | 157.02 | 25.09 | 89.99 |
|  |  |  | 24 | 468.09 | 126.64 | 160.51 | 41.36 | 88.60 |
|  |  |  | 25 | 65.86 | 37.48 | 105.9 | 13.51 | 100.44 |
|  |  |  | 26 | 183.07 | 45.84 | 147.28 | 17.5 | 82.65 |
| *Cignus musicus* | A1903 | ulna | 1 | 432.9 | 86.17 | 137.15 | 71.33 | 86.10 |
|  |  |  | 2 | 525.93 | 61.02 | 168.65 | 42.11 | 89.50 |
|  |  |  | 3 | 425.82 | 119.08 | 151.55 | 45.25 | 84.24 |
|  |  |  | 4 | 295.3 | 76.01 | 114.73 | 77.31 | 91.84 |
|  |  |  | 5 | 755.04 | 124.88 | 148.49 | 55.01 | 98.46 |
|  |  |  | 6 | 204.49 | 36.63 | 122.01 | 44 | 84.35 |
|  |  |  | 7 | 146.55 | 39.58 | 120.79 | 31.57 | 86.92 |
|  |  |  | 8 | 121.36 | 36.85 | 132.7 | 20.28 | 88.05 |
| *Lagopus mutus* | A1906 | Humerus | 1 | 168.6 | 18.19 | 145.6 | 20.71 | 88.41 |
|  |  |  | 2 | 131.13 | 24.46 | 150.24 | 14.18 | 95.05 |
|  |  |  | 3 | 178.95 | 110.2 | 153.37 | 9.75 | 84.01 |
|  |  |  | 4 | 141.39 | 45.11 | 131.79 | 16.73 | 87.71 |
|  |  |  | 5 | 162.77 | 86.93 | 152.66 | 11.5 | 91.62 |
|  |  |  | 6 | 78.57 | 43.77 | 135.92 | 7.05 | 91.42 |
|  |  |  | 7 | 105.51 | 50.64 | 144.96 | 10.78 | 92.76 |
|  |  |  | 8 | 273.36 | 148.39 | 130.3 | 37.96 | 90.82 |
|  |  |  | 9 | 200.74 | 73.07 | 142.98 | 25.69 | 85.92 |
|  |  |  | 10 | 254.17 | 152.19 | 140.73 | 24.72 | 89.18 |
|  |  |  | 11 | 272.93 | 127.27 | 125.31 | 41.69 | 96.55 |
|  |  |  | 12 | 169.33 | 44.91 | 138.51 | 41.52 | 69.25 |
|  |  |  | 13 | 173.47 | 47.32 | 107.1 | 45.94 | 112.16 |
|  |  |  | 14 | 190.72 | 61.57 | 94.93 | 63.84 | 93.57 |
|  |  |  | 15 | 157.8 | 50.33 | 150.79 | 24.94 | 84.53 |
|  |  |  | 16 | 100.45 | 21.85 | 143.67 | 22.3 | 93.39 |
|  |  |  | 17 | 183.72 | 66.57 | 138.92 | 24.39 | 86.01 |
|  |  |  | 18 | 232.87 | 149.65 | 144.44 | 20.01 | 92.34 |
|  |  |  | 19 | 256.71 | 30.46 | 146.13 | 34.6 | 85.94 |
| *Gallus sp* | A1917 | Humerus | 1 | 498.11 | 128.83 | 136.48 | 85.92 | 90.7 |
|  |  |  | 2 | 173.65 | 53.98 | 125.14 | 37.13 | 83.68 |
|  |  |  | 3 | 303.03 | 70.41 | 139.92 | 54.74 | 91.34 |
|  |  |  | 4 | 220.47 | 65.56 | 153.42 | 33.68 | 89.32 |
|  |  |  | 5 | 271.78 | 78.11 | 153.6 | 32.17 | 95.04 |
|  |  |  | 6 | 369.31 | 56.7 | 141.42 | 38.74 | 99.71 |
|  |  |  | 7 | 385.55 | 53.93 | 138.52 | 46.62 | 101.77 |
|  |  |  | 8 | 320.47 | 150.74 | 155.62 | 22.68 | 95.5 |
|  |  |  | 9 | 183.47 | 72.71 | 127.84 | 29.51 | 87.02 |
