# Supplementary material for: An Upper Palaeolithic engraved human bone associated with ritualistic cannibalism
Source: PLoS One. 2017 Aug 9;12(8):e0182127. doi: 10.1371/journal.pone.0182127 (PMC5549908; doi:10.1371/journal.pone.0182127)
Supplement: S2 File — (DOCX) [file pone.0182127.s002.docx]

**Supplementary Information 2**

3-Dimensional Alicona image, Alicona profile (with and without measurements) and description of each engraving mark on the human radius (M54074).

**Engraving 1**

| 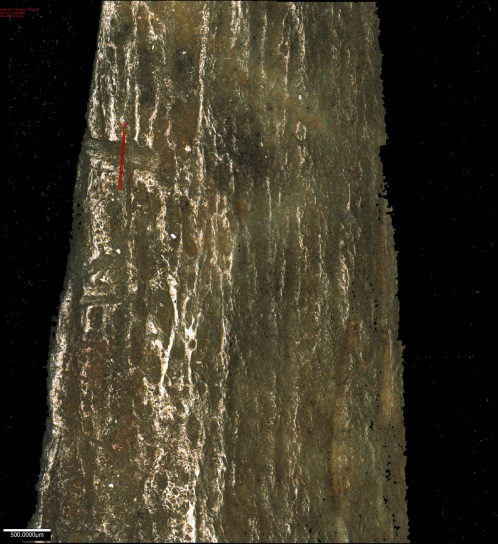 | 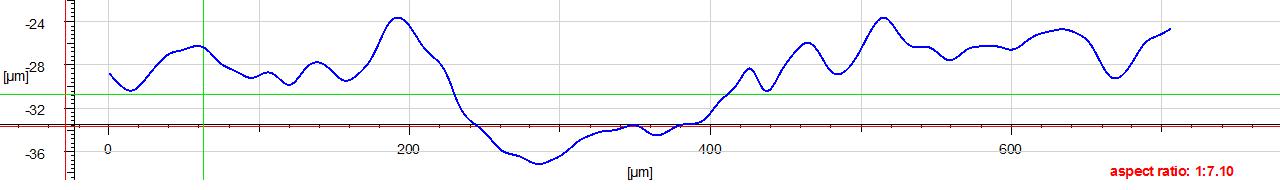  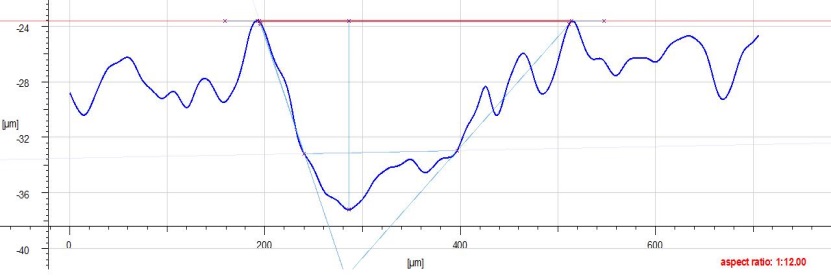 |
| --- | --- |

Figure SI1-1: a) 3D Alicona image of the incised surface of the human radius. The red line indicates where the profile measurements were taken; b-c) profiles of the incision with and without measurements.

This is a single-stroke incision produced by a slicing action of a one-side retouched blade (Figure SI1b: the profile indicates a smooth left side of the profile and a rougher right side, which is the retouched side of the tool).

The incision is ~1.2 mm long. The values of the profile measurements, taken at the incision’s midpoint (Figure SI1a and c), are:

WIS = 322.45 µm

WIB = 156.98 µm

OA = 163.51˚

D = 13.7 µm

ATI = 86.35˚

**Engraving 2**

| 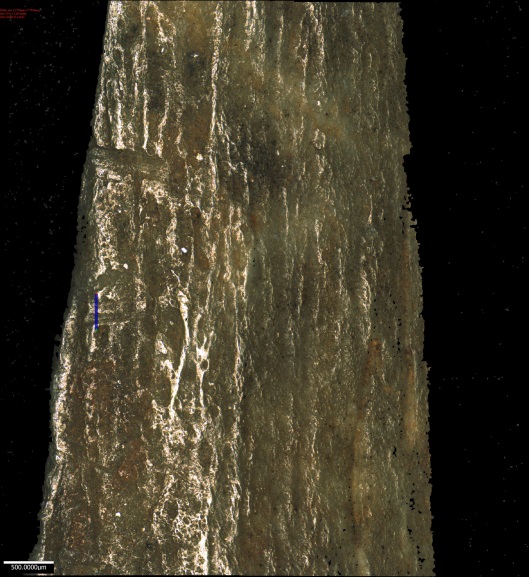 | 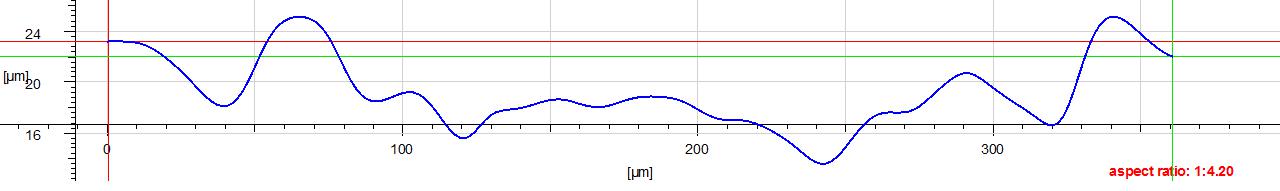  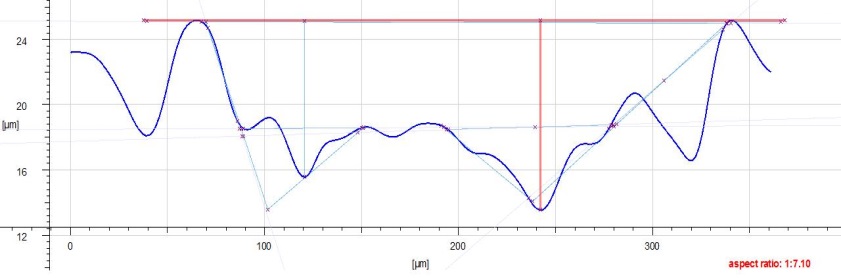 |
| --- | --- |

Figure SI1-2: a) 3D Alicona image of the incised surface of the human radius. The blue line indicates where the profile measurements were taken; b-c) profiles of the incision with and without measurements.

This is a double incision produced by a to-and-fro sawing action. Both incisions are likely to have been produced by a retouched blade (Figure SI2b: the profile shows an irregular profile on both sides of the double incisions).

We divided incision 2 into sub-incisions A (toward the proximal epiphysis) and B (toward distal epiphysis). The length of sub-incision A is 0.94 mm and sub-incision B is ~1.0 mm. The overall WIS value is 271.53 µm. The values of the profile measurements, taken at the edge of the fracture (the incision has been cut short by the fracture; Figure SI2a and c), are respectively:

WIB = 61.04 µm and 84.01 µm

OA = 154.24˚ and 168.06˚

D = 9.64 µm and 11.72 µm

ATI = 82.16˚ and 89.94˚

**Engraving 3**

| 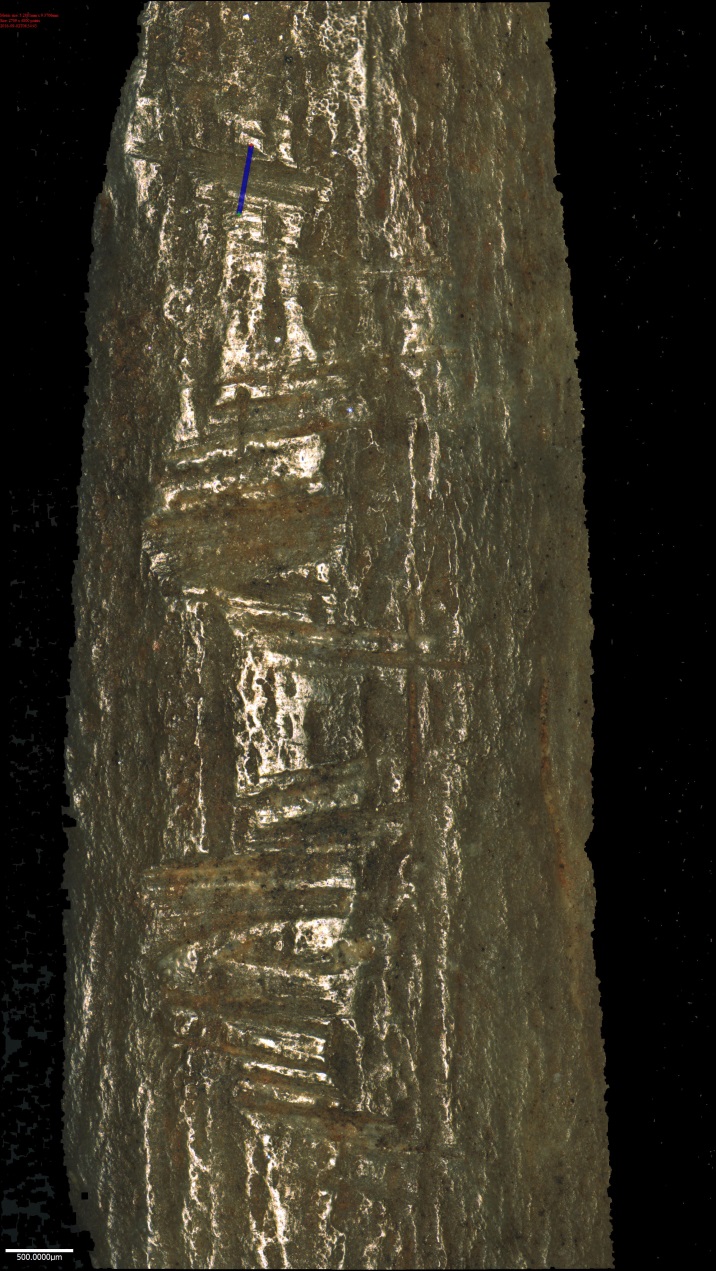 | 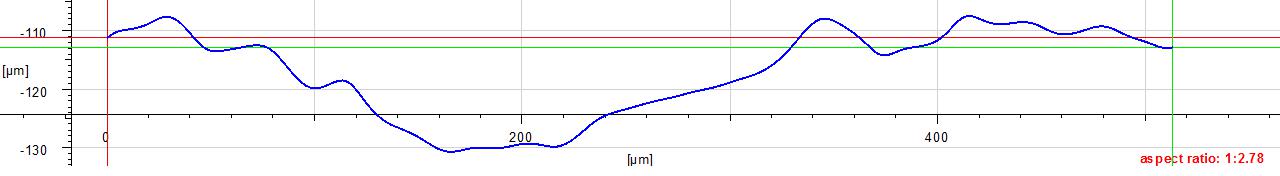  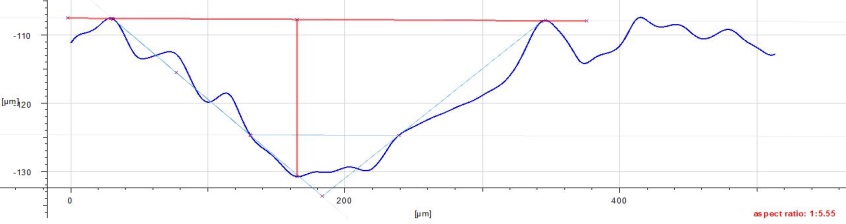 |
| --- | --- |

Figure SI1-3: a) 3D Alicona image of the incised surface of the human radius. The blue line indicates where the profile measurements were taken; b-c) profiles of the incision with and without measurements.

This is a double incision produced by a to-and-fro slicing action. However, the two slicing cuts overlap almost completely, which prohibits the recognition of sub-incisions (*sensu* Bello et al., 2013). This overlapping results in a wider WIB, mimicking a U-shape across the entire incision (Figure SI1-3a). Both incisions are likely to have been produced by a unilaterally retouched blade.

The full length of the incision is 1.59 mm. The values of the profile measurements, taken at their midpoint, are:

WIS = 317.24 µm

WIB = 108.25 µm

OA = 161.17˚

D = 23.18 µm

ATI = 89.70˚

**Engraving 4**

| 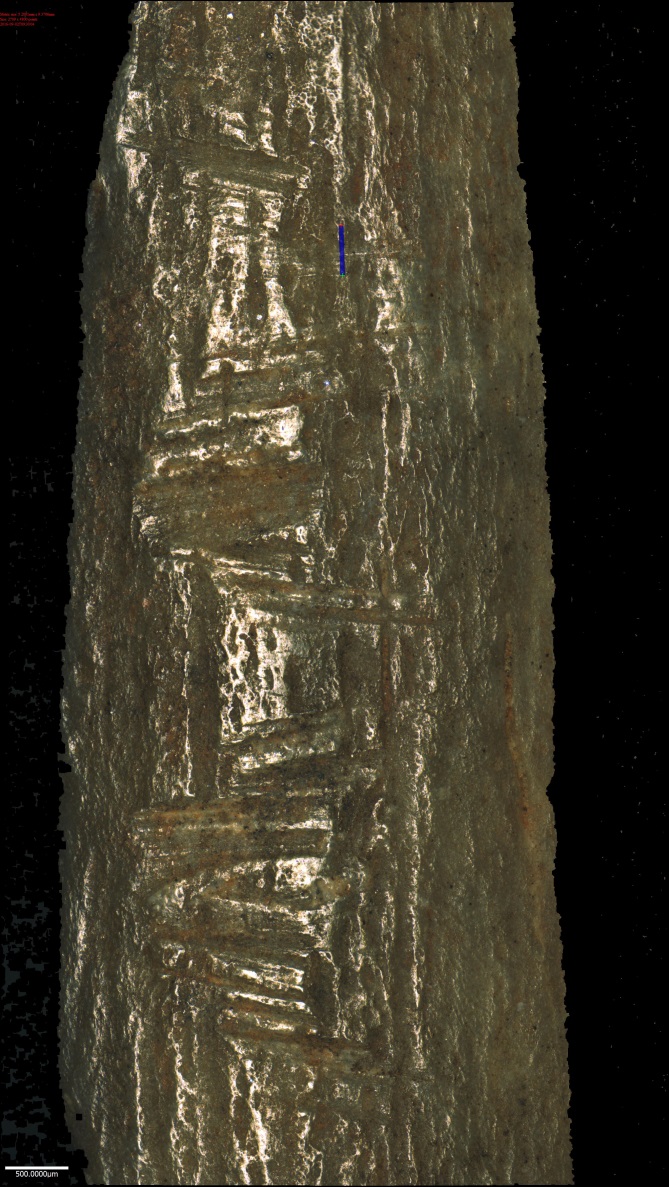 | 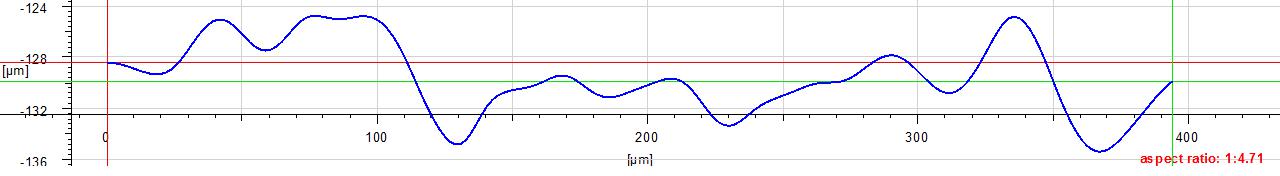  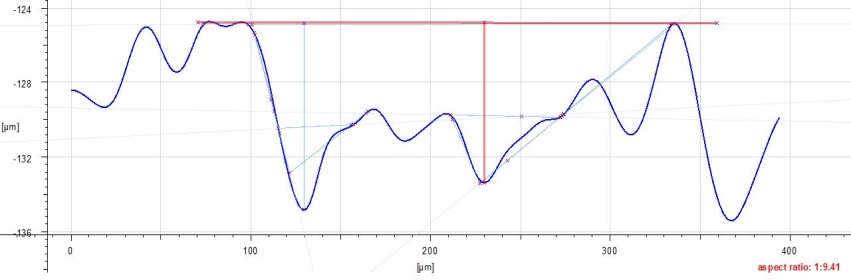 |
| --- | --- |

Figure SI1-4: a) 3D Alicona image of the incised surface of the human radius. The blue line indicates where the profile measurements were taken; b-c) profiles of the incision with and without measurements.

This is a double incision produced by a to-and-fro sawing action. There is no complete overlapping of the two incisions and both incisions are likely to have been produced by a single-side retouched blade.

We divided incision 2 into sub-incisions A (toward the proximal epiphysis) and B (toward distal epiphysis). The total width of the double incision is 236.62 µm, sub-incision A is 1.5 mm long, sub-incision B is 1.4 mm long. The values of the profile measurements, taken at the incision’s midpoint, are:

WIB = 41.89 µm and 61.45 µm

OA = 154.80˚ and 162.82 ˚

D = 10.07 µm and 8.63 µm

ATI = 81.29˚ and 85.92 ˚

**Engraving 5**

| 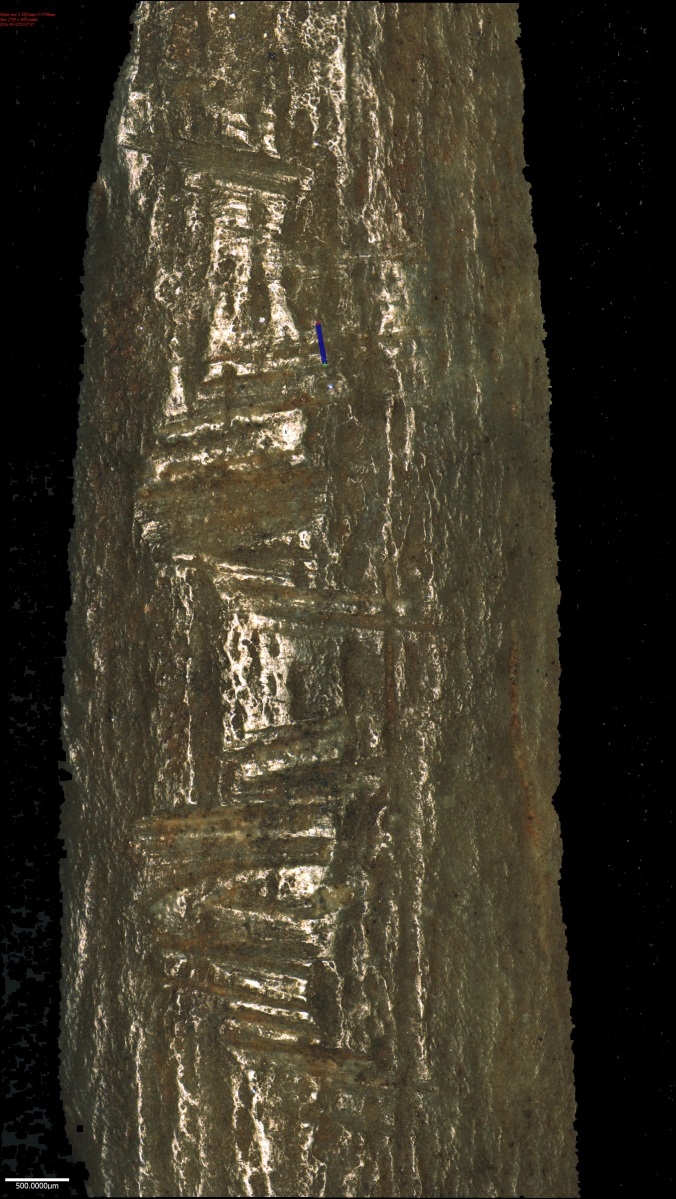 | 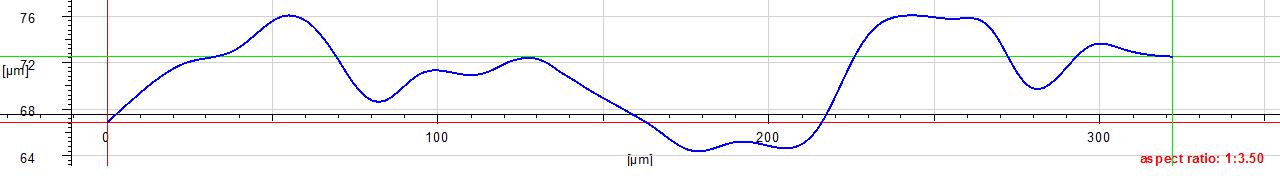  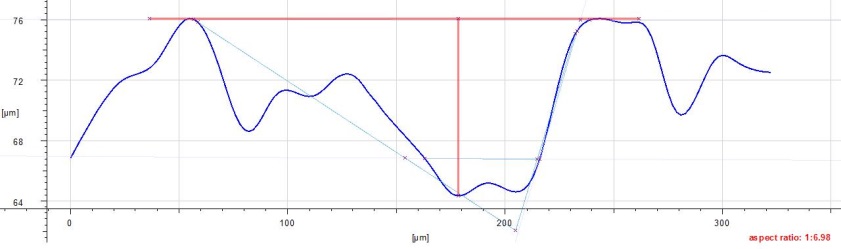 |
| --- | --- |

Figure SI1-5: a) 3D Alicona image of the incised surface of the human radius. The blue line indicates where the profile measurements were taken; b-c) profiles of the incision with and without measurements.

This is a single-stroke incision with a clear shoulder effect toward the proximal epiphysis. The incision was likely to have been produced by a unilaterally retouched tool.

The incision is ~2.21 mm long. The values of the profile measurements, taken at the incision’s midpoint (Figure SI5a and c), are:

WIS = 182.38 µm

WIB = 53.03 µm

OA = 149.11˚

D = 11.79 µm

ATI = 100.79˚

**Engraving 6**

| 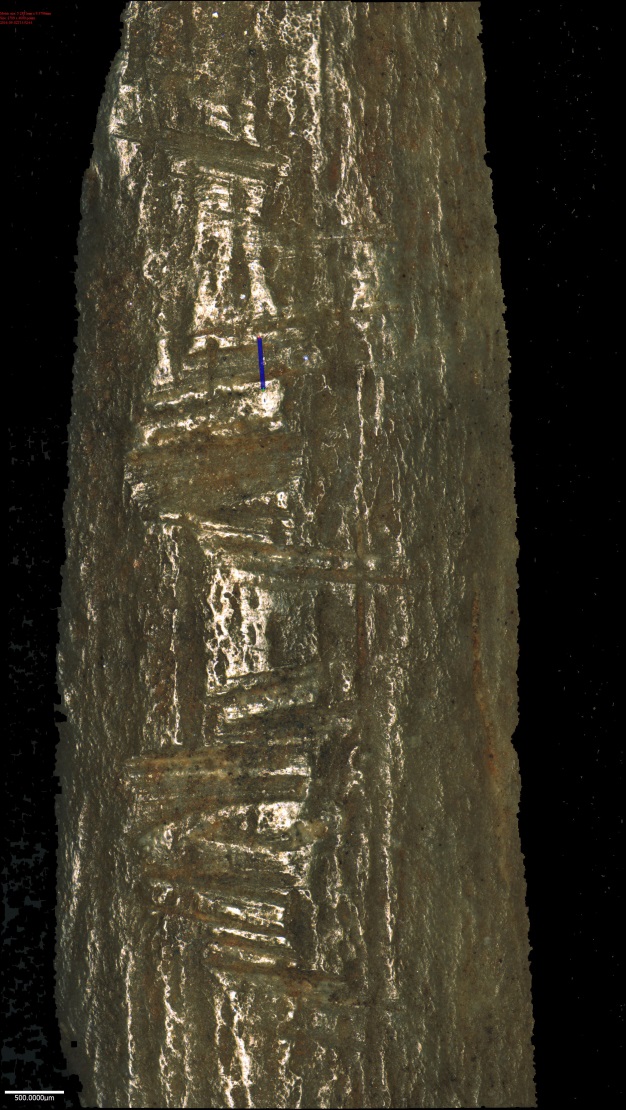 | 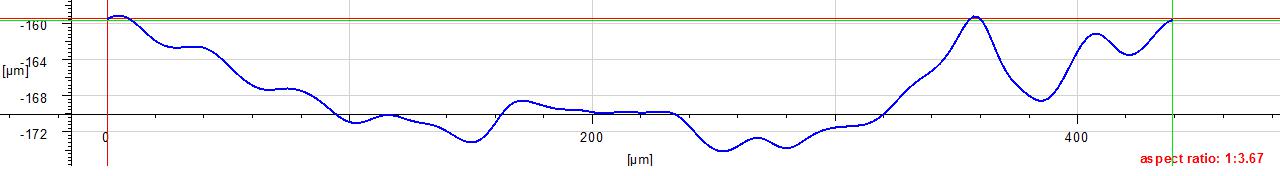  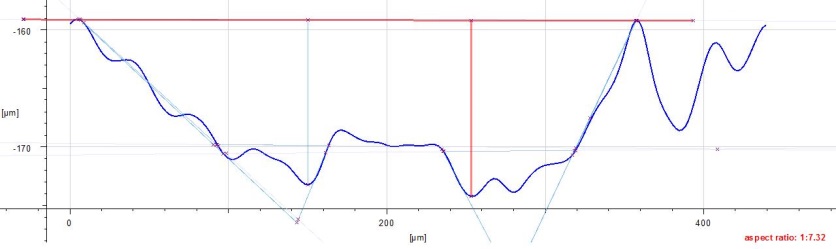 |
| --- | --- |

Figure SI1-6: a) 3D Alicona image of the incised surface of the human radius. The blue line indicates where the profile measurements were taken; b-c) profiles of the incision with and without measurements.

This is a double incision produced by a to-and-fro slicing action. The first cut (toward the distal end) starts ~0.3 mm before the previous one. Despite this asymmetry, the two sub-incisions overlap almost perfectly. Both sub-incisions are likely to have been produced by a unilaterally retouched blade.

We divided incision 2 into sub-incision A (toward the proximal epiphysis) and sub-incision B (toward distal epiphysis). The total width of the double incision is 352.95 µm, while sub-incision A is 1.91 mm long and sub-incision B is 2.45 mm long. The values of the profile measurements, taken at the incision’s midpoint, are:

WIB = 70.24 µm and 83.31 µm

OA = 154.39˚ and 149.46 ˚

D = 14.15 µm and 15.11 µm

ATI = 95.70˚ and 90.66 ˚

**Engraving 7**

| 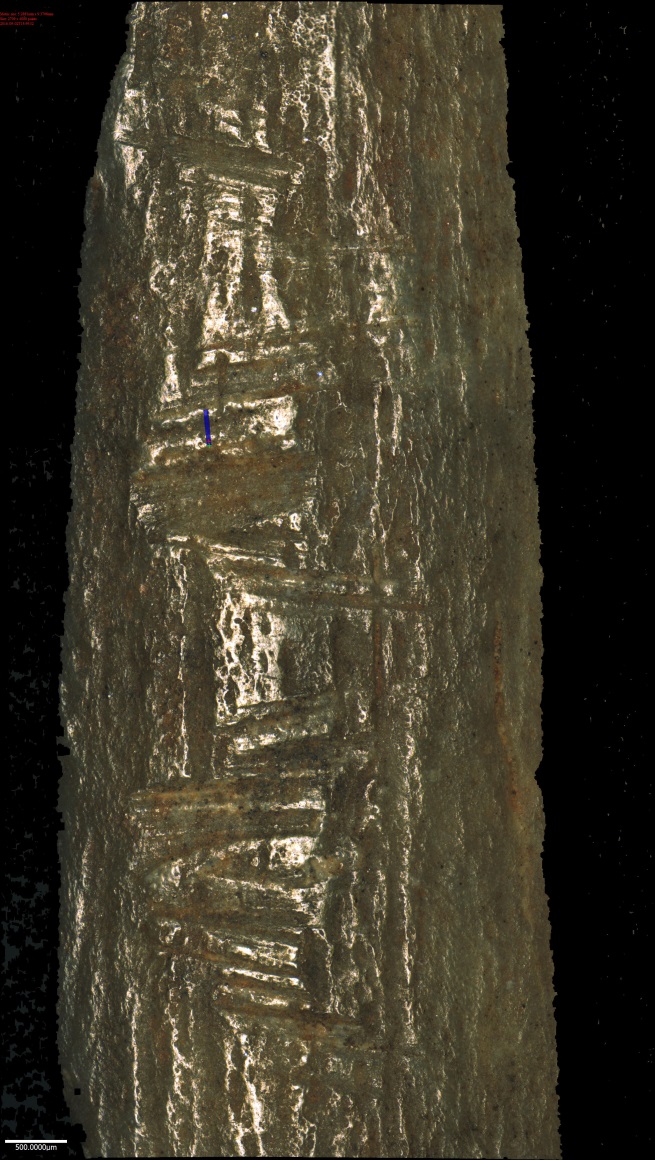 | 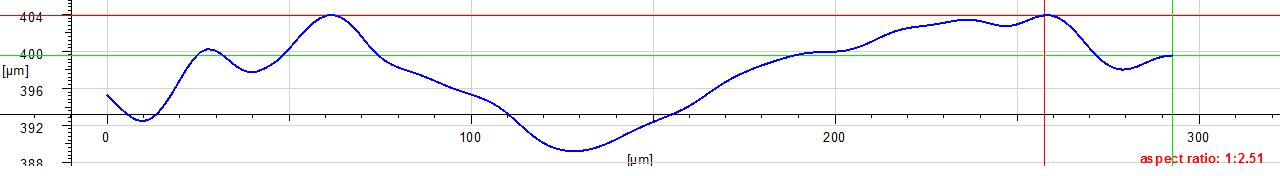  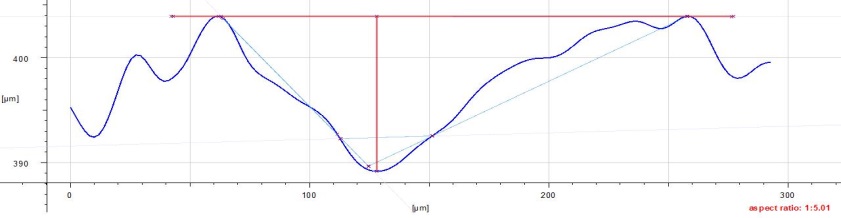 |
| --- | --- |

Figure SI1-7: a) 3D Alicona image of the incised surface of the human radius. The blue line indicates where the profile measurements were taken; b-c) profiles of the incision with and without measurements.

This is a single-stroke incision probably produced by a unilaterally retouched blade.

The full length of the incision is 1.27 mm. The values of the profile measurements, taken at the incision midpoint, are:

WIS = 196.28 µm

WIB = 38.48 µm

OA = 160.78˚

D = 14.86 µm

ATI = 86.5˚

**Engraving 8**

| 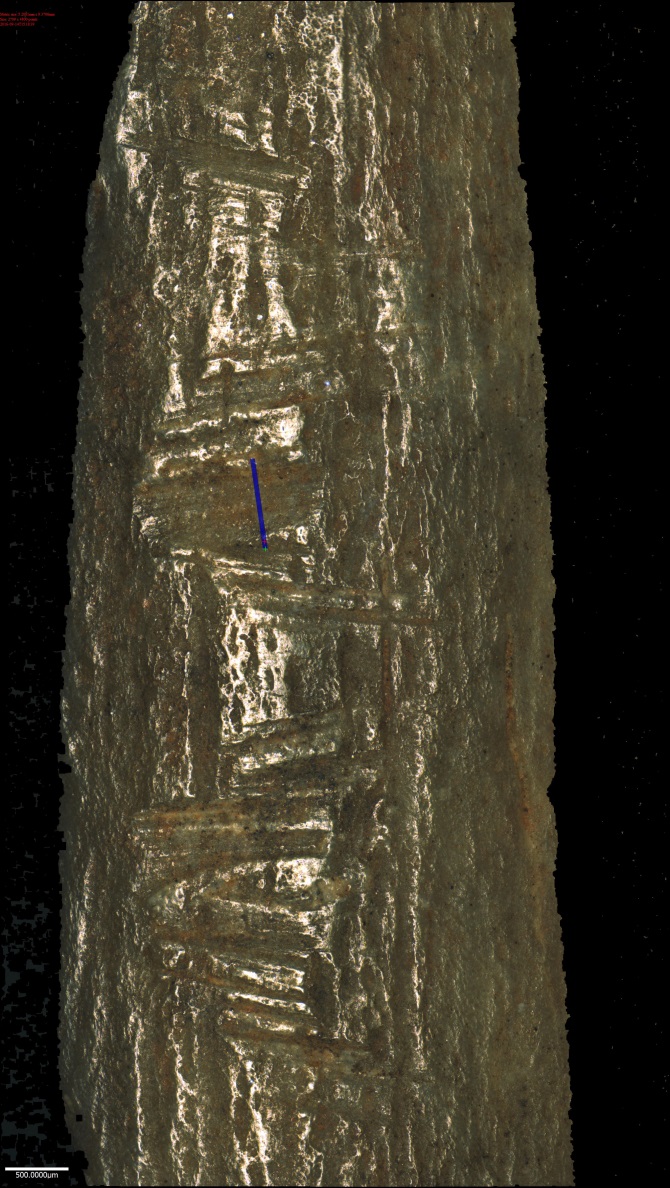 | 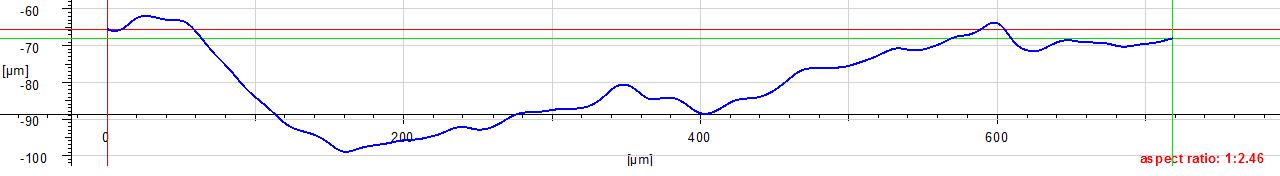  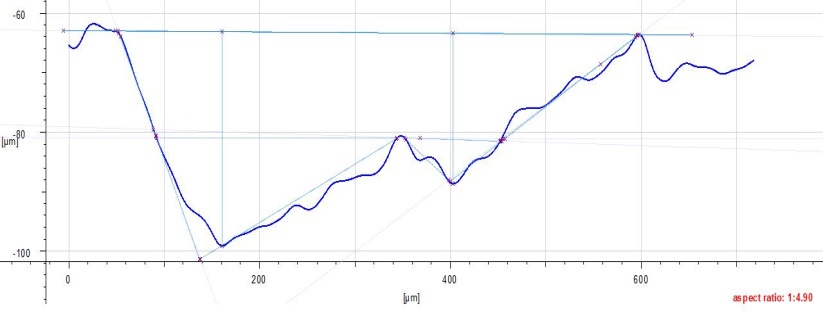 |
| --- | --- |

Figure SI1-8: a) 3D Alicona image of the incised surface of the human radius. The blue line indicates where the profile measurements were taken; b-c) profiles of the incision with and without measurements.

This is a wide incision produced by scraping. The micro-morphological characteristics of each internal sub-incision suggest that the action was interrupted and the tool repositioned to continue the scraping of the bone surface. The profiles of the two sub-incisions are very similar, and their morphology suggests that they were produced by a unilaterally retouched blade.

We divided incision 8 into sub-incisions A (toward the proximal epiphysis) and B (toward distal epiphysis). The total width of the double incision is 548.75 µm, sub-incision A is 1.68 mm long and sub-incision B is 1.55 mm long. The values of the profile measurements, taken at the incision’s midpoint, are:

WIB = 252.33 µm and 100.58 µm

OA = 149.96˚ and 164.20 ˚

D = 36.15 µm and 25.38 µm

ATI = 81.11˚ and 89.09 ˚

**Engraving 9**

| 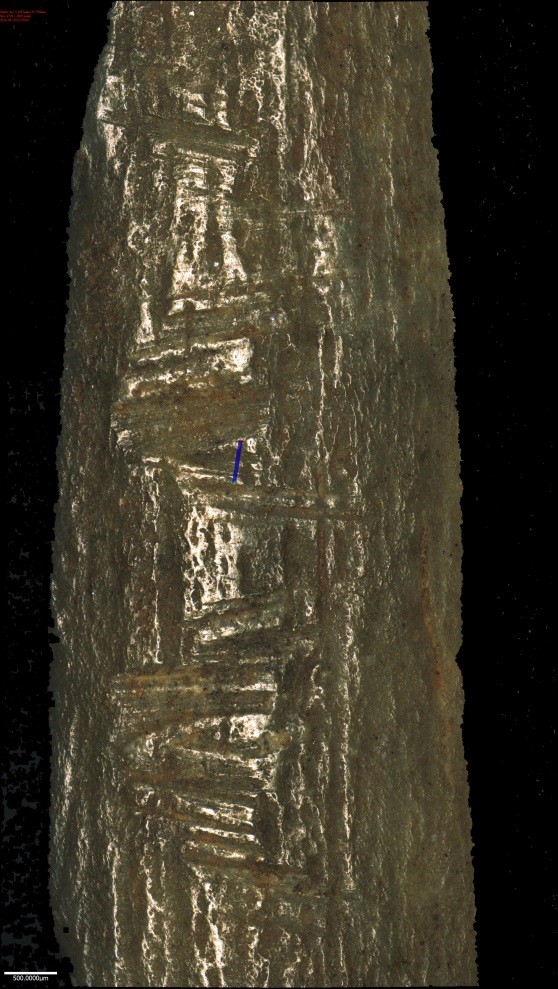 | 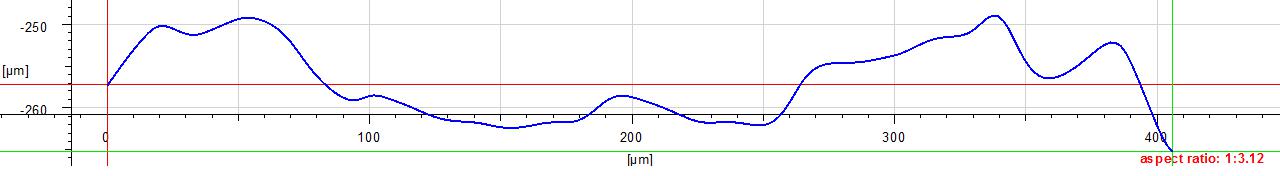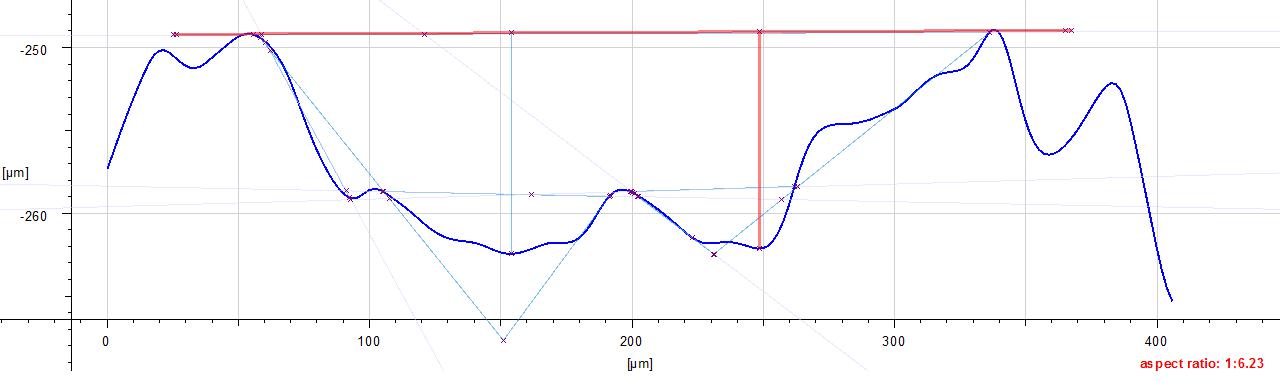 |
| --- | --- |

Figure SI1-9: a) 3D Alicona image of the incised surface of the human radius. The blue line indicates where the profile measurements were taken; b-c) profiles of the incision with and without measurements.

This is a double incision produced by a to-and-fro action. This double incision is bisected by incision 8 at an angle of ~30˚. We divided incision 9 into sub-incisions A (toward the proximal epiphysis) and B (toward the distal epiphysis).The total width of the double incision is 280.83 µm, sub-incision A is 0.82 mm long, sub-incision B is 1.06 mm long. The values of the profile measurements, taken at the incision’s midpoint, are:

WIB = 86.45 µm and 62.49 µm

OA = 156.63˚ and 165.85 ˚

D = 13.43 µm and 13.17 µm

ATI = 85.29˚ and 90.19 ˚

**Engraving 10**

| 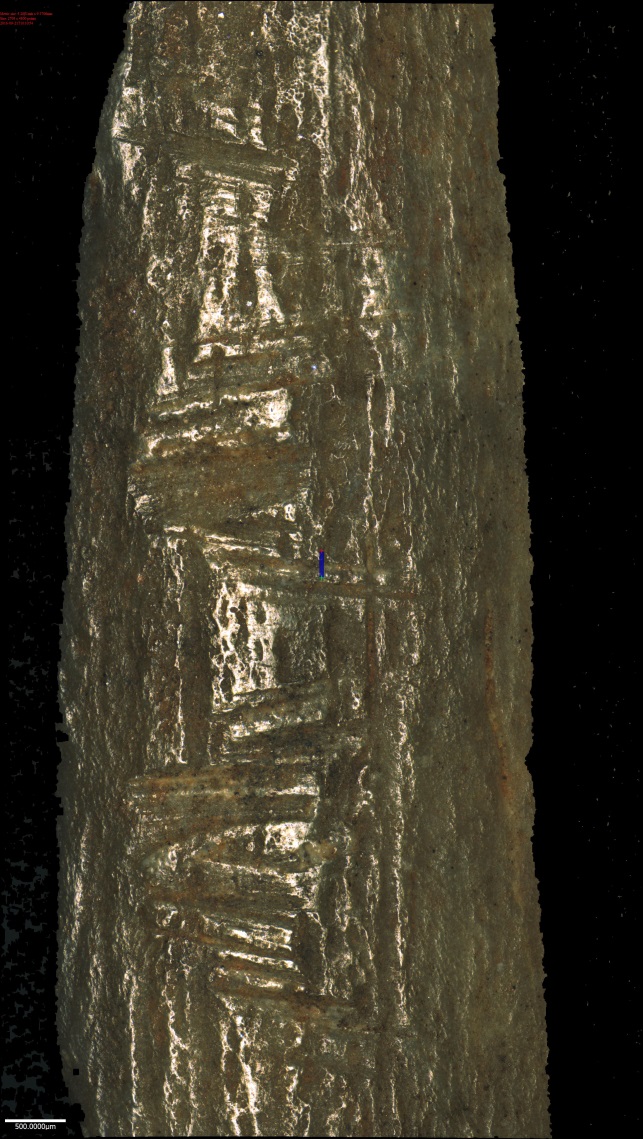 | 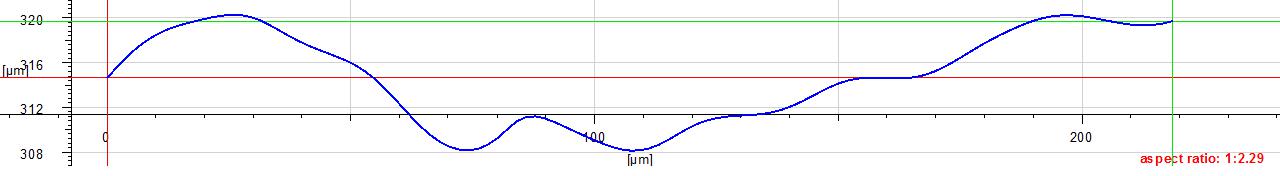  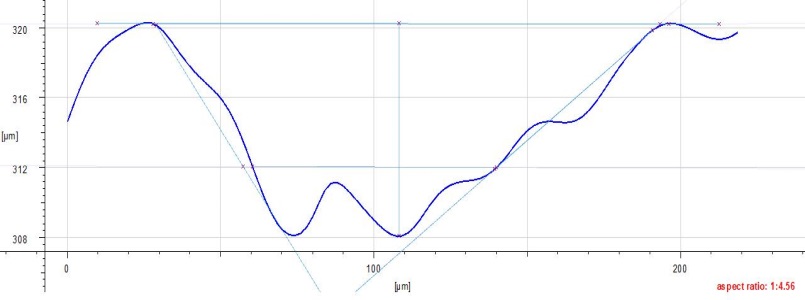 |
| --- | --- |

Figure SI1-10: a) 3D Alicona image of the incised surface of the human radius. The blue line indicates where the profile measurements were taken; b-c) profiles of the incision with and without measurements.

This is a single-stroke incision probably produced by a unilaterally retouched blade.

The full length of the incision is 1.77 mm. The values of the profile measurements, taken at the incision’s midpoint, are:

WIS = 168.31 µm

WIB = 79.67 µm

OA = 155.18˚

D = 12.24 µm

ATI = 86.42˚

**Engraving 11**

| 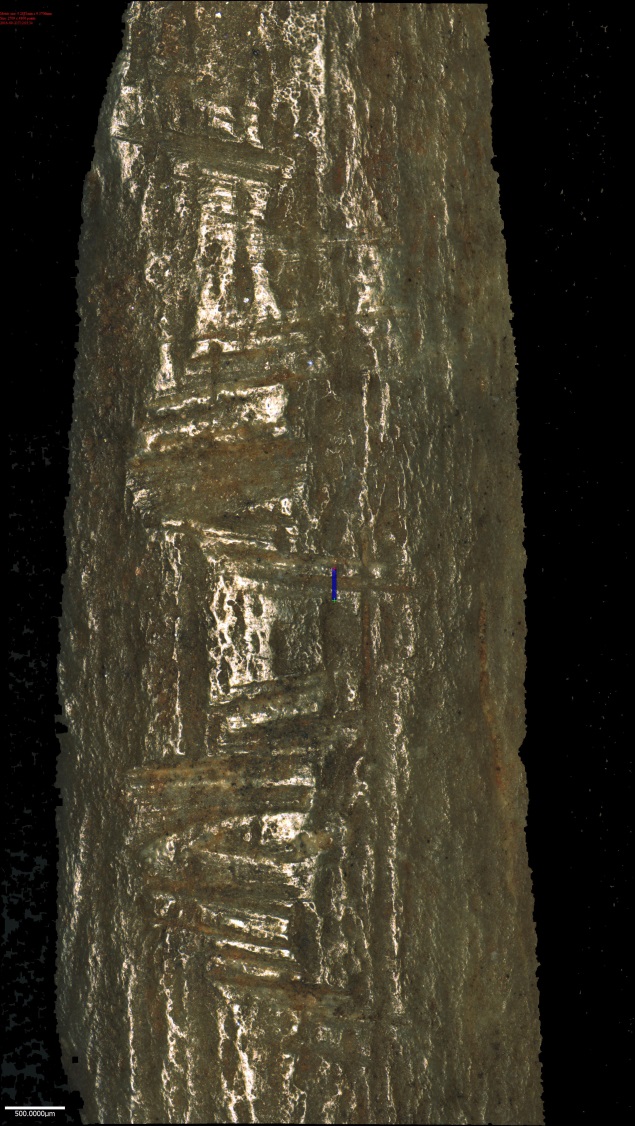 | 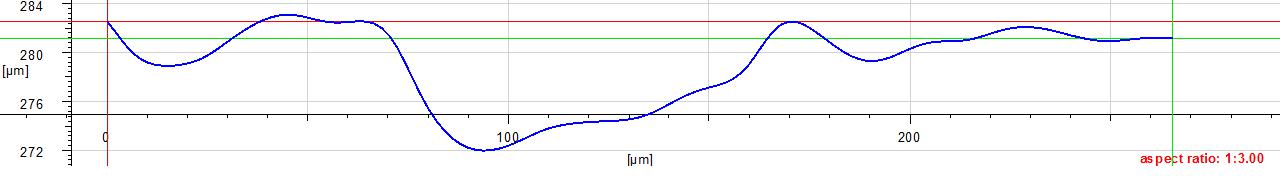  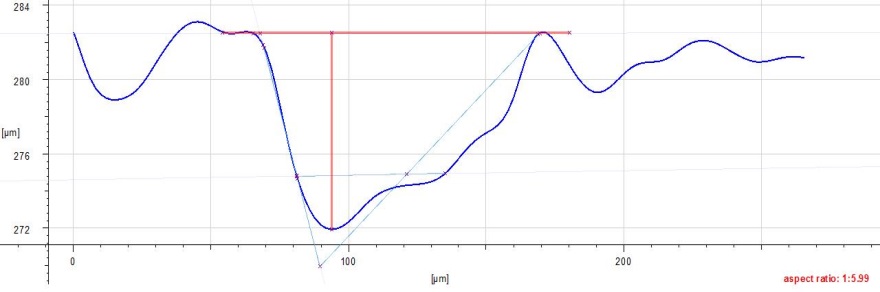 |
| --- | --- |

Figure SI1-11: a) 3D Alicona image of the incised surface of the human radius. The blue line indicates where the profile measurements were taken; b-c) profiles of the incision with and without measurements.

This is a single-stroke incision probably produced by a unilaterally retouched blade. Adjacent to it, towards the distal portion of the diaphysis, one can observe a very short scratch. This is probably a shoulder effect left by irregularities of the stone tool.

The full length of the incision is 1.59 mm. The values of the profile measurements, taken at the incision’s midpoint, are:

WIS = 104.21 µm

WIB = 53.89 µm

OA = 140.65˚

D = 10.67 µm

ATI = 79.78˚

**Engraving 12**

| 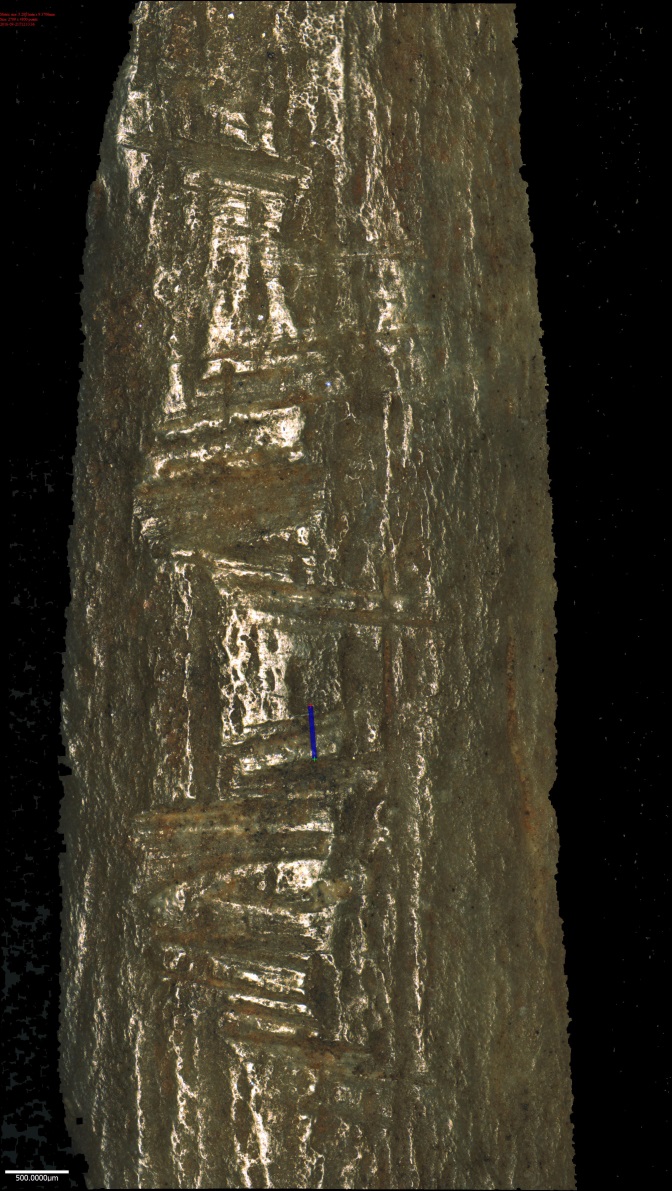 | 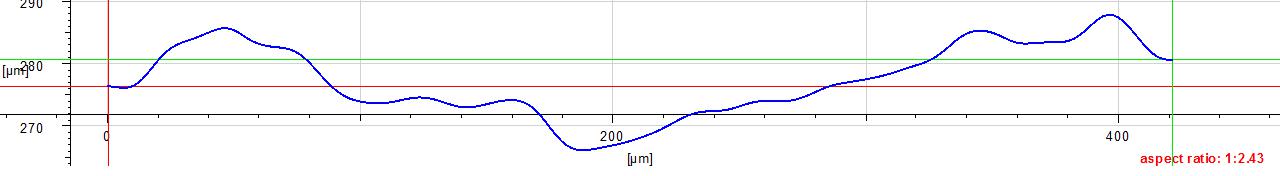  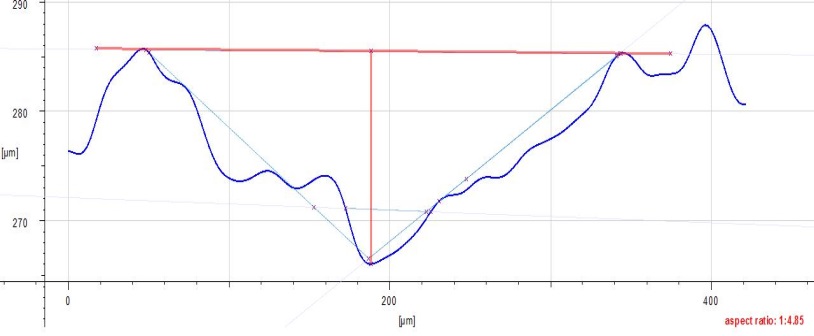 |
| --- | --- |

Figure SI1-12: a) 3D Alicona image of the incised surface of the human radius. The blue line indicates where the profile measurements were taken; b-c) profiles of the incision with and without measurements.

This is a large single incision probably produced by a to-and-fro slicing action with a retouched blade. The two slicing sub-incisions overlap and are not distinguishable. Towards the proximal portion of the diaphysis it is possible to observe a shorter scratch, probably a shoulder effect left by irregularities of the stone tool. The presence of irregularities may have caused the incision to be larger than usual.

The full length of the incision is 1.02 mm. The values of the profile measurements, taken at the incision’s midpoint, are:

WIS = 296.09 µm

WIB = 53.21 µm

OA = 165.26˚

D = 19.60 µm

ATI = 89.50˚

**Engraving 13**

| 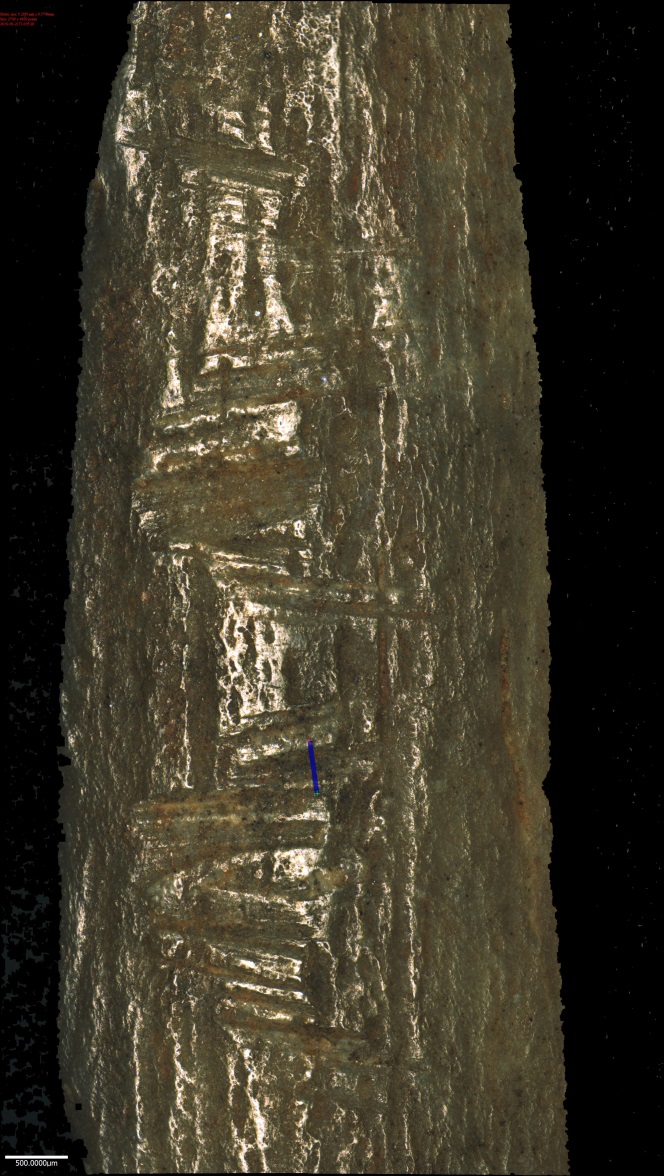 | 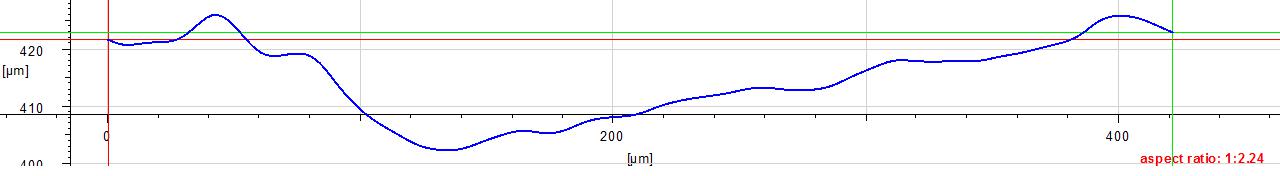  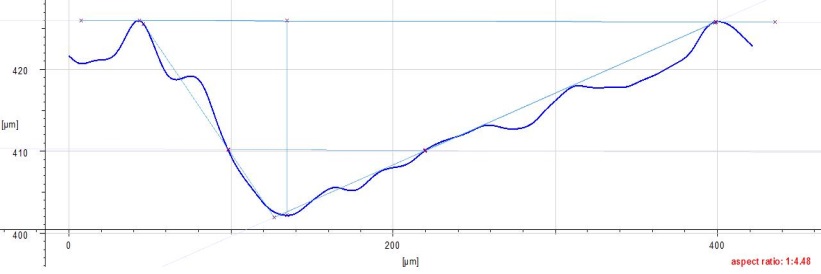 |
| --- | --- |

Figure SI1-13: a) 3D Alicona image of the incised surface of the human radius. The blue line indicates where the profile measurements were taken; b-c) profiles of the incision with and without measurements.

This is a large single incision produced by a to-and-fro action, probably using a unilaterally retouched blade. The internal sub-incisions overlap perfectly giving the impression of a single incision.

The full length of the incision is 1.47 mm. The values of the profile measurements, taken at the incision’s midpoint, are:

WIS = 356.03 µm

WIB = 121.79 µm

OA = 158.56˚

D = 23.93 µm

ATI = 84.3˚

**Engraving 14**

| 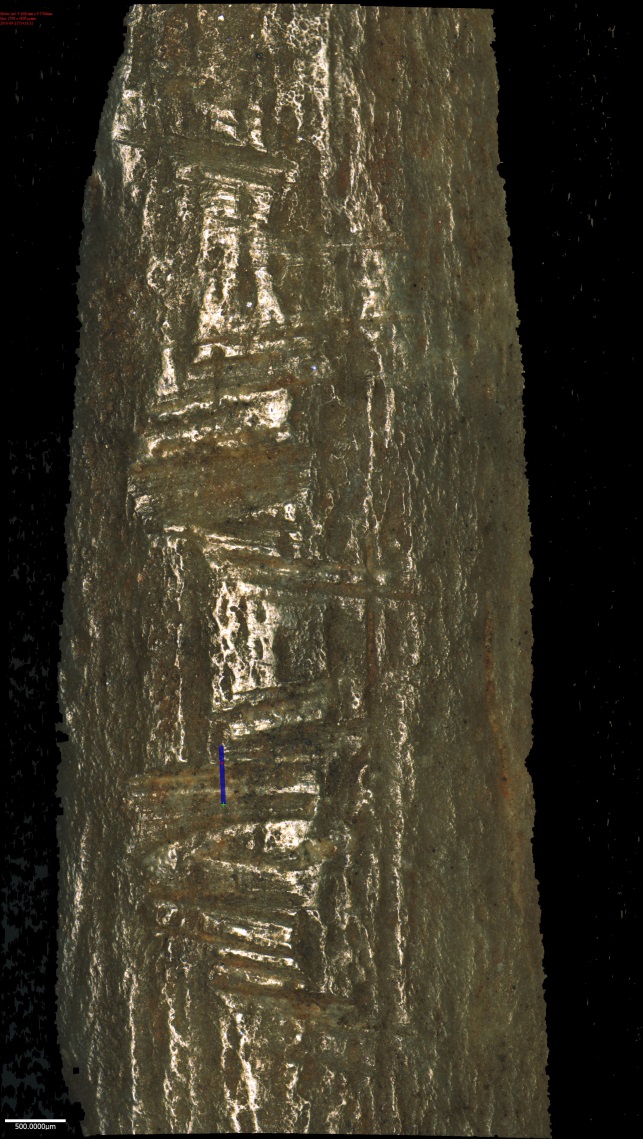 | 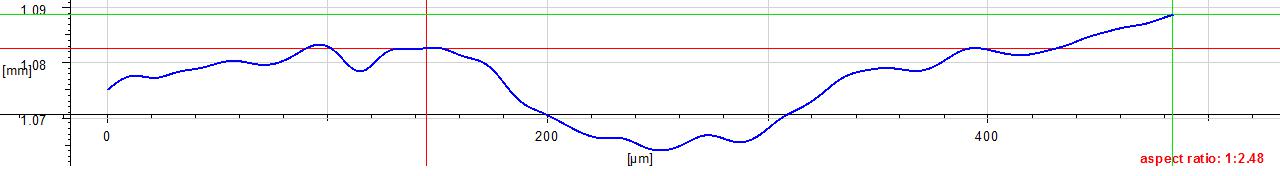  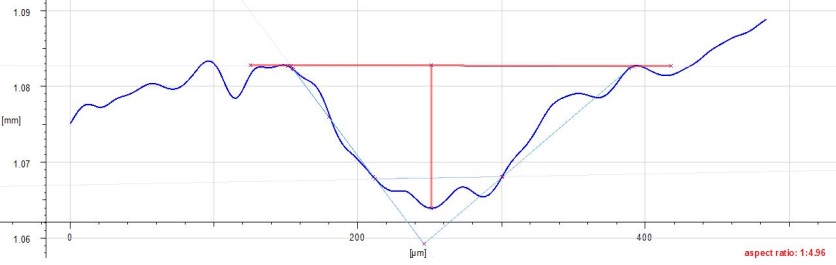 |
| --- | --- |

Figure SI1-14: a) 3D Alicona image of the incised surface of the human radius. The blue line indicates where the profile measurements were taken; b-c) profiles of the incision with and without measurements.

This is a single-stroke incision probably produced by a unilaterally retouched blade.

The full length of the incision is 1.68 mm. The values of the profile measurements, taken at the incision’s midpoint, are:

WIS = 240.75 µm

WIB = 88.34 µm

OA = 156.55˚

D = 18.91 µm

ATI = 87.38˚

**Engraving 15**

| 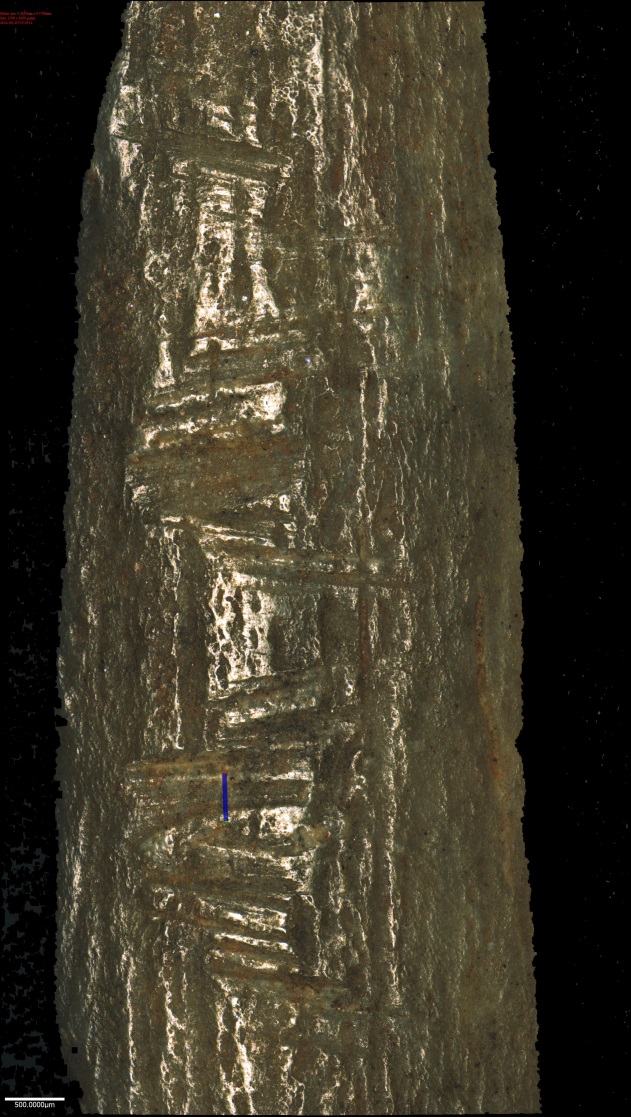 | 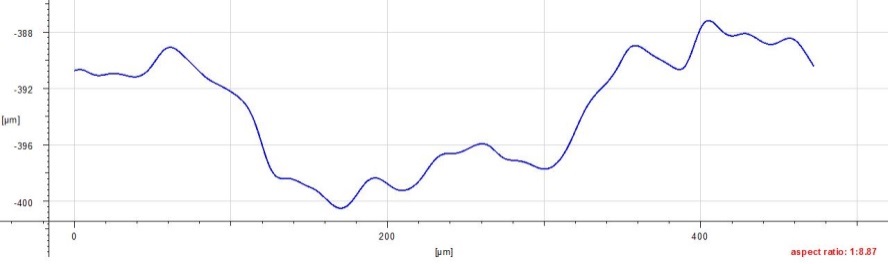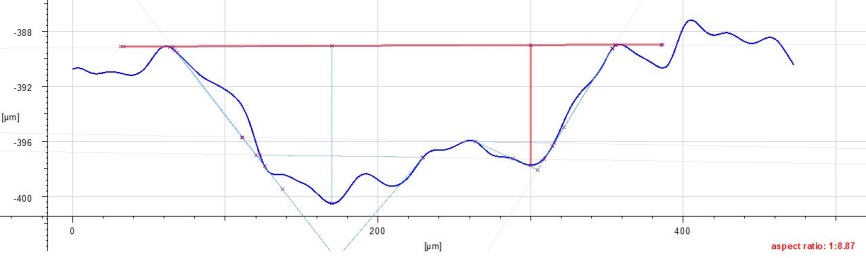 |
| --- | --- |

Figure SI1-15: a) 3D Alicona image of the incised surface of the human radius. The blue line indicates where the profile measurements were taken; b-c) profiles of the incision with and without measurements.

This is a double incision produced by a to-and-fro sawing action. Although the start and end points of the sub-incisions do not overlap perfectly, the midpoint of the double incision is a perfect overlap. We divided incision 15 into sub-incisions A (toward the proximal epiphysis) and B (toward the distal epiphysis). The total width of the double incision is 294.25 µm, sub-incision A is 1.54 mm long and sub-incision B is 1.56 mm long. The values of the profile measurements, taken at the incision’s midpoint, are:

WIB = 102.57 µm and 48.31 µm

OA = 162.73˚ and 166.39 ˚

D = 11.99 µm and 9.04 µm

ATI = 90.90˚ and 93.70 ˚

**Engraving 16**

| 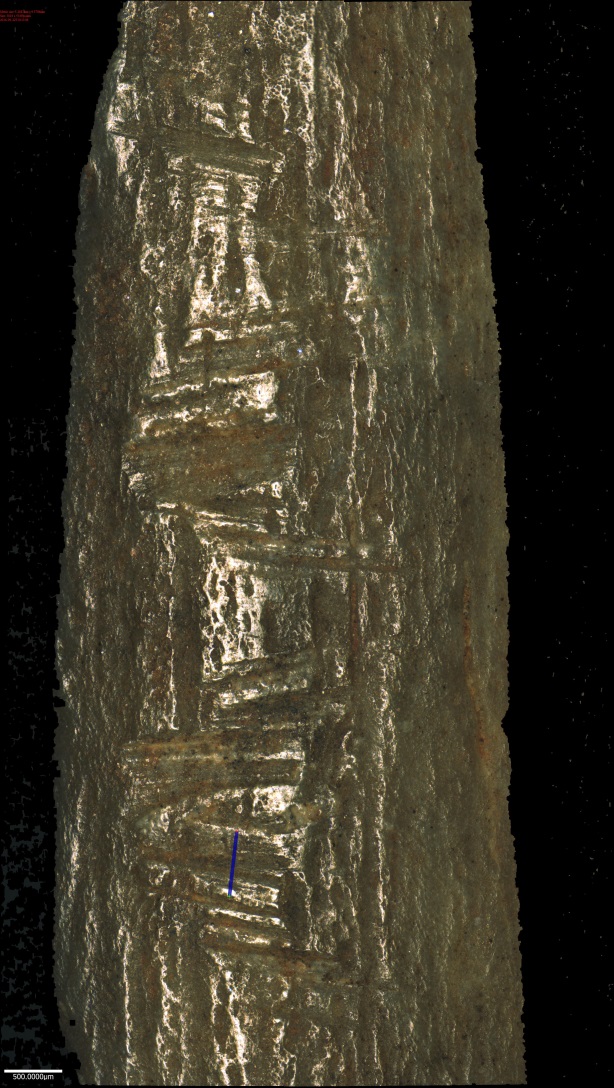 | 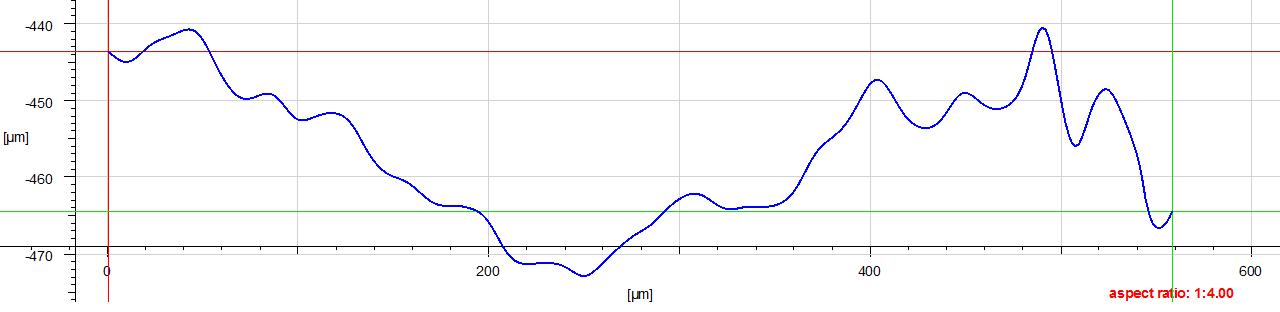  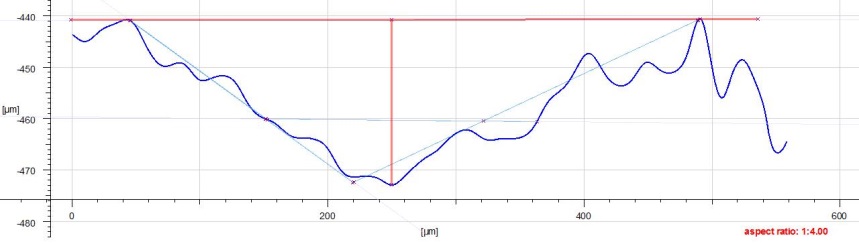 |
| --- | --- |

Figure SI1-16: a) 3D Alicona image of the incised surface of the human radius. The blue line indicates where the profile measurements were taken; b-c) profiles of the incision with and without measurements.

This is a single large and deep incision produced by scraping. The incision seems to have been intentionally enlarged by moving the stone tool side-wise toward the end portion of the incision, but by keeping the stone tool fixed at the starting point of the incision. This process has produced a V-shape incision on the surface of the radius. The full length of the incision is 1.37 mm. The values of the profile measurements, taken at the incision’s midpoint, are:

WIS = 444.98 µm

WIB = 212.7 µm

OA = 162.95˚

D = 32.44 µm

ATI = 88.27˚

**Engraving 17**

| 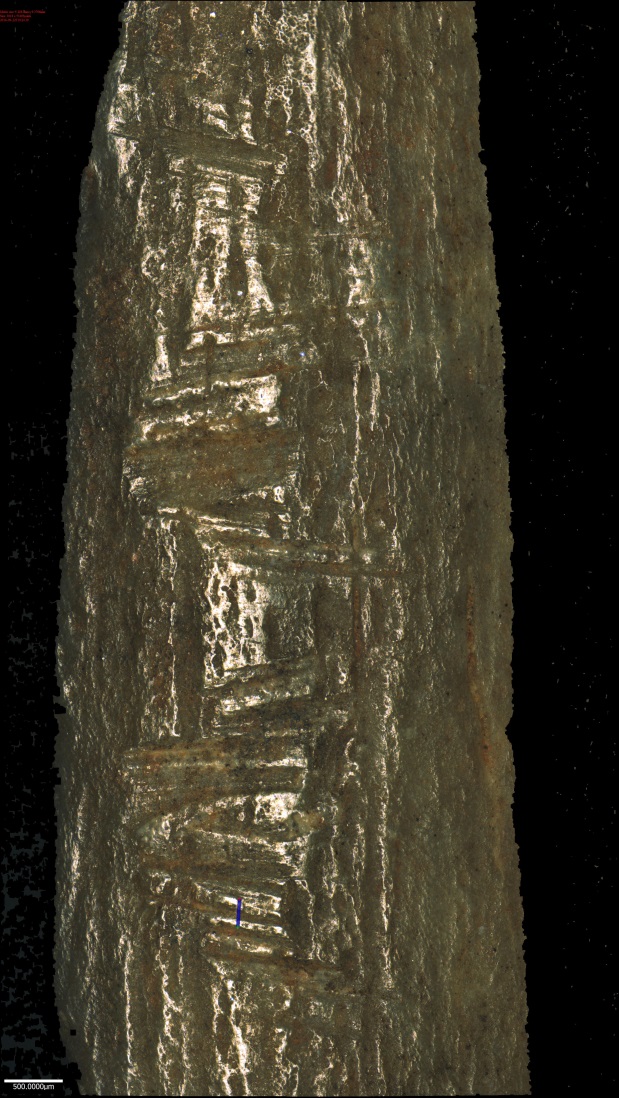 | 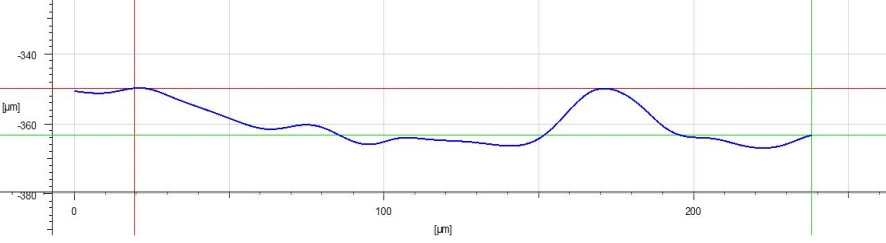  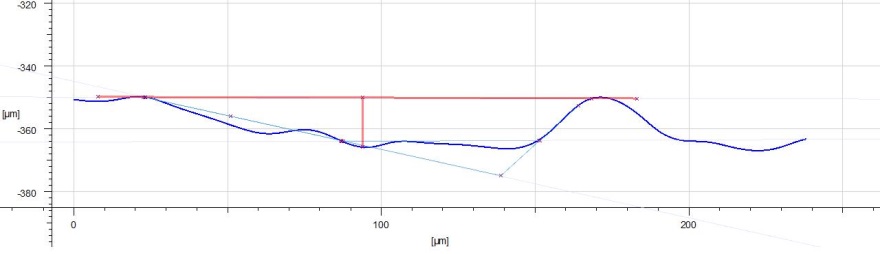 |
| --- | --- |

Figure SI1-17: a) 3D Alicona image of the incised surface of the human radius. The blue line indicates where the profile measurements were taken; b-c) profiles of the incision with and without measurements.

This is a single-stroke incision probably produced by a unilaterally retouched blade. The incision is very similar to incision 18, though incision 18 is shorter. The full length of this incision is 1.35 mm. The values of the profile measurements, taken at the incision’s midpoint, are:

WIS = 145.32 µm

WIB = 64.16 µm

OA = 126.00˚

D = 15.89 µm

ATI = 104.66˚

**Engraving 18**

| 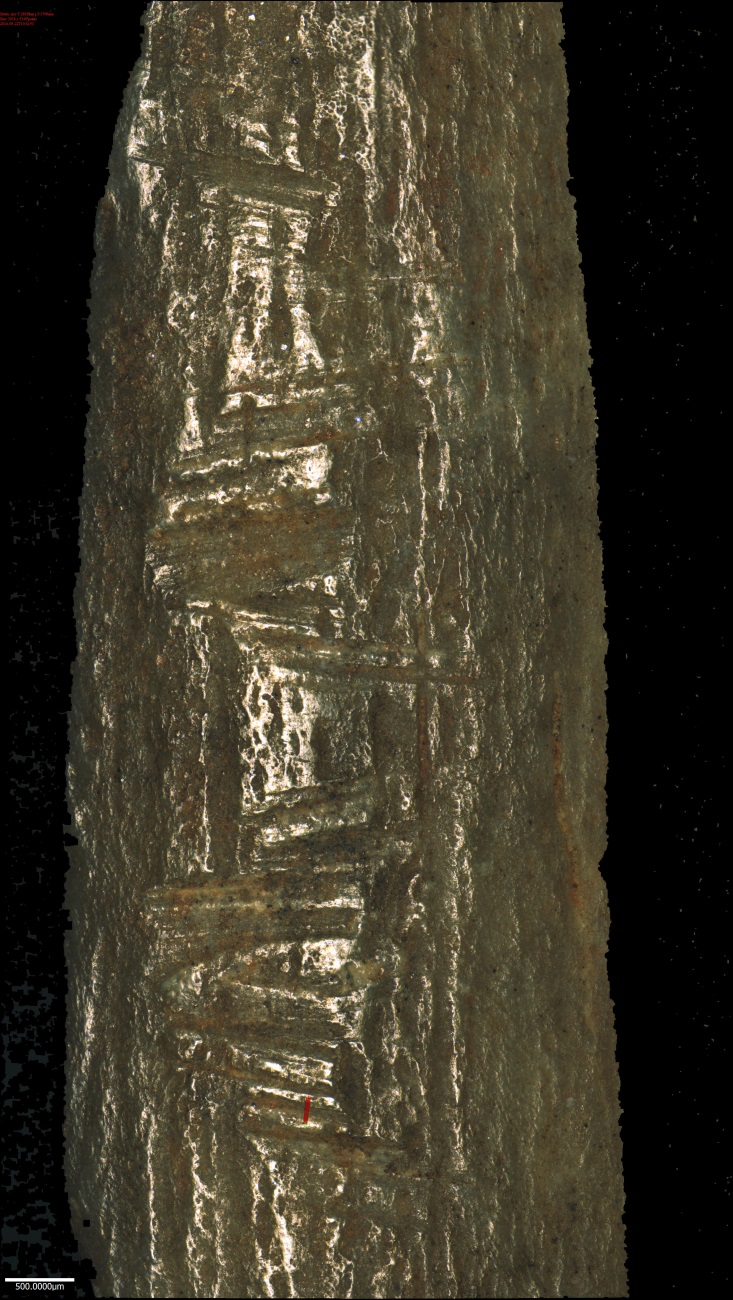 | 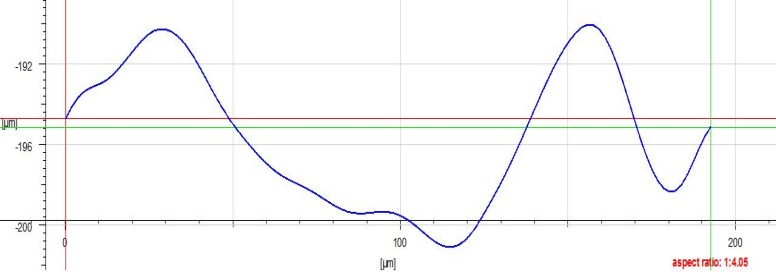  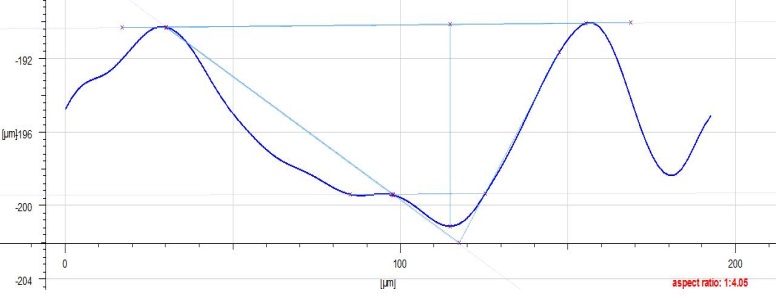 |
| --- | --- |

Figure SI1-18: a) 3D Alicona image of the incised surface of the human radius. The blue line indicates where the profile measurements were taken; b-c) profiles of the incision with and without measurements.

This is a single-stroke incision probably produced by a unilaterally retouched blade. The incision (its profile and micro-morphometric characteristics) is very similar to incision 17. The full length of the incision is 0.91 mm. The values of the profile measurements, taken at the incision’s midpoint, are:

WIS = 125.27 µm

WIB = 40.24 µm

OA = 153.04˚

D = 11.08 µm

ATI = 95.75˚

**Engraving 19**

| 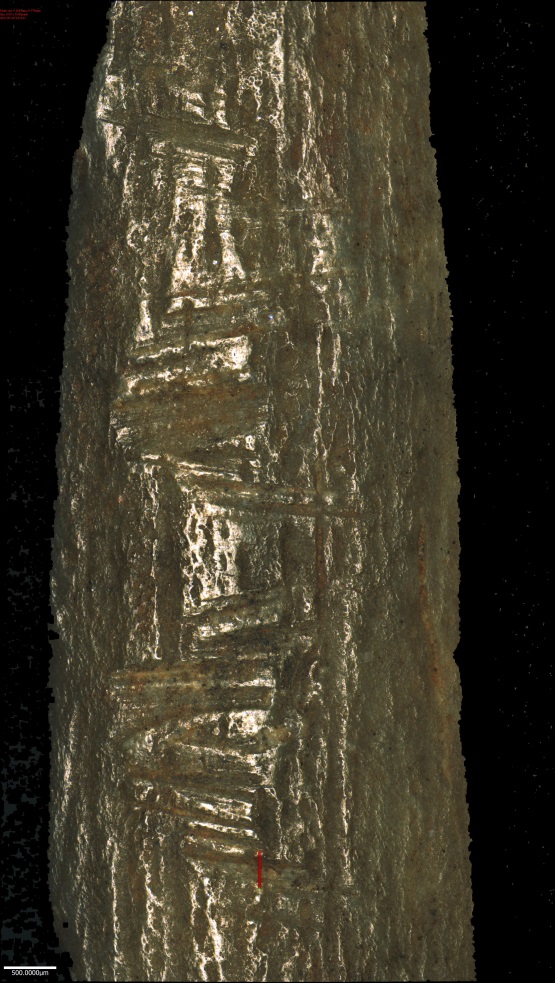 | 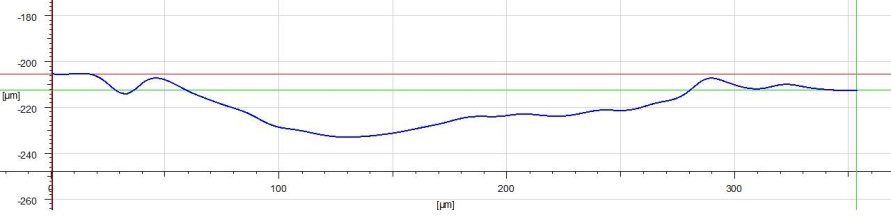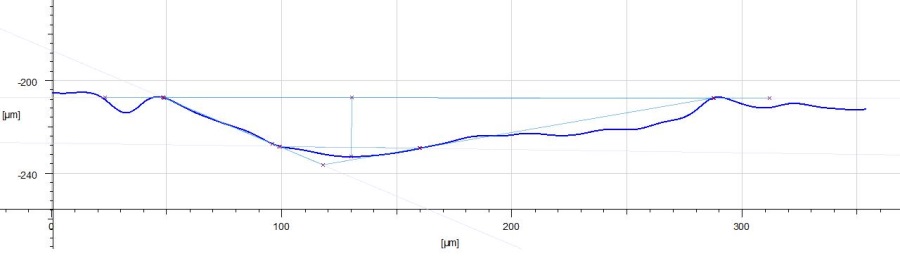 |
| --- | --- |

Figure SI1-19: a) 3D Alicona image of the incised surface of the human radius. The blue line indicates where the profile measurements were taken; b-c) profiles of the incision with and without measurements.

This is a wide single incision produced by a to-and-fro action, and is 1.62 mm long. The internal sub-incisions overlap perfectly giving the impression of a single large incision. The values of the profile measurements, taken at the incision’s midpoint, are:

WIS = 239.96 µm

WIB = 60.76 µm

OA = 147.47˚

D = 25.84 µm

ATI = 83.38˚

**Engraving 20**

| 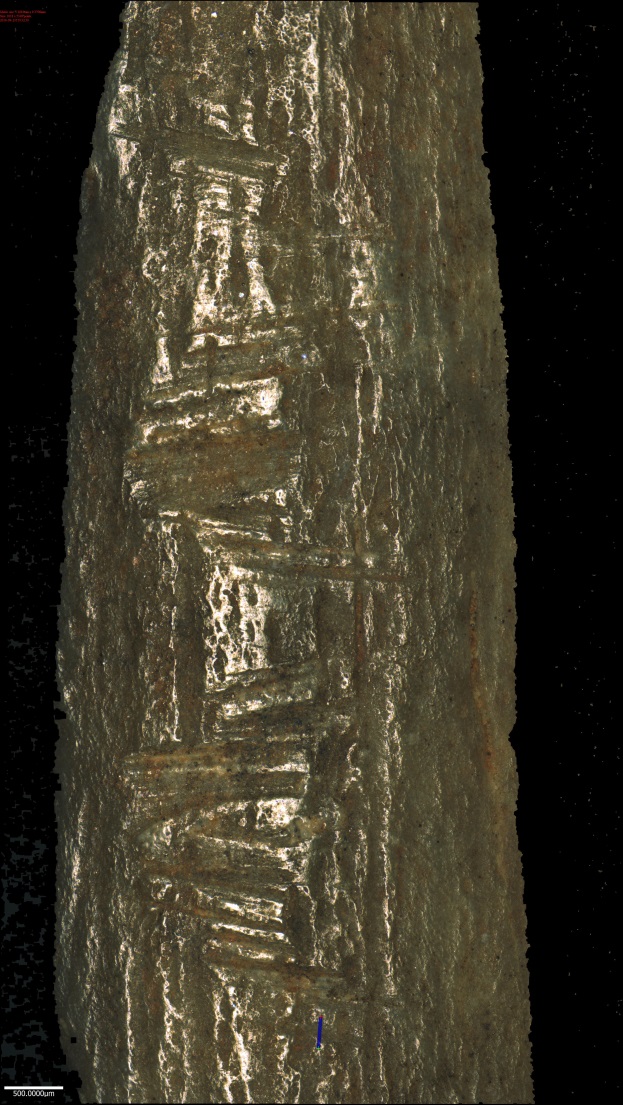 | 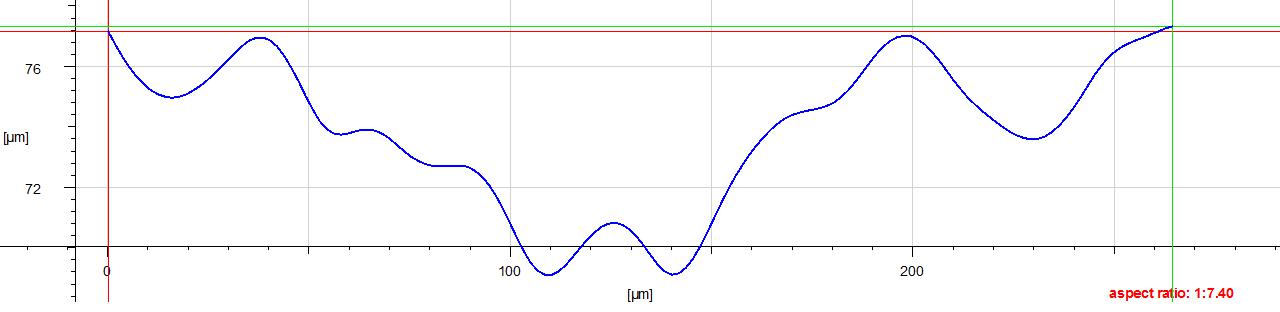  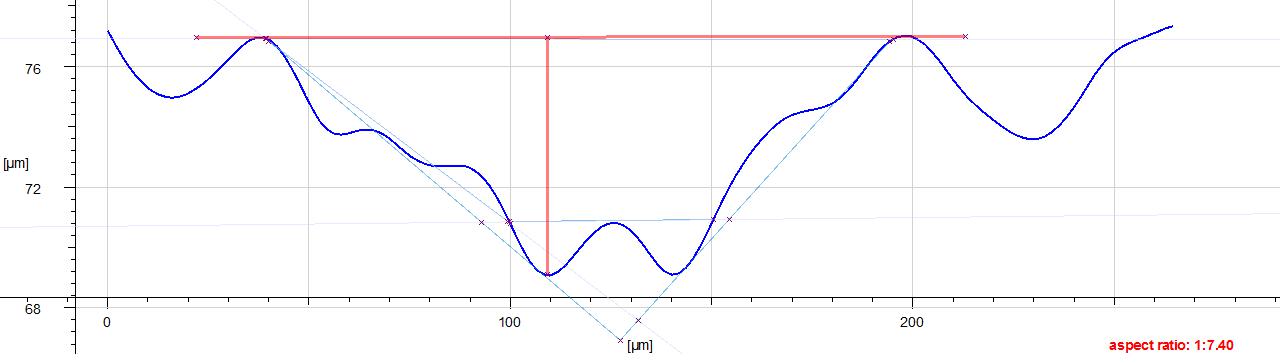 |
| --- | --- |

Figure SI1-20: a) 3D Alicona image of the incised surface of the human radius. The blue line indicates where the profile measurements were taken; b-c) profiles of the incision with and without measurements.

This is a single-stroke incision, located slightly apart from the previous cluster of cuts, probably produced by a unilaterally retouched blade. The full length of the incision is 1.14 mm. The values of the profile measurements, taken at the incision’s midpoint, are:

WIS = 155.99 µm

WIB = 50.7 µm

OA = 165.01˚

D = 7.94 µm

ATI = 91.70˚

**Engraving 21**

| 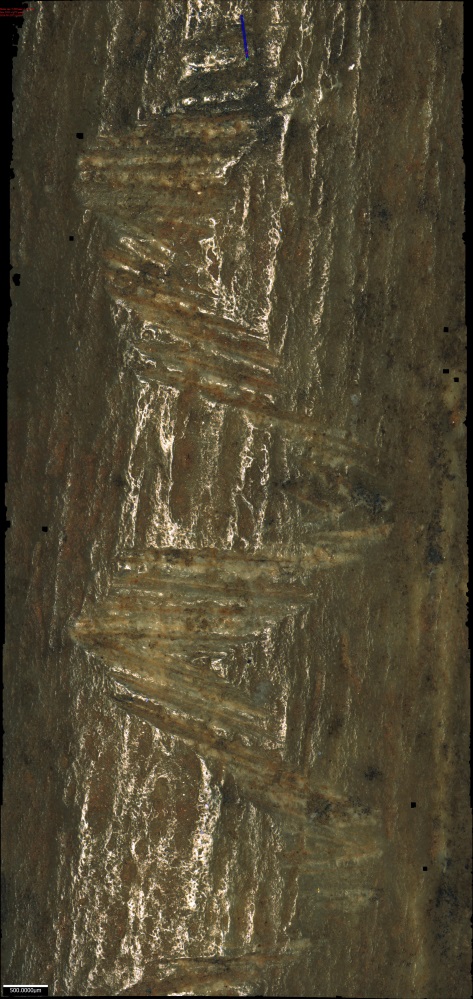 | 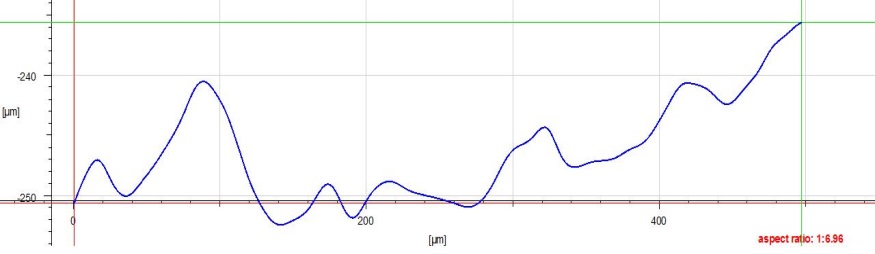  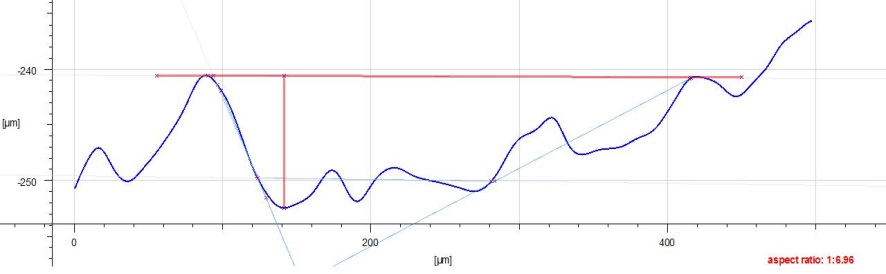 |
| --- | --- |

Figure SI1-21: a) 3D Alicona image of the incised surface of the human radius. The blue line indicates where the profile measurements were taken; b-c) profiles of the incision with and without measurements.

This is a wide though very shallow single-stroke incision. The incision is slightly apart from the previous and successive cluster of cuts. The incision was probably produced by a unilaterally retouched blade. The full length of the incision is 1.25 mm. The values of the profile measurements, taken at the incision’s midpoint, are:

WIS = 326.64 µm

WIB = 157.93 µm

OA = 158.14˚

D = 11.95 µm

ATI = 83.65˚

**Engraving 22**

| 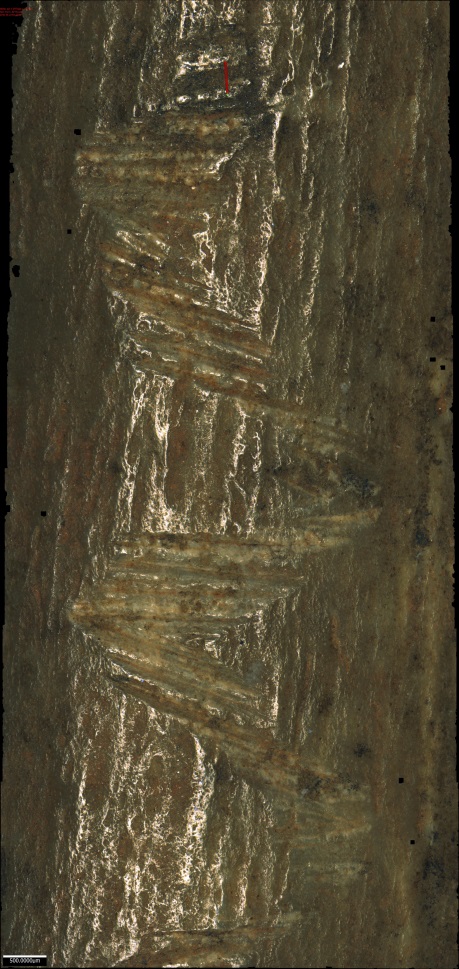 | 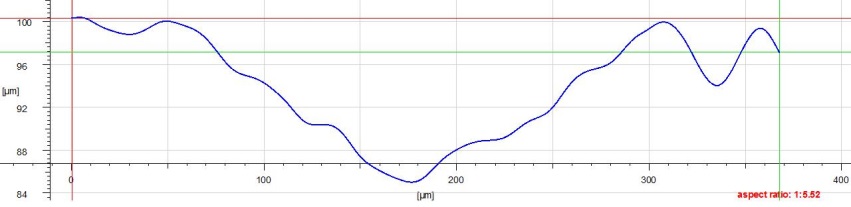  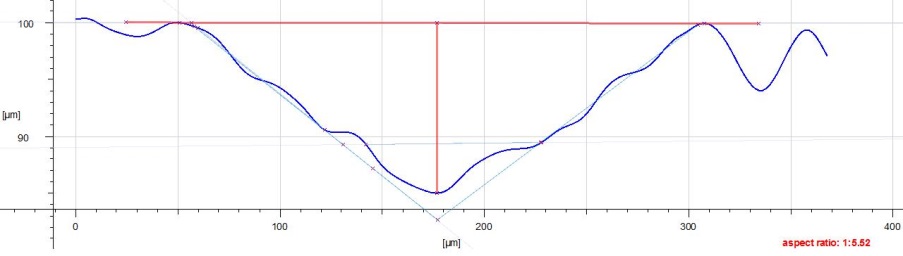 |
| --- | --- |

Figure SI1-22: a) 3D Alicona image of the incised surface of the human radius. The blue line indicates where the profile measurements were taken; b-c) profiles of the incision with and without measurements.

This is a wide single-stroke incision parallel to incision 21 and slightly apart from the successive cluster of cuts. The full length of the incision is 1.22 mm. The values of the profile measurements, taken at the incision’s midpoint, are:

WIS = 257.52 µm

WIB = 85.95 µm

OA = 171.74˚

D = 15.08 µm

ATI = 89.68˚

**Engraving 23**

| 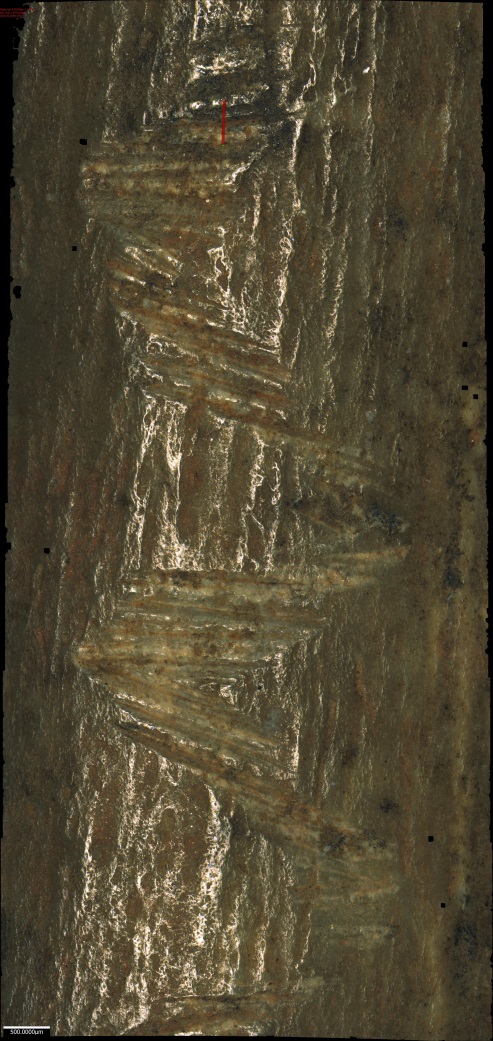 | 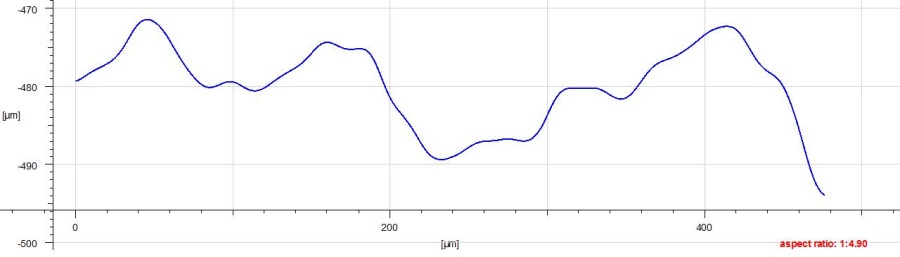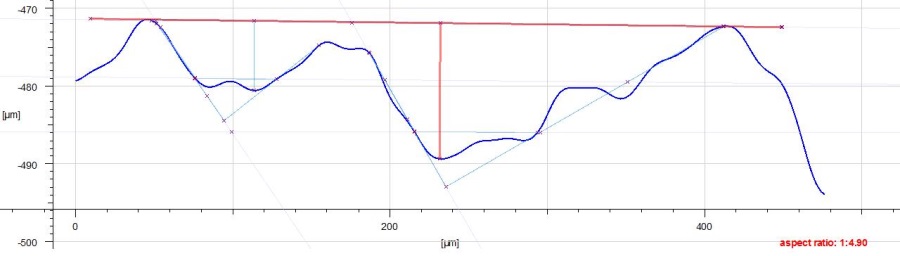 |
| --- | --- |

Figure SI1-23: a) 3D Alicona image of the incised surface of the human radius. The blue line indicates where the profile measurements were taken; b-c) profiles of the incision with and without measurements.

This is a double incision produced by a to-and-fro sawing action, although the second incision (toward the proximal end of the diaphysis, at the top of the image) could be a scratch produced by the irregularities of the stone tool (cf. shoulder effect). We divided incision 23 into sub-incisions A (toward the proximal epiphysis) and B (toward distal epiphysis).The total width of the double incision is 364.29 µm, sub-incision A is 1.36 mm long and sub-incision B is 1.99 mm long. The values of the profile measurements, taken at the incision’s midpoint, are:

WIB = 52.22 µm and 78.41 µm

OA = 154.58˚ and 153.83˚

D = 9.03 µm and 17.60 µm

ATI = 86.02˚ and 83.50˚

**Engraving 24**

| 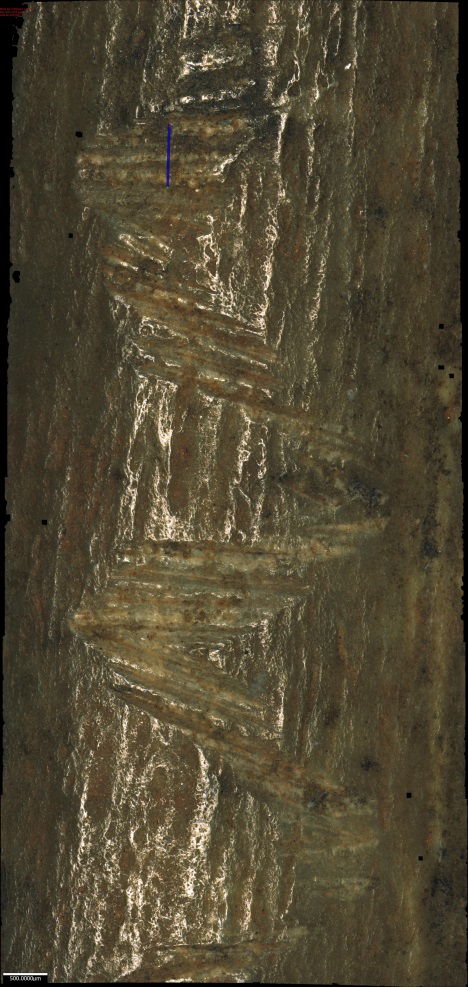 | 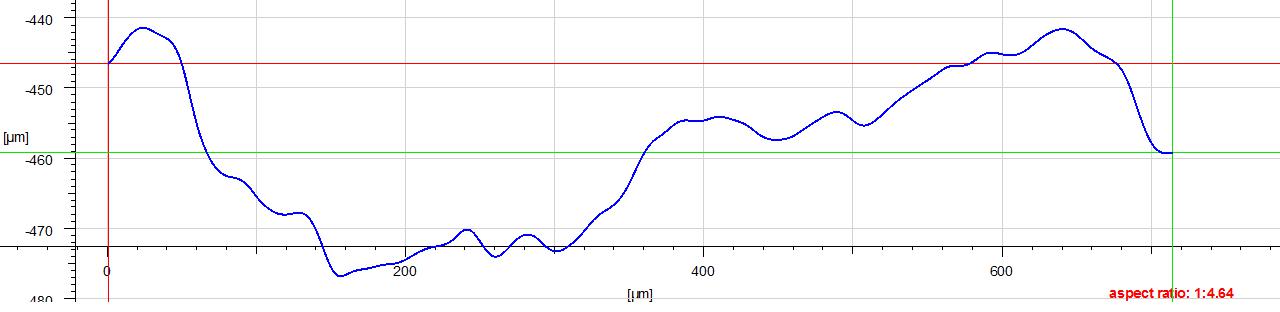  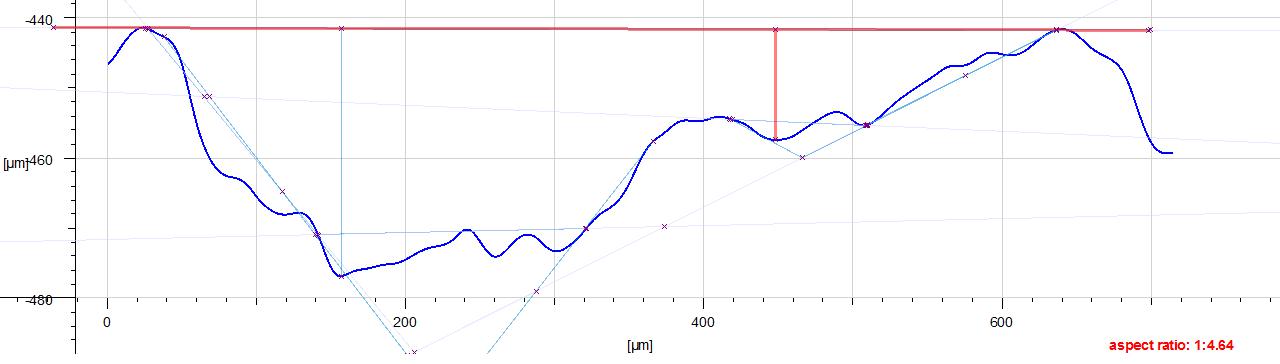 |
| --- | --- |

Figure SI1-24: a) 3D Alicona image of the incised surface of the human radius. The blue line indicates where the profile measurements were taken; b-c) profiles of the incision with and without measurements.

This is a wide and deep double incision produced by scraping. We divided incision 24 into sub-incisions A (toward the proximal epiphysis) and B (toward distal epiphysis).The total width of the double incision is 612.08 µm, sub-incision A is 1.80 mm long and sub-incision B is 1.63 mm long. The values of the profile measurements, taken at the incision’s midpoint, are:

WIB = 180.45 µm and 89.85 µm

OA = 149.03˚ and 167.26˚

D = 35.63 µm and 15.89 µm

ATI = 90.90˚ and 89.75˚

**Engraving 25**

| 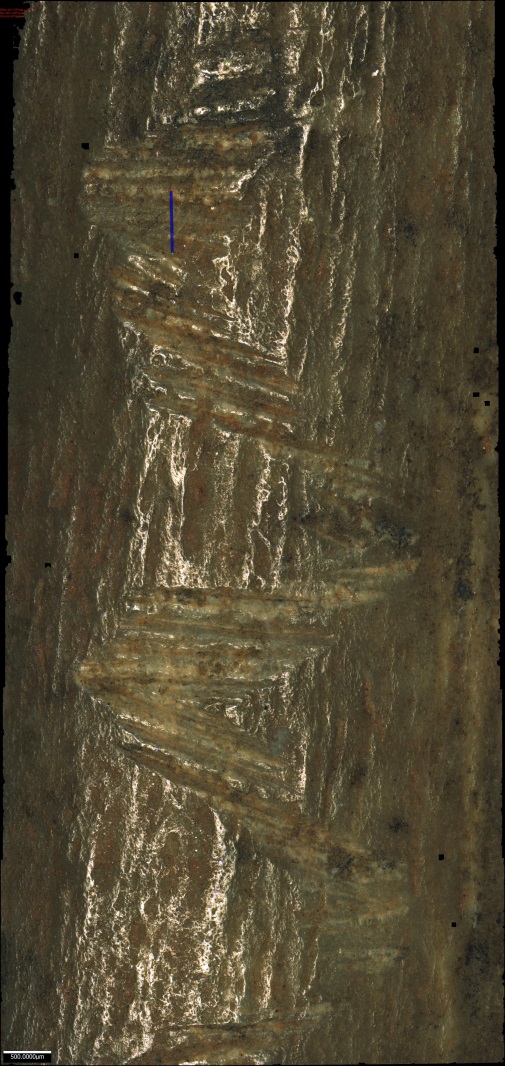 | 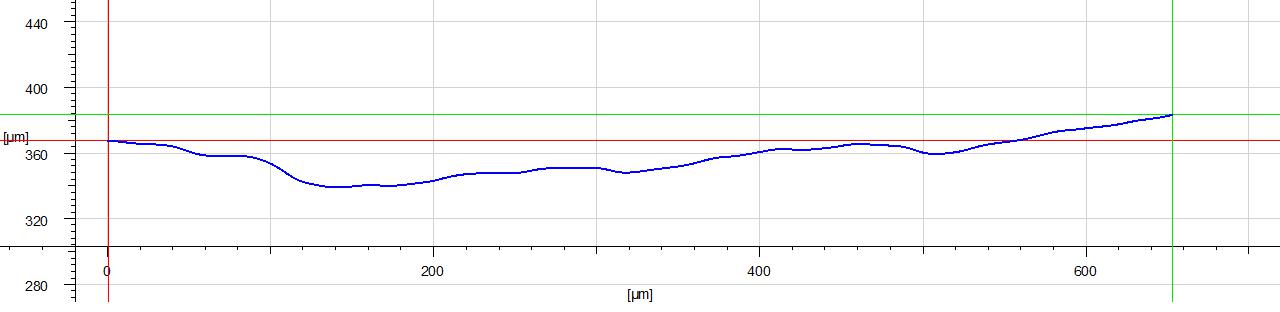  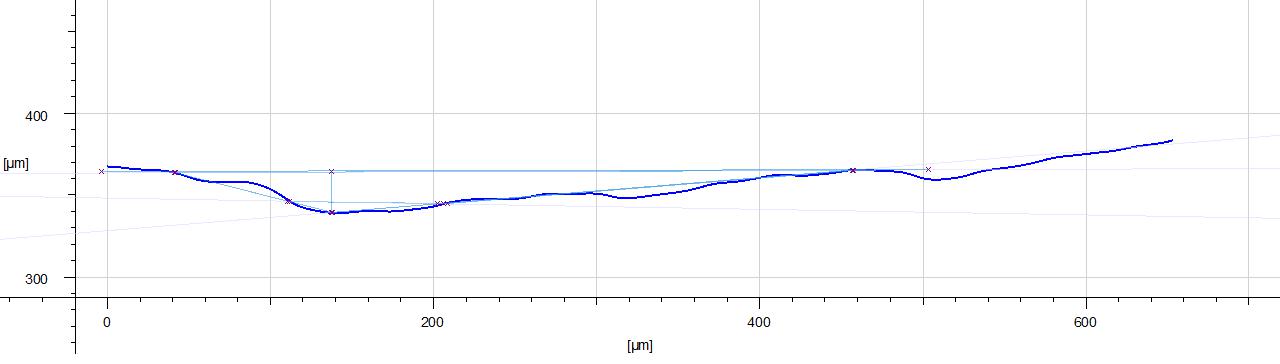 |
| --- | --- |

Figure SI1-25: a) 3D Alicona image of the incised surface of the human radius. The blue line indicates where the profile measurements were taken; b-c) profiles of the incision with and without measurements.

This is a wide and deep single incision produced by scraping. No clear internal sub-incisions are distinguishable. The full length of the incision is 1.80 mm. The values of the profile measurements, taken at the incision’s midpoint, are:

WIS = 415.72 µm

WIB = 94.54 µm

OA = 161.15˚

D = 25.51 µm

ATI = 85.23˚

**Engraving 26**

| 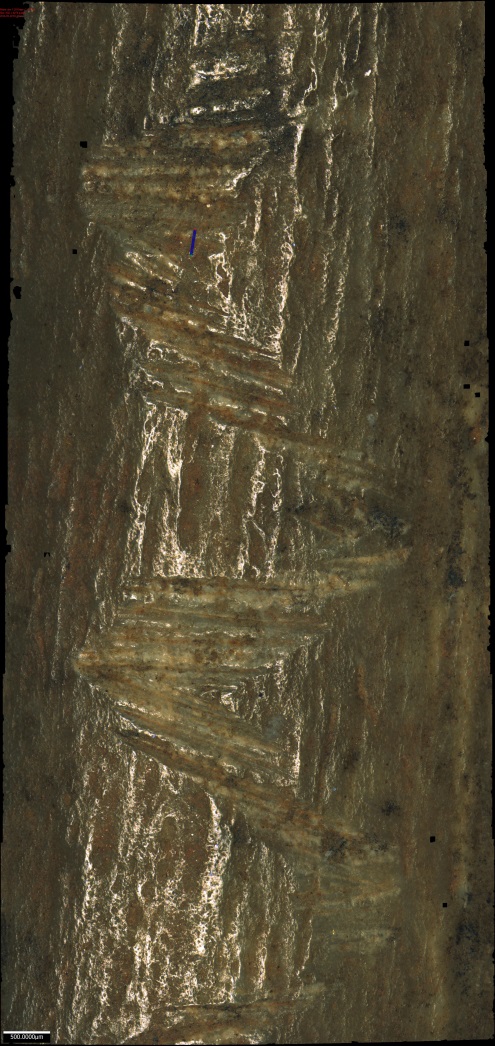 | 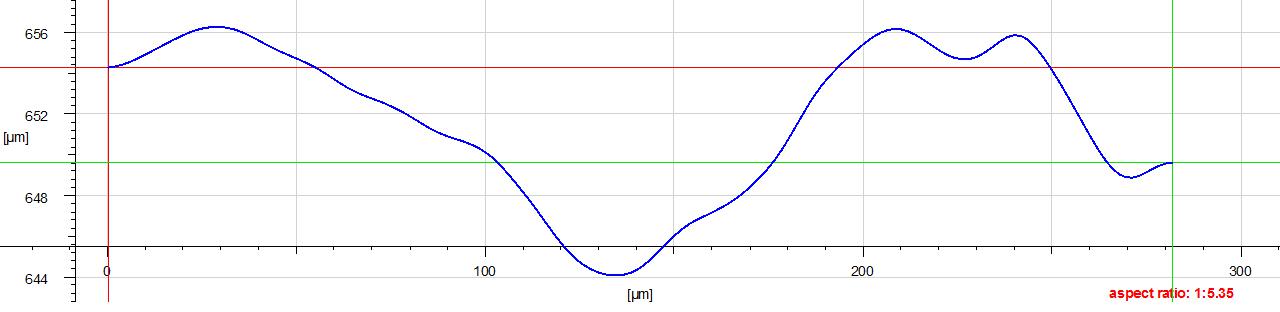  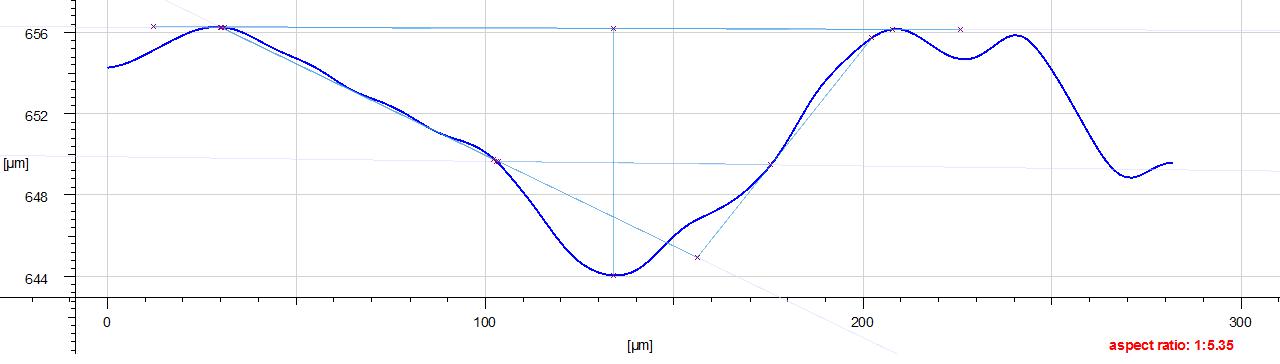 |
| --- | --- |

Figure SI1-26: a) 3D Alicona image of the incised surface of the human radius. The blue line indicates where the profile measurements were taken; b-c) profiles of the incision with and without measurements.

This is a single-stroke incision, partially bisected by incision 25. The preserved portion of the incision is 0.99 mm long. The values of the profile measurements, taken at the incision’s midpoint, are:

WIS = 177.22 µm

WIB = 72.77 µm

OA = 161.65˚

D = 12.22 µm

ATI = 94.02˚

**Engraving 27**

| 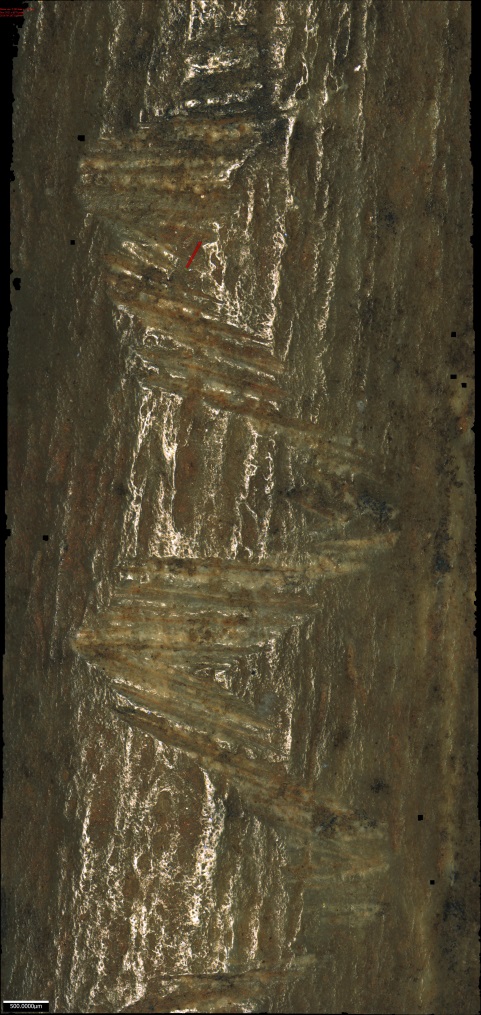 | 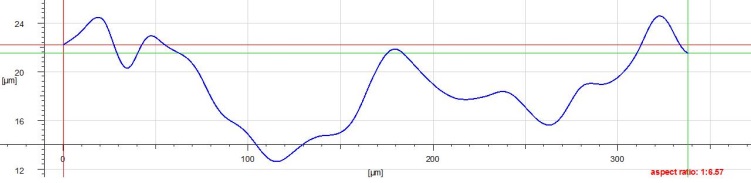  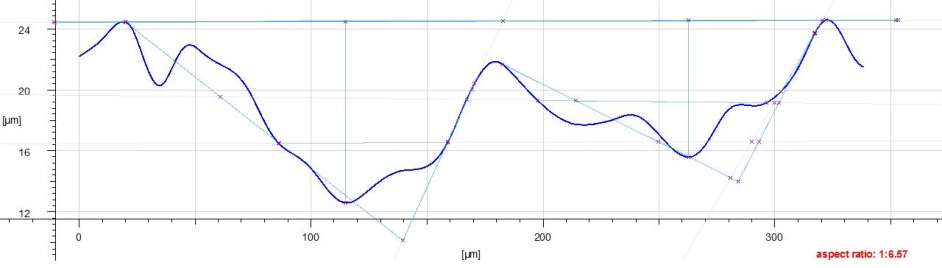 |
| --- | --- |

Figure SI1-27: a) 3D Alicona image of the incised surface of the human radius. The blue line indicates where the profile measurements were taken; b-c) profiles of the incision with and without measurements.

This is a double incision, produced by a to-and-fro action. The two sub-incisions do not overlap. Although the end point of the first sub-incision (toward the distal epiphysis) coincides with the starting point of the second one, the end point of the second sub-incision diverges, giving an overall superficial V-shape to the incision. The incision, probably produced by a unilaterally retouched blade, has been divided into sub-incisions A (toward the proximal epiphysis) and B (toward distal epiphysis). The total width of the double incision is 302.60 µm, sub-incision A is 0.69 mm long and sub-incision B is 1.3 mm long. The values of the profile measurements, taken at the incision’s midpoint, are:

WIB = 73.05 µm and 98.87 µm

OA = 154.69˚ and 159.21˚

D = 11.94 µm and 9.00 µm

ATI = 95.85˚ and 94.29˚

**Engraving 28**

| 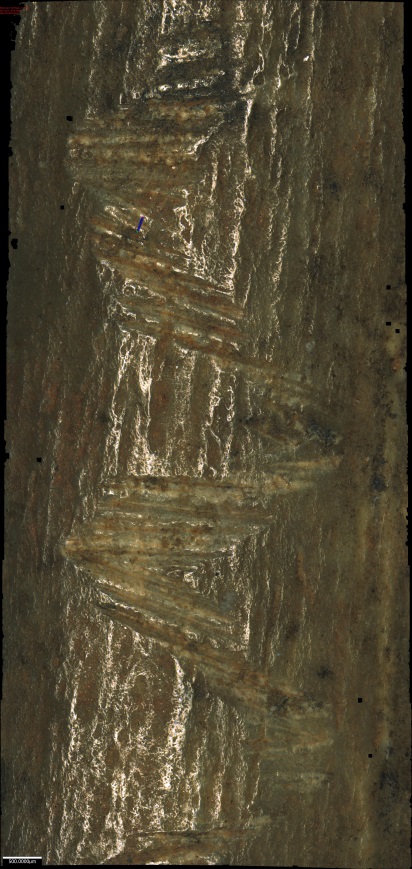 | 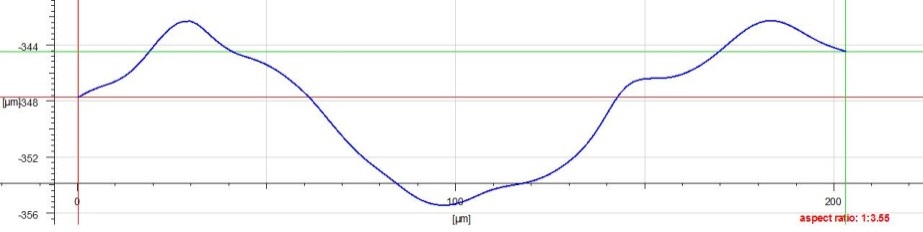  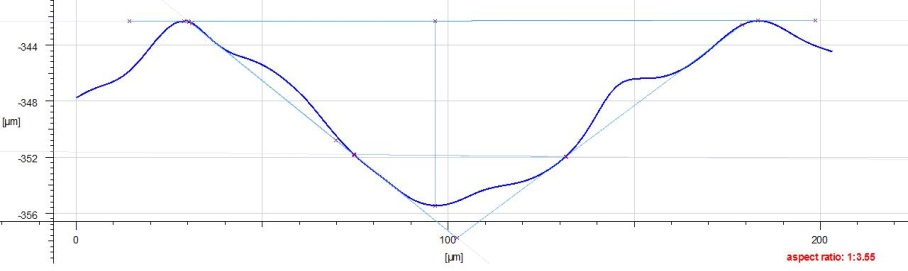 |
| --- | --- |

Figure SI1-28: a) 3D Alicona image of the incised surface of the human radius. The blue line indicates where the profile measurements were taken; b-c) profiles of the incision with and without measurements.

This is a single-stroke incision, 1.08 mm long. The values of the profile measurements, taken at the incision’s midpoint, are:

WIS = 154.53 µm

WIB = 57.02 µm

OA = 156.52˚

D = 13.26 µm

ATI = 89.62˚

**Engraving 29**

| 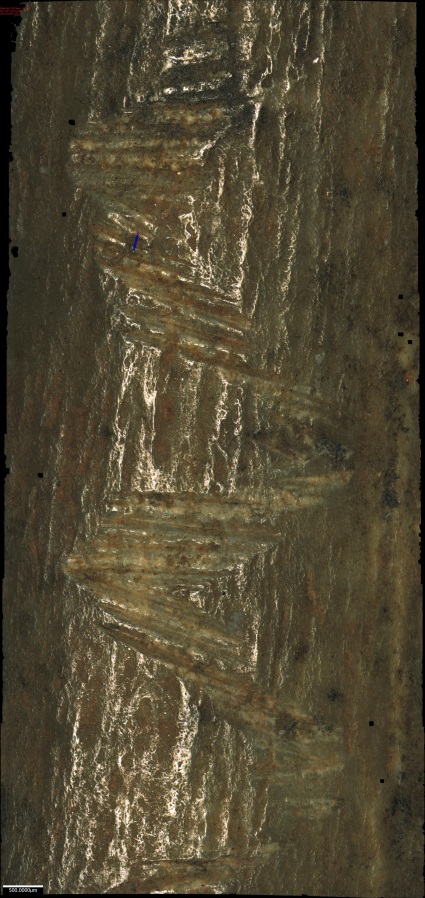 | 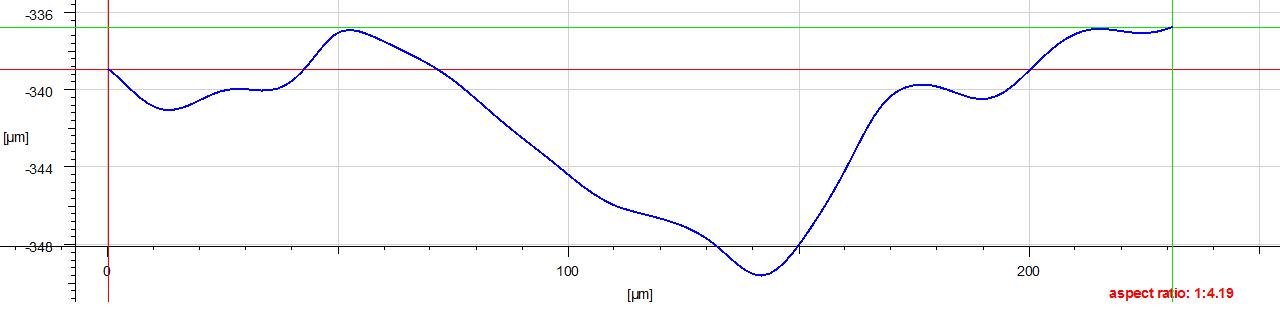  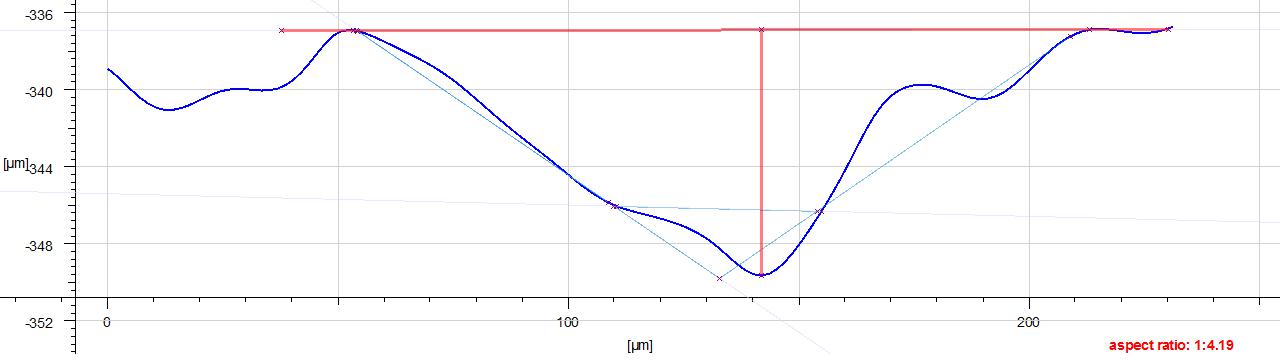 |
| --- | --- |

Figure SI1-29: a) 3D Alicona image of the incised surface of the human radius. The blue line indicates where the profile measurements were taken; b-c) profiles of the incision with and without measurements.

This is a single-stroke incision, probably produced by a unilaterally retouched blade. It is 1.43 mm long. The values of the profile measurements, taken at the incision’s midpoint, are:

WIS = 160.02 µm

WIB = 44.57 µm

OA = 161.28˚

D = 12.82 µm

ATI = 90.06˚

**Engraving 30**

| 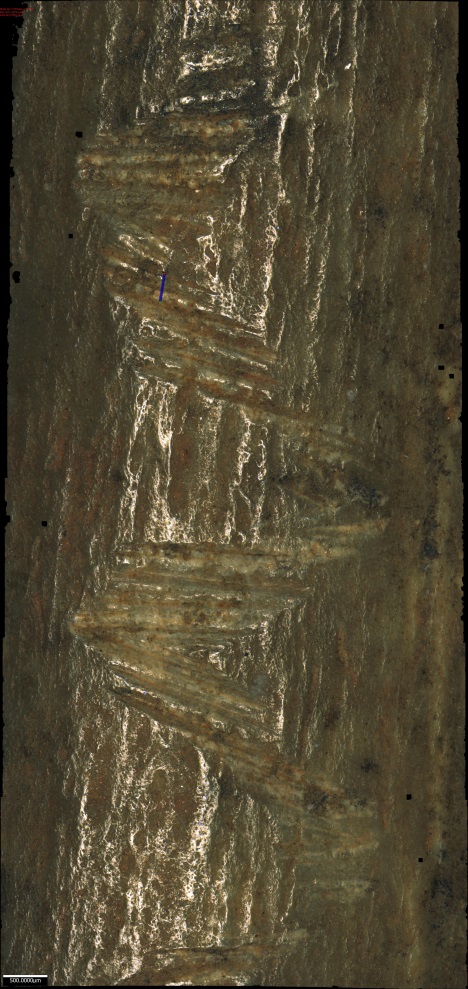 | 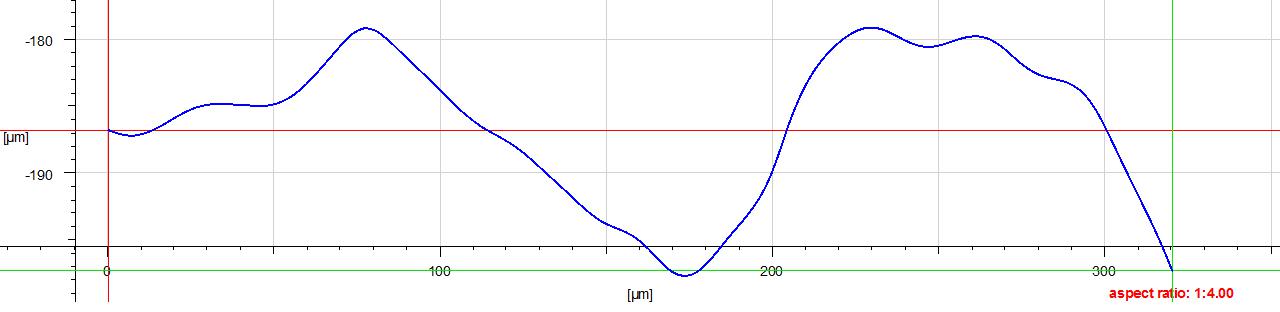  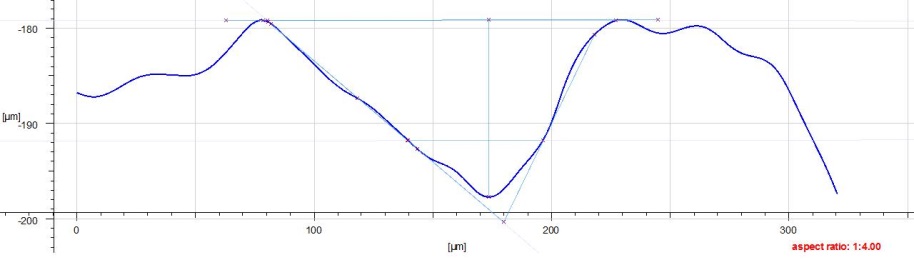 |
| --- | --- |

Figure SI1-30: a) 3D Alicona image of the incised surface of the human radius. The blue line indicates where the profile measurements were taken; b-c) profiles of the incision with and without measurements.

This is a single-stroke incision, probably produced by an unretouched blade. It is 1.58 mm long. The values of the profile measurements, taken at the incision’s midpoint, are:

WIS = 149.03 µm

WIB = 56.77 µm

OA = 140.65˚

D = 18.73 µm

ATI = 97.58˚

**Engraving 31**

| 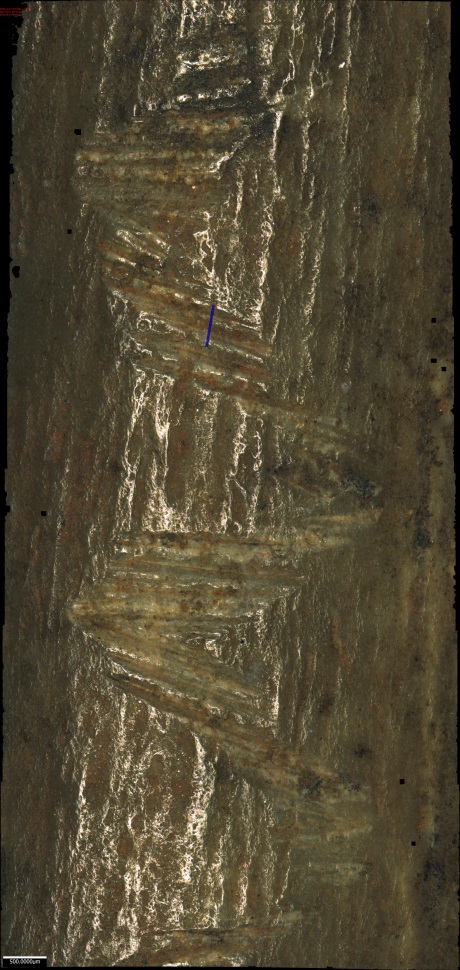 | 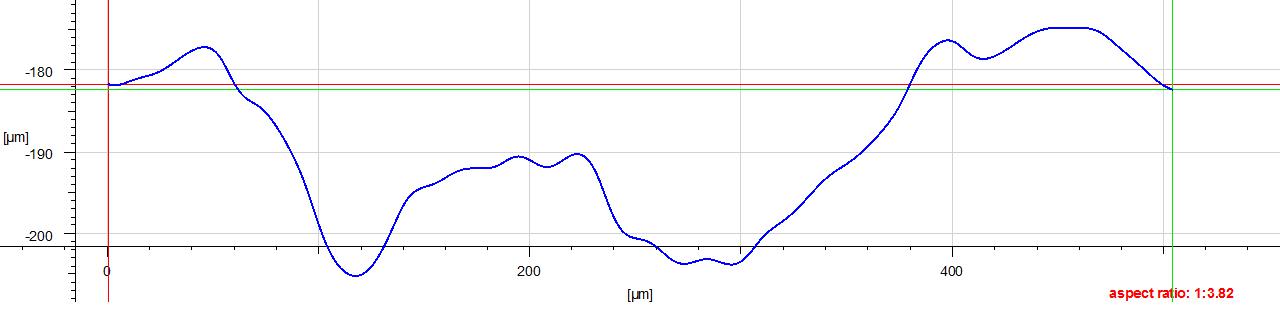  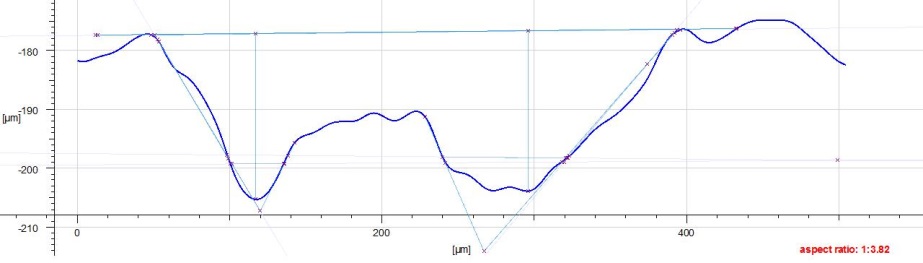 |
| --- | --- |

Figure SI1-31: a) 3D Alicona image of the incised surface of the human radius. The blue line indicates where the profile measurements were taken; b-c) profiles of the incision with and without measurements.

This is a double incision produced by a to-and-fro sawing action. Similarly to incision 27, the two sub-incisions do not overlap. The end point of the first sub-incision (toward the distal epiphysis) intersects with the starting point of the second one. However, the end point of the second sub-incision diverges, giving an overall superficial V-shape to the incision. The incision has been divided into sub-incisions A (toward the proximal epiphysis) and B (toward distal epiphysis). The total width of the double incision is 348.33 µm, sub-incision A is 1.85 mm long and sub-incision B is 1.89 mm long. The values of the profile measurements, taken at the incision’s midpoint, are:

WIB = 34.98 µm and 81.73 µm

OA = 129.26˚ and 132.62˚

D = 28.43 µm and 27.46 µm

ATI = 91.92˚ and 83.38˚

**Engraving 32**

| 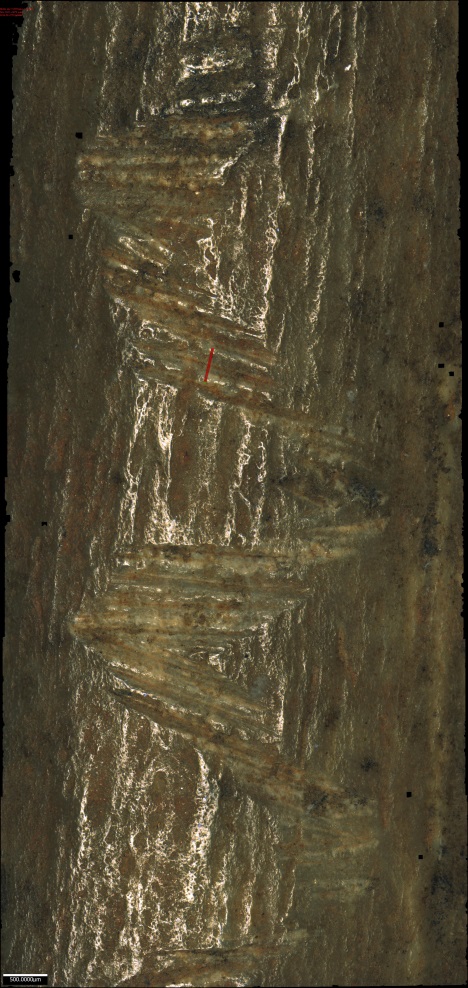 | 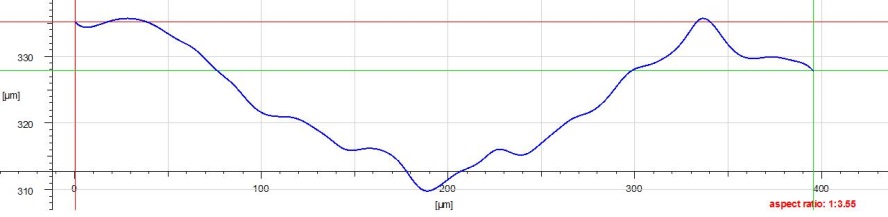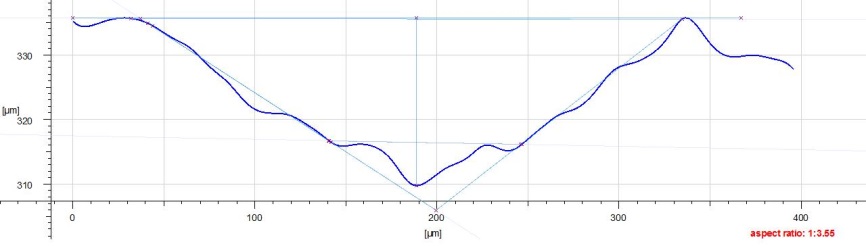 |
| --- | --- |

Figure SI1-32: a) 3D Alicona image of the incised surface of the human radius. The blue line indicates where the profile measurements were taken; b-c) profiles of the incision with and without measurements.

This is a large single incision produced by a to-and-fro action, probably through use of a unilaterally retouched blade. No internal sub-incisions are recognisable, due to complete overlapping. The incision is 1.85 mm long and the values of the profile measurements, taken at the incision’s midpoint, are:

WIS = 303.32 µm

WIB = 106.03 µm

OA = 157.18˚

D = 26.12 µm

ATI = 90.99˚

**Engraving 33**

| 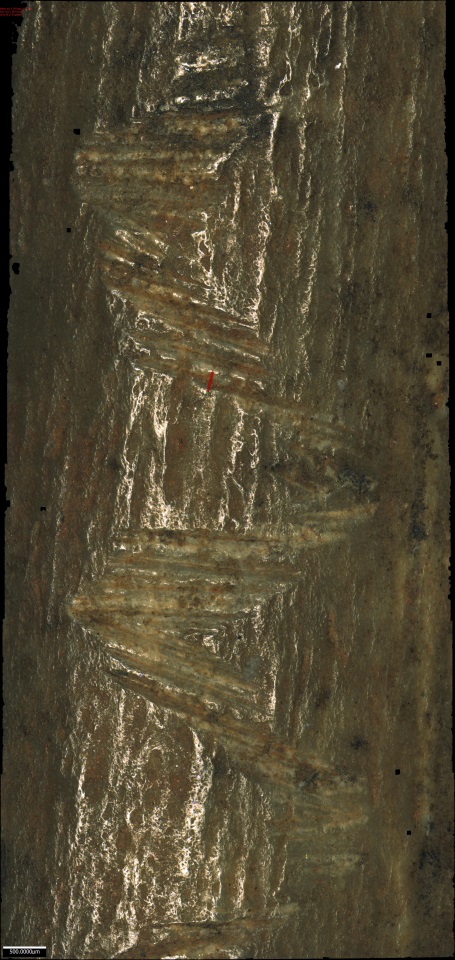 | 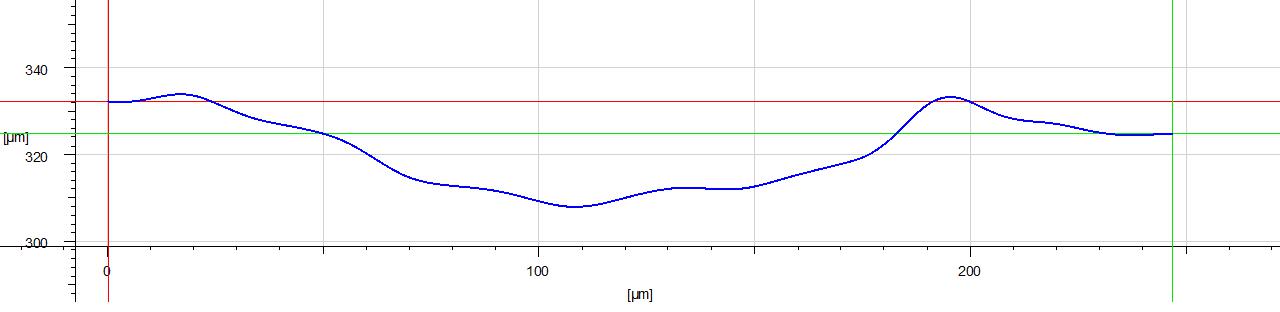  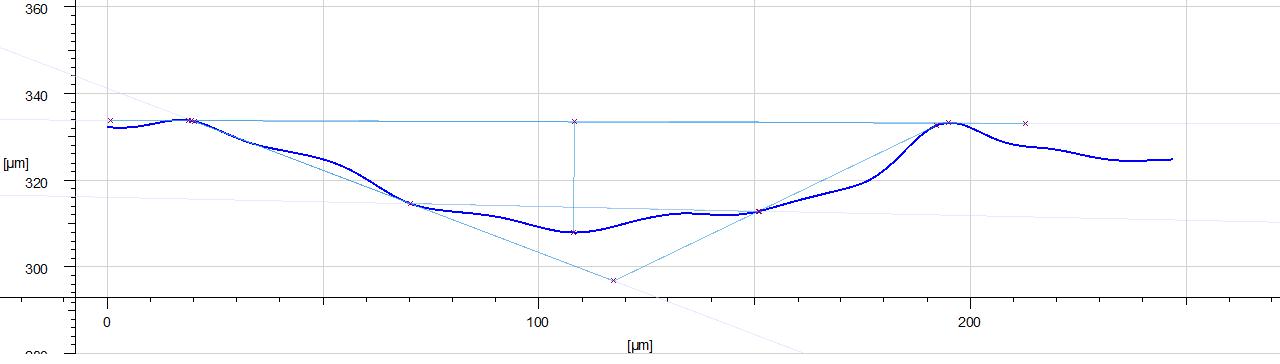 |
| --- | --- |

Figure SI1-33: a) 3D Alicona image of the incised surface of the human radius. The blue line indicates where the profile measurements were taken; b-c) profiles of the incision with and without measurements.

This is a single-stroke incision, 2.47 mm long. The values of the profile measurements, taken at the incision’s midpoint, are:

WIS = 176.34 µm

WIB = 80.91 µm

OA = 133.46˚

D = 25.75 µm

ATI = 92.45˚

**Engraving 34**

| 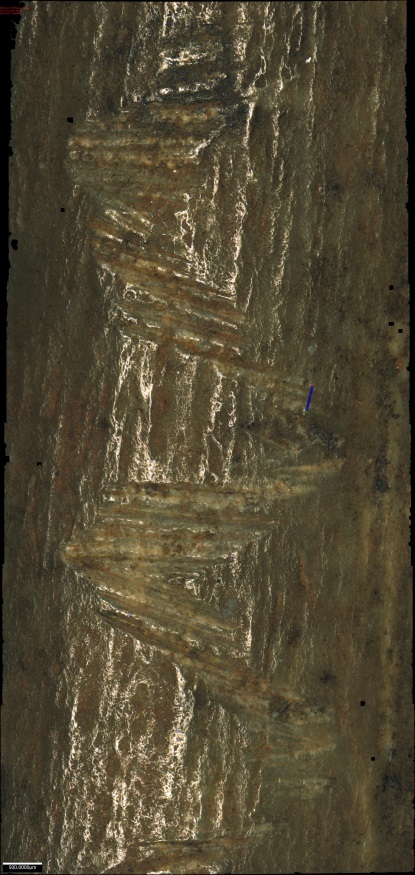 |  |
| --- | --- |

Figure SI1-34: a) 3D Alicona image of the incised surface of the human radius. The blue line indicates where the profile measurements were taken; b-c) profiles of the incision with and without measurements.

This is a double incision, produced by a to-and-fro sawing action. The two sub-incisions are parallel and very close to each other, so much as to partially overlap. The incision has been divided into sub-incisions A (toward the proximal epiphysis) and B (toward distal epiphysis).The total width of the double incision is 311.31 µm, sub-incision A is 1.25 mm long and sub-incision B is 1.23 mm long. The values of the profile measurements, taken at the incision’s midpoint, are:

WIB = 56.69 µm and 91.15 µm

OA = 150.17˚ and 143.45˚

D = 16.77 µm and 13.67 µm

ATI = 86.25˚ and 97.82˚

**Engraving 35**

|  |  |
| --- | --- |

Figure SI1-35: a) 3D Alicona image of the incised surface of the human radius. The blue line indicates where the profile measurements were taken; b-c) profiles of the incision with and without measurements.

This is a single-stroke incision, 1.23 mm long. The values of the profile measurements, taken at the incision’s midpoint, are:

WIS = 243.24 µm

WIB = 55.28 µm

OA = 157.37˚

D = 23.55 µm

ATI = 89.40˚

**Engraving 36**

|  |  |
| --- | --- |

Figure SI1-36: a) 3D Alicona image of the incised surface of the human radius. The blue line indicates where the profile measurements were taken; b-c) profiles of the incision with and without measurements.

This is a single-stroke incision, 0.81 mm long. The values of the profile measurements, taken at the incision’s midpoint, are:

WIS = 324.53 µm

WIB = 103.91 µm

OA = 158.24˚

D = 24.15 µm

ATI = 90.13˚

**Engraving 37**

|  |  |
| --- | --- |

Figure SI1-37: a) 3D Alicona image of the incised surface of the human radius. The blue line indicates where the profile measurements were taken; b-c) profiles of the incision with and without measurements.

This is a single-stroke incision, probably produced by a unilaterally retouched blade, which intersects incision 36 towards its end. This suggests that incision 35 was made prior to incision 36, which is contrary to the general succession of the engraving (from the distal toward the proximal end of the radius). Incision 36 is 1.07 mm long and the values of the profile measurements, taken at the incision’s midpoint, are:

WIS = 293.29 µm

WIB = 47.85 µm

OA = 155.45˚

D = 20.23 µm

ATI = 82.74˚

**Engraving 38**

|  |  |
| --- | --- |

Figure SI1-38: a) 3D Alicona image of the incised surface of the human radius. The blue line indicates where the profile measurements were taken; b-c) profiles of the incision with and without measurements.

This is a single-stroke incision, probably produced by a unilaterally retouched blade. It is 2.47 mm long and the values of the profile measurements, taken at the incision’s midpoint, are:

WIS = 258.78 µm

WIB = 88.84 µm

OA = 149.45˚

D = 28.45 µm

ATI = 88.49˚

**Engraving 39**

|  |  |
| --- | --- |

Figure SI1-39: a) 3D Alicona image of the incised surface of the human radius. The blue line indicates where the profile measurements were taken; b-c) profiles of the incision with and without measurements.

This is a single incision produced by a to-and-fro action, probably by a unilaterally retouched blade. This incision is very similar to incision 38. The incision is 2.33 mm long and the values of the profile measurements, taken at the incision’s midpoint, are:

WIS = 300.91 µm

WIB = 68.67 µm

OA = 156.53˚

D = 30.62 µm

ATI = 88.13˚

**Engraving 40**

|  |  |
| --- | --- |

Figure SI1-40: a) 3D Alicona image of the incised surface of the human radius. The blue line indicates where the profile measurements were taken; b-c) profiles of the incision with and without measurements.

This is a single-stroke incision produced by a unilaterally retouched blade. It is 2.24 mm long and the values of the profile measurements, taken at the incision’s midpoint, are:

WIS = 238.23 µm

WIB = 92.53 µm

OA = 134.32˚

D = 26.14 µm

ATI = 75.56˚

**Engraving 41**

|  |  |
| --- | --- |

Figure SI1-41: a) 3D Alicona image of the incised surface of the human radius. The blue line indicates where the profile measurements were taken; b-c) profiles of the incision with and without measurements.

This is a single-stroke incision, 2.10 mm long. The values of the profile measurements, taken at the incision’s midpoint, are:

WIS = 249.84 µm

WIB = 81.3 µm

OA = 149.88˚

D = 29.90 µm

ATI = 82.61˚

**Engraving 42**

|  |  |
| --- | --- |

Figure SI1-42: a) 3D Alicona image of the incised surface of the human radius. The blue line indicates where the profile measurements were taken; b-c) profiles of the incision with and without measurements.

This is a large single incision produced by a to-and-fro sawing action, with two sub-incisions that overlap perfectly. It is 2.41 mm long overall and the values of the profile measurements, taken at the incision’s midpoint, are:

WIS = 306.10 µm

WIB = 106.21 µm

OA = 150.78˚

D = 33.56 µm

ATI = 90.13˚

**Engraving 43**

|  |  |
| --- | --- |

Figure SI1-43: a) 3D Alicona image of the incised surface of the human radius. The blue line indicates where the profile measurements were taken; b-c) profiles of the incision with and without measurements.

This is a single-stroke incision, 2.12 mm long. The values of the profile measurements, taken at the incision’s midpoint, are:

WIS = 212.77 µm

WIB = 40.72 µm

OA = 148.07˚

D = 32.09 µm

ATI = 90.62˚

**Engraving 44**

|  |  |
| --- | --- |

Figure SI1-44: a) 3D Alicona image of the incised surface of the human radius. The blue line indicates where the profile measurements were taken; b-c) profiles of the incision with and without measurements.

This is a large single-stroke incision, 2.89 mm long. The values of the profile measurements, taken at the incision’s midpoint, are:

WIS = 330.75 µm

WIB = 91.43 µm

OA = 143.86˚

D = 33.76 µm

ATI = 97.70˚

**Engraving 45**

|  |  |
| --- | --- |

Figure SI1-45: a) 3D Alicona image of the incised surface of the human radius. The blue line indicates where the profile measurements were taken; b-c) profiles of the incision with and without measurements.

This is a very large and deep incision produced by scraping. It is possible to recognise two internal sub-incisions that are parallel and close to each other. The incision has been divided into sub-incisions A (toward the proximal epiphysis) and B (toward the distal epiphysis).The total width of the double incision is 779.93 µm, sub-incision A is 1.59 mm long and sub-incision B is 1.40 mm long. The values of the profile measurements, taken at the incision’s midpoint, are:

WIB = 64.56 µm and 247.37 µm

OA = 160.71˚ and 151.68˚

D = 41.90 µm and 54.70 µm

ATI = 87.53˚ and 82.29˚

**Engraving 46**

|  |  |
| --- | --- |

Figure SI1-46: a) 3D Alicona image of the incised surface of the human radius. The blue line indicates where the profile measurements were taken; b-c) profiles of the incision with and without measurements.

This is a large though shallow double incision, produced by a to-and-fro sawing action. The two incisions are parallel and very close to each other, so much as to overlap for much of their lengths. The incision has been divided into sub-incisions A (toward the proximal epiphysis) and B (toward distal epiphysis). The total width of the double incision is 350.65 µm, sub-incision A is 1.11 mm long and sub-incision B is 1.16 mm long. The values of the profile measurements, taken at the incision’s midpoint, are:

WIB = 32.85 µm and 48.20 µm

OA = 168.75˚ and 162.56˚

D = 10.01 µm and 11.20 µm

ATI = 86.13˚ and 84.47˚

**Engraving 47**

|  |  |
| --- | --- |

Figure SI1-47: a) 3D Alicona image of the incised surface of the human radius. The blue line indicates where the profile measurements were taken; b-c) profiles of the incision with and without measurements.

This is a double incision produced by a to-and-fro action, where the two sub-incisions are parallel and very close to each other. The incision has been divided into sub-incisions A (toward the proximal epiphysis) and B (toward distal epiphysis). Sub-incision A is much more narrow and shallow than sub-incision B. It is possible that this sub-incision is in fact a scratch produced by an imperfection of the tool (shoulder effect), rather than being a proper incision. The total width of the double incision is 322.22 µm, sub-incision A is 1.67 mm long and sub-incision B is 2.01 mm long. The values of the profile measurements, taken at the incision’s midpoint, are:

WIB = 32.89 µm and 48.20 µm

OA = 137.30˚ and 151.42˚

D = 15.76 µm and 20.57 µm

ATI = 77.94˚ and 88.44˚

**Engraving 48**

|  |  |
| --- | --- |

Figure SI1-48: a) 3D Alicona image of the incised surface of the human radius. The blue line indicates where the profile measurements were taken; b-c) profiles of the incision with and without measurements.

This is a very large and deep incision, 2.05 mm long, produced by scraping. The scraping action must have been very homogenous, resulting in a unique single incision. Laterally, this incision has a fan-shape. The values of the profile measurements, taken at the incision’s midpoint, are:

WIS = 659.36 µm

WIB = 214.43 µm

OA = 159.92˚

D = 47.96 µm

ATI = 93.66˚

**Engraving 49**

|  |  |
| --- | --- |

Figure SI1-49: a) 3D Alicona image of the incised surface of the human radius. The blue line indicates where the profile measurements were taken; b-c) profiles of the incision with and without measurements.

This is a large and deep double incision produced by scraping. The pressure was not homogeneous, resulting in two successive sub-incisions that slightly diverge from each other, producing an overall fan-shape. The incision has been divided in sub-incisions A (toward the proximal epiphysis) and B (toward distal epiphysis). The total width of the incision is 656.16 µm, sub-incision A is 1.55 mm long and sub-incision B is 2.02 mm long. The values of the profile measurements, taken at the incision’s midpoint, are:

WIB = 80.78 µm and 94.86 µm

OA = 160.30˚ and 150.68˚

D = 35.06 µm and 53.63 µm

ATI = 88.83˚ and 91.24˚

**Engraving 50**

|  |  |
| --- | --- |

Figure SI1-50: a) 3D Alicona image of the incised surface of the human radius. The blue line indicates where the profile measurements were taken; b-c) profiles of the incision with and without measurements.

This is a single-stroke incision, 1.04 mm long. The values of the profile measurements, taken at the incision’s midpoint, are:

WIS = 185.4 µm

WIB = 56.96 µm

OA = 148.87˚

D = 22.07 µm

ATI = 91.62˚

**Engraving 51**

|  |  |
| --- | --- |

Figure SI1-51: a) 3D Alicona image of the incised surface of the human radius. The blue line indicates where the profile measurements were taken; b-c) profiles of the incision with and without measurements.

This is a large single-stroke incision, 2.06 mm long. The values of the profile measurements, taken at the incision’s midpoint, are:

WIS = 340.73 µm

WIB = 95.27 µm

OA = 151.94˚

D = 29.46 µm

ATI = 95.39˚

**Engraving 52**

|  |  |
| --- | --- |

Figure SI1-52: a) 3D Alicona image of the incised surface of the human radius. The blue line indicates where the profile measurements were taken; b-c) profiles of the incision with and without measurements.

This is a single-stroke incision, 1.93 mm long. The values of the profile measurements, taken at the incision’s midpoint, are:

WIS = 366.58 µm

WIB = 113.92 µm

OA = 149.42˚

D = 41.86 µm

ATI = 89.23˚

**Engraving 53**

|  |  |
| --- | --- |

Figure SI1-53: a) 3D Alicona image of the incised surface of the human radius. The blue line indicates where the profile measurements were taken; b-c) profiles of the incision with and without measurements.

This is a single-stroke incision, 1.93 mm long, very similar in its morphometric characteristics to incision 52. The values of the profile measurements, taken at the incision’s midpoint, are:

WIS = 280.90 µm

WIB = 103.92 µm

OA = 146.94˚

D = 36.48 µm

ATI = 89.04˚

**Engraving 54**

|  |  |
| --- | --- |

Figure SI1-54: a) 3D Alicona image of the incised surface of the human radius. The blue line indicates where the profile measurements were taken; b-c) profiles of the incision with and without measurements.

This is a very large and deep incision produced by scraping. The entire cutting edge was used homogenously, which does not allow for recognition of internal sub-incisions, and resulting in a lateral ‘rectangular shape’. The incision is 2.41 mm long and the values of the profile measurements, taken at the incision’s midpoint, are:

WIS = 1227.7 µm

WIB = 499.55 µm

OA = 155.45˚

D = 82.71 µm

ATI = 83.92˚

**Engraving 55**

|  |  |
| --- | --- |

Figure SI1-55: a) 3D Alicona image of the incised surface of the human radius. The blue line indicates where the profile measurements were taken; b-c) profiles of the incision with and without measurements.

This is a large though shallow incision produced by scraping. The scraping was not homogenous, and this resulted in clear internal sub-incisions. It is possible to distinguish at least three internal sub-incisions, which have been identified as A (toward the proximal epiphysis), B (intermediate), and C (toward distal epiphysis). The total width of the incision is 502.03 µm, sub-incision A is 1.78 mm long, sub-incision B is 1.58 mm long, and sub-incision C is 1.25 mm long. The values of the profile measurements, taken at the incision’s midpoint, are:

WIB = 20.17 µm, 65.58 µm and 75.05 µm

OA = 169.40˚, 170.23˚ and 169.33˚

D = 8.43 µm, 12.39 µm and 10.81 µm

ATI = 86.60˚, 87.87˚ and 89.65˚

**Engraving 56**

|  |  |
| --- | --- |

Figure SI1-56: a) 3D Alicona image of the incised surface of the human radius. The blue line indicates where the profile measurements were taken; b-c) profiles of the incision with and without measurements.

This is a very large single incision produced by a to-and-fro sawing action. No measurable internal sub-incisions were identified. The incision is 1.31 mm long and the values of the profile measurements, taken at the incision’s midpoint, are:

WIS = 317.99 µm

WIB = 114.57 µm

OA = 166.43˚

D = 13.34 µm

ATI = 86.47˚

**Engraving 57**

|  |  |
| --- | --- |

Figure SI1-49: a) 3D Alicona image of the incised surface of the human radius. The blue line indicates where the profile measurements were taken; b-c) profiles of the incision with and without measurements.

This is a double incision produced by a to-and-fro sawing action. The midpoint and the end of the first incision (toward the distal epiphysis, bottom of the image) overlap, while the end of the second incision (toward the proximal end of the radius, top of the image) diverges, which results in a fan-shape incision. The ATIs of sub-incision A (toward the proximal epiphysis) and sub-incision B (toward distal epiphysis) indicate that the angle of the tool (and tool user hand) had changed from ~80˚ for the first incision to over 100˚ for the second. This suggests the engraver intended to enlarge the incision. Both incisions are cut-off by the breakage in the bone; therefore the length of the incision does not reflect its full length. The width of the double incision is 389.16 µm, sub-incision A is 1.16 mm long and sub-incision B is 1.50 mm long. The values of the profile measurements, taken at the incision’s midpoint, are:

WIB = 50.97 µm and 92.61 µm

OA = 143.17˚ and 137.69˚

D = 24.97 µm and 40.83 µm

ATI = 82.69˚ and 100.45˚

**Engraving 58**

|  |  |
| --- | --- |

Figure SI1-58: a) 3D Alicona image of the incised surface of the human radius. The blue line indicates where the profile measurements were taken; b-c) profiles of the incision with and without measurements.

This is a large and deep incision, produced by scraping. The pressure on the stone tool edge was probably homogenous and therefore it is not possible to recognise internal sub-incisions. Overall, the shape of the incision is rectangular. The total length of the incision is 2.16 mm and the values of the profile measurements, taken at the incision’s midpoint, are:

WIS = 416.75 µm

WIB = 217.83 µm

OA = 137.31˚

D = 51.25 µm

ATI = 85.58˚

**Engraving 59**

|  |  |
| --- | --- |

Figure SI1-59: a) 3D Alicona image of the incised surface of the human radius. The blue line indicates where the profile measurements were taken; b-c) profiles of the incision with and without measurements.

This is a single-stroke incision intersected by incision 58 at its end. The total length of the incision is 2.16 mm. The values of the profile measurements, taken at the incision’s midpoint, are:

WIS = 230.72 µm

WIB = 69.27 µm

OA = 141.39˚

D = 25.01 µm

ATI = 98.29˚

**Engraving 60**

|  |  |
| --- | --- |

Figure SI1-60: a) 3D Alicona image of the incised surface of the human radius. The blue line indicates where the profile measurements were taken; b-c) profiles of the incision with and without measurements.

This is a large and deep incision, produced by scraping. The pressure of the stone tool was uniform, and no internal sub-incisions have been identified. However, the incision has a lateral fan-shape, suggesting the tool pivoted around one of the corners of its cutting edge during scraping. For this reason, its width changes, with a minimum length of 1.83 mm towards the distal end of the radius, and a maximum length of 3.76 mm toward the proximal end of the radius. The incision is, at its mid-point, 2.12 mm long and the values of the profile measurements, also taken at the incision’s midpoint, are:

WIS = 1119.45 µm

WIB = 253.98 µm

OA = 147.96˚

D = 123.17 µm

ATI = 84.19˚

**Engraving 61**

|  |  |
| --- | --- |

Figure SI1-61: a) 3D Alicona image of the incised surface of the human radius. The blue line indicates where the profile measurements were taken; b-c) profiles of the incision with and without measurements.

This is a single incision, produced by scraping. The bifurcating end of the incision (on the right) and its irregular starting point (to the left of the image) suggest the tool pivoted around one of the corner of its cutting edge, resulting in a fan-shape incision. The incision is 1.95 mm long. The values of the profile measurements, taken at the incision’s midpoint, are:

WIS = 334.53 µm

WIB = 105.66 µm

OA = 150.13˚

D = 41.52 µm

ATI = 89.29˚

**Engraving 62**

|  |  |
| --- | --- |

Figure SI1-62: a) 3D Alicona image of the incised surface of the human radius. The blue line indicates where the profile measurements were taken; b-c) profiles of the incision with and without measurements.

This is a single-stroke incision, probably produced by a unilaterally retouched blade. The incision is 1.49 mm long and the values of the profile measurements, taken at the incision’s midpoint, are:

WIS = 340.39 µm

WIB = 56.39 µm

OA = 165.35˚

D = 19.03 µm

ATI = 92.39˚

**Engraving 63**

|  |  |
| --- | --- |

Figure SI1-63: a) 3D Alicona image of the incised surface of the human radius. The blue line indicates where the profile measurements were taken; b-c) profiles of the incision with and without measurements.

55

This is a large and deep incision produced by scraping. Similarly to incision 55, it is possible to distinguish at least three internal sub-incisions. These have been defined as A (toward the proximal epiphysis), B (intermediate), and C (toward distal epiphysis). The total width of the multiple incision is 954.23 µm, sub-incision A is 2.85 mm long and sub-incisions B and C are both 2.60 mm long. The values of the profile measurements, taken at the incision’s midpoint, are:

WIB = 87.35 µm, 54.06 µm and 34.88 µm

OA = 146.76˚, 149.93˚ and 150.30˚

D = 109.94 µm, 97.68 µm and 58.38 µm

ATI = 87.27˚, 87.81˚ and 88.51˚

**Engraving 64**

|  |  |
| --- | --- |

Figure SI1-64: a) 3D Alicona image of the incised surface of the human radius. The blue line indicates where the profile measurements were taken; b-c) profiles of the incision with and without measurements.

This is a double incision, where the two incisions are more or less parallel to each other, but have been produced by a divergent to-and-fro sawing action (cf. Bello et al., 2013). The ATIs of sub-incision A (toward the proximal epiphysis) and sub-incision B (toward distal epiphysis) indicate that the angle of the tool (and the user’s hand) had changed from ~80˚ for the first incision to greater than 90˚ for the second, which allowed for the enlargement of the incision. The total width of the double incision is 453.55 µm, sub-incision A is 2.44 mm long and sub-incision B is 2.10 mm long. The values of the profile measurements, taken at the incision’s midpoint, are:

WIB = 107.7 µm and 24.28 µm

OA = 144.05˚ and 156.19˚

D = 31.93 µm and 25.62 µm

ATI = 82.56˚ and 91.56˚

**Engraving 65**

|  |  |
| --- | --- |

Figure SI1-65: a) 3D Alicona image of the incised surface of the human radius. The blue line indicates where the profile measurements were taken; b-c) profiles of the incision with and without measurements.

This is a large and deep incision produced by scraping. The incision has an overall fan-shape and is, on average, 2.12 mm long, with a minimum length of 1.96 mm toward the distal end of the radius, and a maximum length of 3.89 mm toward the proximal end. It must have been produced by a homogeneous scraping action as it is not possible to identify internal sub-incisions. The values of the profile measurements, taken at the incision’s midpoint, are:

WIS = 1043.93 µm

WIB = 232.39 µm

OA = 100.59˚

D = 120.42 µm

ATI = 59.37˚

**Engraving 66**

|  |  |
| --- | --- |

Figure SI1-66: a) 3D Alicona image of the incised surface of the human radius. The blue line indicates where the profile measurements were taken; b-c) profiles of the incision with and without measurements.

This is a single-stroke incision, 1.30 mm long. The values of the profile measurements, taken at the incision’s midpoint, are:

WIS = 227.23 µm

WIB = 76.52 µm

OA = 138.06˚

D = 21.51 µm

ATI = 102.4˚

**Engraving 67**

|  |  |
| --- | --- |

Figure SI1-67: a) 3D Alicona image of the incised surface of the human radius. The blue line indicates where the profile measurements were taken; b-c) profiles of the incision with and without measurements.

This is a double incision, produced by a to-and-fro sawing action, with the two incisions more or less parallel to each other, but produced by divergent action (cf. Bello et al., 2013). The ATIs of sub-incision A (toward the proximal epiphysis) and sub-incision B (toward distal epiphysis) indicate that the angle of the tool (and the user’s hand) had changed from ~87˚ for the first incision to greater than 95˚ for the second. The total width of the double incision is 302.00 µm, sub-incision A is 0.97 mm long and sub-incision B is 1.34 mm long. The values of the profile measurements, taken for incisions 67 A and B at the incision’s midpoint, are:

WIB = 39.15 µm and 36.57 µm

OA = 155.14˚ and 130.59˚

D = 25.63 µm and 26.92 µm

ATI = 87.17˚ and 95.24˚

**Engraving 68**

|  |  |
| --- | --- |

Figure SI1-68: a) 3D Alicona image of the incised surface of the human radius. The blue line indicates where the profile measurements were taken; b-c) profiles of the incision with and without measurements.

This is a double incision produced by scraping. The pressure on the stone tool’s edge was not homogeneous and this resulted in the production of two consecutive and distinct sub-incisions. The incision has an overall fan-shape, and the length of the two distinct sub-incisions, A (toward the proximal epiphysis and B (toward distal epiphysis) are 1.31 mm and 2.22 mm, respectively. The overall width (WIS) is 739.84 µm and the values of the profile measurements, taken at the incision’s midpoint, are:

WIB = 59.58 µm and 102.25 µm

OA = 128.64˚ and 131.23˚

D = 47.38 µm and 69.63 µm

ATI = 70.14˚ and 73.01˚

**Engraving 69**

|  |  |
| --- | --- |

Figure SI1-69: a) 3D Alicona image of the incised surface of the human radius. The blue line indicates where the profile measurements were taken; b-c) profiles of the incision with and without measurements.

This is a large single-stroke incision, 1.97 mm long, probably produced by a unilaterally retouched flake/blade. The values of the profile measurements, taken at the incision’s midpoint, are:

WIS = 256.06 µm

WIB = 69.70 µm

OA = 162.04˚

D = 17.02 µm

ATI = 87.18˚

**Engraving 70**

|  |  |
| --- | --- |

Figure SI1-70: a) 3D Alicona image of the incised surface of the human radius. The blue line indicates where the profile measurements were taken; b-c) profiles of the incision with and without measurements.

This is a large and deep incision, produced by a to-and-fro sawing action. The sub-incisions almost overlap perfectly at their mid-point, at the end of the first sub-incision (toward the distal epiphysis, bottom of the image), and at the beginning of second sub-incision (toward the proximal epiphysis, top of the image). However, the start point of the first incision and the end point of the second incision clearly diverge. The incision is 2.15 mm long and the values of the profile measurements, taken at the incision’s midpoint (where the two sub-incisions overlap), are:

WIS = 428.11 µm

WIB = 176.25 µm

OA = 151.18˚

D = 50.29 µm

ATI = 90.01˚

**Engraving 71**

|  |  |
| --- | --- |

Figure SI1-71: a) 3D Alicona image of the incised surface of the human radius. The blue line indicates where the profile measurements were taken; b-c) profiles of the incision with and without measurements.

This is a large incision produced by a short to-and-fro sawing action. No internal sub-incisions are recognisable, however the to-and-fro action is confirmed by the bifurcating ends of the incision. The incision is 1.24 mm long and the values of the profile measurements, taken at the incision’s midpoint, are:

WIS = 396.96 µm

WIB = 97.66 µm

OA = 139.91˚

D = 60.64 µm

ATI = 92.58˚

**Engraving 72**

|  |  |
| --- | --- |

Figure SI1-72: a) 3D Alicona image of the incised surface of the human radius. The blue line indicates where the profile measurements were taken; b-c) profiles of the incision with and without measurements.

This is a large and deep single incision produced by scraping. The action must have been with a certain amount of strength because it appears as though the tool was wedged in the bone at the end of the scraping action. The incision has an overall fan-shape and is, on average, 2.42 mm long with a minimum length of 1.90 mm towards the distal end of the radius and a maximum length of 3.71 mm toward the proximal end of the radius. The values of the profile measurements, taken at the incision’s midpoint, are:

WIS = 549.78 µm

WIB = 102.73 µm

OA = 86.72˚

D = 132.75 µm

ATI = 61.17˚

**Engraving 73**

|  |  |
| --- | --- |

Figure SI1-73: a) 3D Alicona image of the incised surface of the human radius. The blue line indicates where the profile measurements were taken; b-c) profiles of the incision with and without measurements.

This is a large and deep single incision produced by scraping. The homogeneous action has left several ‘chatting-marks’ on the surface, but none deep or distinct enough to be defined as an internal sub-incision. The incision has an overall rectangular shape and its average length (taken at its midpoint) is 3.37 mm. The values of the profile measurements, taken at the incision’s midpoint, are:

WIS = 936.8 µm

WIB = 310.10 µm

OA = 148.98˚

D = 96.21 µm

ATI = 85.19˚

**Engraving 74**

|  |  |
| --- | --- |

Figure SI1-74: a) 3D Alicona image of the incised surface of the human radius. The blue line indicates where the profile measurements were taken; b-c) profiles of the incision with and without measurements.

This is a large incision produced by scraping. In this case it is possible to distinguish at least two internal sub-incisions. The successive incisions (sub-incision A, toward the proximal epiphysis, and sub-incision B, toward distal epiphysis) are parallel to each other and respectively 2.28 mm and 1.82 mm long. The overall width (WIS) is 613.96 µm and the values of the profile measurements, taken at the incision’s midpoint, are:

WIB = 116.92 µm and 48.23 µm

OA = 144.65˚ and 142.09˚

D = 68.38 µm and 37.88 µm

ATI = 91.33˚ and 92.73˚

**Engraving 75**

|  |  |
| --- | --- |

Figure SI1-75: a) 3D Alicona image of the incised surface of the human radius. The blue line indicates where the profile measurements were taken; b-c) profiles of the incision with and without measurements.

This is a large and deep incision produced by scraping. It is not possible to distinguish measurable internal sub-incisions, which suggests a homogeneous scraping action. The incision has an overall fan-shape, and its average length (taken at the incision’s midpoint) is 3.31 mm. The values of the profile measurements, taken at the incision’s midpoint, are:

WIS = 633.36 µm

WIB = 78.79 µm

OA = 143.84˚

D = 81.88 µm

ATI = 83.15˚

**Engraving 76**

|  |  |
| --- | --- |

Figure SI1-76: a) 3D Alicona image of the incised surface of the human radius. The blue line indicates where the profile measurements were taken; b-c) profiles of the incision with and without measurements.

This is a single incision, which is the result of consecutive multiple incisions produced by scraping. The hand pressure was probably homogeneous because the profile analysis does not reveal any clear distinguishable internal sub-incisions. The incision has an overall fan-shape and is, on average, 2.42 mm long, with a minimum length of 1.92 mm towards the distal end of the radius and a maximum length of 3.74 mm toward the proximal end of the radius. The values of the profile measurements, taken at the incision’s midpoint, are:

WIS = 766.56 µm

WIB = 192.28 µm

OA = 138.51˚

D = 129.93 µm

ATI = 89.71˚

**Engraving 77**

|  |  |
| --- | --- |

Figure SI1-77: a) 3D Alicona image of the incised surface of the human radius. The blue line indicates where the profile measurements were taken; b-c) profiles of the incision with and without measurements.

This is a double incision, which is the result of a to-and-fro sawing action. The internal sub-incisions (sub-incision A, toward the proximal epiphysis, and sub-incision B, toward distal epiphysis) are parallel to each other and 2.20 mm and 2.23 mm long, respectively. The overall width (WIS) is 339.64 µm and the values of the profile measurements, taken at the incision’s midpoint, are:

WIB = 36.51 µm and 43.17 µm

OA = 130.88˚ and 136.95˚

D = 37.64 µm and 44.79 µm

ATI = 81.45˚ and 85.43˚

**Engraving 78**

|  |  |
| --- | --- |

Figure SI1-78: a) 3D Alicona image of the incised surface of the human radius. The blue line indicates where the profile measurements were taken; b-c) profiles of the incision with and without measurements.

This is a single-stroke incision, separated by 2.07 mm from the previous group of incisions (incisions 73-77). This incision is 1.47 mm long and the values of the profile measurements, taken at the incision’s midpoint, are:

WIS = 189.41 µm

WIB = 58.69 µm

OA = 156.94˚

D = 17.98 µm

ATI = 87.77˚

**Engraving 79**

|  |  |
| --- | --- |

Figure SI1-79: a) 3D Alicona image of the incised surface of the human radius. The blue line indicates where the profile measurements were taken; b-c) profiles of the incision with and without measurements.

This is a double incision, which is the result of a to-and-fro sawing action. The internal sub-incisions (sub-incision A, toward the proximal epiphysis, and sub-incision B, toward distal epiphysis) are parallel to each other and 2.04 mm and 4.14 mm long, respectively. The overall width (WIS) is 546.45 µm and the values of the profile measurements, taken at the incision’s midpoint, are:

WIB = 49.23 µm and 78.94 µm

OA = 120.29˚ and 150.29˚

D = 17.25 µm and 36.29 µm

ATI = 96.04˚ and 95.39˚

**Engraving 80**

|  |  |
| --- | --- |

Figure SI1-80: a) 3D Alicona image of the incised surface of the human radius. The blue line indicates where the profile measurements were taken; b-c) profiles of the incision with and without measurements.

This is a single incision, which is the result of multiple overlapping sub-incisions produced by a to-and-fro action. The incision is 2.93 mm long and the values of the profile measurements, taken at the incision’s midpoint, are:

WIS = 465.05 µm

WIB = 100.41 µm

OA = 150.17˚

D = 56.53 µm

ATI = 91.04˚

**Engraving 81**

|  |  |
| --- | --- |

Figure SI1-81: a) 3D Alicona image of the incised surface of the human radius. The blue line indicates where the profile measurements were taken; b-c) profiles of the incision with and without measurements.

This is a double incision, which is the result of a to-and-fro sawing action. The internal sub-incisions (sub-incision A, toward the proximal epiphysis, and sub-incision B, toward distal epiphysis) are parallel to each other and 1.96 mm and 2.52 mm long, respectively. The overall width (WIS) is 418.88 µm and the values of the profile measurements, taken at the incision’s midpoint, are:

WIB = 28.27 µm and 36.76 µm

OA = 150.74˚ and 142.41˚

D = 22.58 µm and 35.44 µm

ATI = 92.98˚ and 89.71˚

**Engraving 82**

|  |  |
| --- | --- |

Figure SI1-82: a) 3D Alicona image of the incised surface of the human radius. The blue line indicates where the profile measurements were taken; b-c) profiles of the incision with and without measurements.

This is a large incision produced by a to-and-fro sawing action. Two sub-incisions have been identified: sub-incision A (toward the proximal epiphysis) and sub-incision B (toward distal epiphysis). These are parallel to each other and 2.71 mm and 2.23 mm long, respectively. The overall width (WIS) is 467.90 µm and the values of the profile measurements, taken at the incision’s midpoint, are:

WIB = 82.07 µm and 46.85 µm

OA = 116.29˚ and 140.75˚

D = 45.43 µm and 31.39 µm

ATI = 68.53˚ and 82.24˚

**Engraving 83**

|  |  |
| --- | --- |

Figure SI1-83: a) 3D Alicona image of the incised surface of the human radius. The blue line indicates where the profile measurements were taken; b-c) profiles of the incision with and without measurements.

This is a large incision produced by a homogeneous scraping action, which has left no measurable sub-incisions. The incision, which is separated from incision 82 by a small gap (0.68 mm), has an overall fan-shape and is, on average, 1.87 mm long, with a minimum length of 1.49 mm towards the distal end of the radius and a maximum length of 2.67 mm toward the proximal end of the radius. The values of the profile measurements, taken at the incision’s midpoint, are:

WIS = 613.52 µm

WIB = 270.69 µm

OA = 145.39˚

D = 39.95 µm

ATI = 78.52˚

**Engraving 84**

|  |  |
| --- | --- |

Figure SI1-84: a) 3D Alicona image of the incised surface of the human radius. The blue line indicates where the profile measurements were taken; b-c) profiles of the incision with and without measurements.

This is a double incision produced by scraping. The incision has an overall fan-shape, and two distinct sub-incisions (sub-incision A, toward the proximal epiphysis, and sub-incision B, toward distal epiphysis) have been identified. They are 1.97 mm and 1.87 mm long, respectively. The overall width (WIS) is 522.77 µm and the values of the profile measurements, taken at the incision’s midpoint, are:

WIB = 81.84 µm and 42.82 µm

OA = 153.54˚ and 155.88˚

D = 47.63 µm and 20.68 µm

ATI = 90.56˚ and 94.34˚

**Engraving 85**

|  |  |
| --- | --- |

Figure SI1-85: a) 3D Alicona image of the incised surface of the human radius. The blue line indicates where the profile measurements were taken; b-c) profiles of the incision with and without measurements.

This is a large and deep incision, produced by scraping. The pressure was probably homogeneous as it is not possible to recognise distinct measurable sub-incisions within it. The incision has an overall fan-shape and is, on average, 2.45 mm long, with a minimum length of 1.27 mm towards the distal end of the radius and a maximum length of 3.53 mm toward the proximal end of the radius. The values of the profile measurements, taken at the incision’s midpoint, are:

WIS = 804.25 µm

WIB = 213.76 µm

OA = 139.88˚

D = 124.54 µm

ATI = 86.85˚

**Engraving 86**

|  |  |
| --- | --- |

Figure SI1-86: a) 3D Alicona image of the incised surface of the human radius. The blue line indicates where the profile measurements were taken; b-c) profiles of the incision with and without measurements.

This is a large and deep incision produced by scraping. Similarly to the previous incision, the pressure exerted by the tool was probably homogeneous as it is not possible to distinguish distinct measurable sub-incisions. The incision has an overall fan-shape and is 1.85 mm long at the midpoint of the incision, with a minimum length of 1.75 mm towards the distal end of the radius and a maximum length of 2.47 mm toward the proximal end of the radius. The values of the profile measurements, taken at the incision’s midpoint, are:

WIS = 469.97 µm

WIB = 277.10 µm

OA = 120.39˚

D = 62.0 µm

ATI = 76.53˚

**Engraving 87**

|  |  |
| --- | --- |

Figure SI1-87: a) 3D Alicona image of the incised surface of the human radius. The blue line indicates where the profile measurements were taken; b-c) profiles of the incision with and without measurements.

This is a double incision produced by a to-and fro action. It is possible to recognise two distinct sub-incisions within it, sub-incision A, toward the proximal epiphysis, and sub-incision B, toward distal epiphysis. They are 2.35 and 3.05 mm long, respectively. The overall width (WIS) is 570.91 µm and the values of the profile measurements, taken at the incision’s midpoint, are:

WIB = 209.72 µm and 74.72 µm

OA = 151.25˚ and 112.82˚

D = 45.80 µm and 55.33 µm

ATI = 85.72˚ and 82.03˚
